# Changing epidemiology of calcific aortic valve disease: 30-year trends of incidence, prevalence, and deaths across 204 countries and territories

**DOI:** 10.18632/aging.202942

**Published:** 2021-05-11

**Authors:** Bin Yi, Weike Zeng, Lei Lv, Ping Hua

**Affiliations:** 1 Department of Cardiovascular Surgery, Sun Yat-Sen Memorial Hospital, Sun Yat-Sen University, Guangzhou 510120, China; 2 Department of Radiology, Sun Yat-Sen Memorial Hospital, Sun Yat-Sen University, Guangzhou 510120, China

**Keywords:** epidemiology, calcific aortic valve disease, 30-year trends, incidence, prevalence

## Abstract

Calcific aortic valve disease (CAVD) is associated with increased morbidity and mortality. We aimed to elucidate the 30-year epidemiology of CAVD globally. Global CAVD incidence, prevalence, and deaths increased 3.51-, 4.43-, and 1.38-fold from 1990 to 2019, respectively, without any decreasing trends, even after age standardization. In 2019, Slovenia had the highest age-standardized rate (ASR) of CAVD incidence (62.21/100,000 persons) and prevalence (1,080.06/100,000) whereas Cyprus had the highest ASR of deaths (8.20/100,000). Population aging was an important contributor to incidence. Compared with women, more men had CAVD and men had earlier peaks in disease prevalence. High systolic blood pressure, diet high in sodium, and lead exposure were the main risk factors for deaths owing to CAVD. The estimated annual percentage change, a measure to estimate the variation of ASR, was significantly associated with the ASR and sociodemographic index (SDI) in 2019 for incidence and prevalence across all 204 countries and territories (all p<0.0001). With increased lifespan and risk factors, the overall burden of CAVD is high and remains on the rise, with differences by sex, age, and SDI level. Our findings serve to sound the alarm for organizations, institutions, and resources whose primary purpose is to improve human health.

## INTRODUCTION

Calcific aortic valve disease (CAVD) is an active progressive disease characterized by an abnormal accumulation of calcium nodules on the aortic valve or annulus. CAVD is of clinical importance in that severe mineralization results in arrhythmia, thromboembolism, left ventricular dysfunction, and even sudden death [1–3]. Given the lack of effective pharmacological strategies for remission and recovery in CAVD, the only feasible therapy choice is open-heart or transcatheter aortic valve replacement [4], which places psychological and physiological burdens on patients and their family, as well as stresses the health care system and limited medical resources [5]. A class I indication for repair or replacement of the aortic valve is patients with symptoms and severe aortic stenosis, based on the American Heart Association/American College of Cardiology (AHA/ACC) practice guidelines [6]. Therefore, comprehensive understanding of the spatial and temporal patterns of CAVD has become critical in the prevention and control of CAVD.

The Global Burden of Disease Study (GBD) 2019, which represents an update of the GBD 2017, has facilitated systematic analyses regarding the global burden of 87 risk factors, 369 diseases, and injuries in 204 countries and territories from 1990 to 2019 [7, 8], as well as comprehensive demographic assessments on global age- and sex-specific fertility, mortality, healthy life expectancy, and population estimates from 1950 to 2019 [9]. In this study, we analyzed the epidemiology of CAVD across 204 countries and territories during the past 30 years by taking full advantage of estimates from the GBD 2019. Furthermore, we expect to offer more thorough information on the burden of CAVD, which will be beneficial and valuable in generating more tailored strategies for managing CAVD.

## RESULTS

### Incidence patterns of CAVD

**Figure 1:**
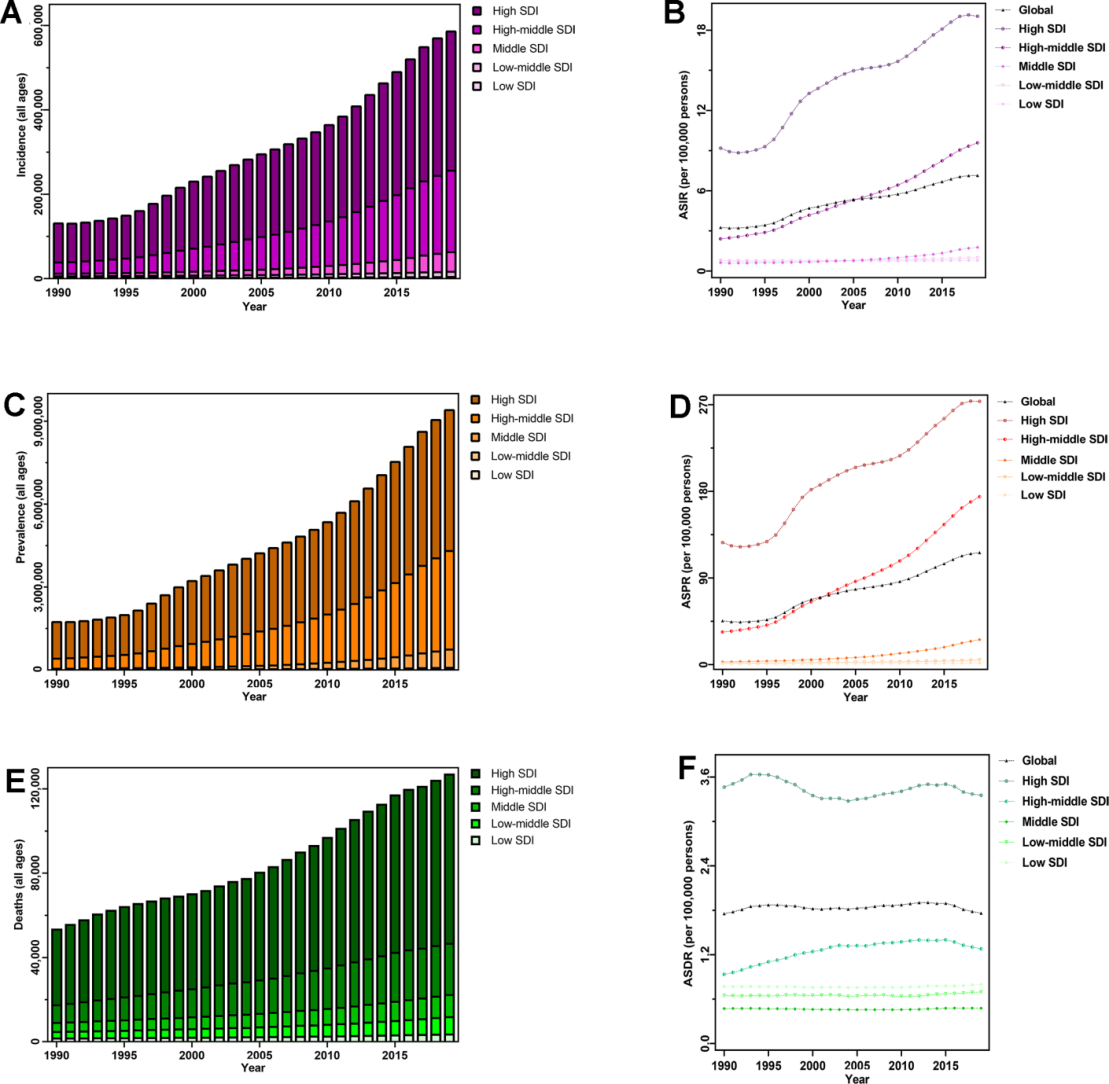
**The disease burden of CAVD globally, and in five SDI quintiles from 1990 to 2019.** (**A**) Incident cases; (**B**) ASIR; (**C**) prevalent cases; (**D**) ASPR; (**E**) deaths; (**F**) ASDR. Abbreviations: CAVD, calcific aortic valve disease; SDI, socio-demographic index; ASIR, age-standardized incidence rate; ASPR, age-standardized prevalence rate; ASDR, age-standardized deaths rate.

The global number of newly diagnosed cases of CAVD increased 3.51-fold from 1990, with 589,638 in 2019, including 313,805 male and 275,833 female patients. During the same period, the ASIR was stable (EAPC=2.74, 95% CI: −1.80–7.50) from 3.25/100,000 persons to 7.13/100,000 persons (Table 1 and Figure 1A, 1B).

**Table 1:** The spatio-temporal epidemic dynamics of CAVD in cases and ASR from 1990 to 2019.

**Characteristics**	**Incidence**			**Prevalence**			**Deaths**		
	**2019 Cases X10^3^** **(95% UI)**	**1990-2019 Change in case**	**1990-2019 EAPC in ASR (95% CI)**	**2019 Cases X10^3^** **(95% UI)**	**1990-2019 Change in case**	**1990-2019 EAPC in ASR (95% CI)**	**2019 Cases X10^3^ (95% UI)**	**1990-2019 Change in case**	**1990-2019 EAPC in ASR (95% CI)**
Global	589.64(512.9-677.06)	3.51	2.74(-1.8-7.5)	9404.08(8079.6-10889.73)	4.43	3.29(2.07-4.51)	126.83(105.6-141.39)	1.38	0.01(-6.94-7.49)
Sex									
Male	313.8(271.31-360.92)	3.51	2.74(-1.56-7.23)	5027.26(4276.88-5861.59)	4.62	3.36(2.22-4.51)	54.17(47.77-58.67)	1.21	-0.03(-6.8-7.25)
Female	275.83(239.87-317.14)	3.51	2.74(-2.08-7.8)	4376.82(3771.24-5082.8)	4.22	3.18(1.89-4.49)	72.65(57.76-84.3)	1.52	0.07(-7.13-7.82)
SDI Quintiles									
Low SDI	3.6(3.12-4.16)	1.22	-0.12(-10.22-11.11)	9.68(7.61-12.14)	2.3	1.41(-6.63-10.14)	3.46(2.56-4.39)	1.3	0.12(-10.12-11.54)
Low-middle SDI	12.5(10.74-14.48)	2.2	0.82(-9.04-11.75)	67.26(54.36-81.97)	6.18	4.2(-2.27-11.1)	8.17(6.63-9.99)	1.62	0.25(-10.79-12.65)
Middle SDI	46.68(38.85-55.46)	6.54	3.64(-6.15-14.46)	658.55(529.93-800.78)	20.59	7.96(3.47-12.63)	10.48(9.43-11.74)	1.45	0.04(-12.92-14.92)
High-middle SDI	193.16(163.48-225.58)	6.32	4.89(-0.11-10.13)	3569.82(3002.41-4203.73)	8.78	5.81(4.48-7.16)	24.44(20.86-27.26)	1.92	1.09(-7.79-10.83)
High SDI	329.82(285.98-381.25)	2.56	2.55(-0.2-5.37)	5095.44(4402.07-5933.38)	2.85	2.69(1.94-3.43)	80.21(64.3-90.1)	1.23	-0.11(-5.15-5.2)
GBD Regions									
Central Asia	2.65(2.18-3.11)	7.02	5.21(-4.08-15.39)	41.06(33.49-48.81)	9.9	6.79(4.08-9.58)	0.21(0.17-0.26)	2.26	3.05(-16.95-27.86)
East Asia	56.64(45.3-69.33)	18.07	7.27(-5.16-21.33)	891.02(707.25-1093.31)	47.76	10.92(5.75-16.35)	3.23(2.62-3.85)	1.06	-0.3(-20.39-24.85)
South Asia	12.37(10.49-14.62)	2	0.26(-9.01-10.48)	30.19(23.68-37.63)	2.62	1.44(-5.91-9.38)	8.75(6.85-11.05)	1.86	0.04(-10.37-11.67)
Southeast Asia	3.29(2.78-3.94)	3.62	1.84(-12.42-18.42)	23.99(18.74-30.27)	12.44	6.25(-2.65-15.97)	1.7(1.4-2.13)	1.59	0.34(-15.44-19.07)
High-income Asia Pacific	88.6(73.61-107.12)	1.91	1.75(-0.46-4.02)	1715.7(1450.88-2042.13)	2.65	1.95(1.38-2.51)	13.57(9.08-16.51)	2.17	-0.9(-7.03-5.65)
Australasia	20.17(17.13-23.51)	15.01	7.42(4.21-10.74)	320.82(272.23-381.06)	31.48	9.92(8.74-11.12)	1.87(1.51-2.11)	1.42	-0.31(-5.4-5.05)
Oceania	0.13(0.11-0.15)	3.64	1.68(-6.43-10.48)	1.13(0.9-1.37)	9.25	5.18(1.45-9.06)	0.04(0.03-0.06)	1.35	-0.16(-10.47-11.33)
High-income North America	123.57(106.45-142.33)	1.53	1.2(-1.14-3.59)	1492.89(1305.2-1727.5)	1.17	0.85(0.19-1.51)	27.19(22.16-30.24)	1.07	0.08(-4.84-5.25)
Caribbean	2.35(2.02-2.73)	4.31	3.42(-2.69-9.91)	45.47(37.69-54.85)	12.54	6.91(4.75-9.11)	0.52(0.42-0.63)	1.04	-0.01(-9.12-10)
Andean Latin America	2.15(1.86-2.46)	13.15	5.71(-3.1-15.32)	33.35(28.25-39.02)	39.13	9.95(6.07-13.97)	0.29(0.24-0.35)	1.46	-0.35(-12.37-13.33)
Central Latin America	5.75(4.95-6.62)	4.47	2.3(-5.23-10.43)	76.96(63.86-91.38)	10	4.89(2.12-7.73)	1.96(1.61-2.43)	1.98	0.18(-9.81-11.28)
Southern Latin America	8.73(7.73-9.92)	7.02	5(0.19-10.04)	101.89(87.12-120.8)	17.54	8.39(6.18-10.64)	2.84(2.46-3.16)	1.05	-0.14(-5.21-5.2)
Tropical Latin America	6.59(5.61-7.68)	2.38	0.68(-5.3-7.03)	58.6(47.23-72.05)	6.29	3.91(1.06-6.85)	3.58(3.11-4.14)	1.34	-0.52(-7.6-7.1)
Central Europe	57.28(48.87-67.32)	4.84	5.73(2.73-8.82)	1260.56(1067.65-1479.62)	7.15	6.27(5.51-7.04)	4.68(3.77-5.58)	4.18	4.04(-5.43-14.47)
Eastern Europe	68.42(55.23-82.91)	7.62	7.33(2.9-11.96)	1328.69(1065.7-1605.21)	9.92	7.93(6.77-9.1)	1.64(1.37-1.93)	1.92	2.85(-13.7-22.57)
Western Europe	116.51(98.95-138.35)	6.3	5.73(1.28-10.38)	1862.79(1577.56-2209.09)	7.54	6.01(4.75-7.29)	47.89(39.52-53.66)	1.27	0.42(-4.39-5.46)
North Africa and Middle East	7.07(5.98-8.35)	2.47	0.71(-7.35-9.47)	54.3(43.23-66.78)	4.6	2.76(-0.95-6.6)	3.82(3.19-4.44)	1.21	-0.53(-9.41-9.22)
Central Sub-Saharan Africa	0.41(0.36-0.47)	1.52	0.12(-9.2-10.39)	0.8(0.62-1.01)	2.24	1.09(-7.52-10.5)	0.41(0.29-0.58)	1.42	0.12(-9.06-10.22)
Eastern Sub-Saharan Africa	1.17(1.01-1.35)	1.33	0.02(-9.9-11.04)	2.6(2-3.27)	2.41	1.45(-7.33-11.05)	1.2(0.95-1.53)	1.08	-0.07(-9.49-10.34)
Southern Sub-Saharan Africa	4.64(3.72-5.7)	9.52	5.22(-0.79-11.6)	57.41(44.28-73.39)	23.27	8.83(6.18-11.55)	0.43(0.37-0.48)	0.96	0.21(-9.26-10.68)
Western Sub-Saharan Africa	1.16(0.99-1.37)	1.3	0.06(-11.82-13.54)	3.87(3-4.88)	1.98	0.9(-6.84-9.29)	1.02(0.71-1.42)	0.99	-0.3(-12.11-13.09)

Abbreviations: CAVD, calcific aortic valve disease; ASR, age standardized rate; SDI, socio-demographic index; GBD, global burden of disease UI, uncertainty interval; EAPC, estimated annual percentage change; CI, confidence interval.

**Figure 2:**
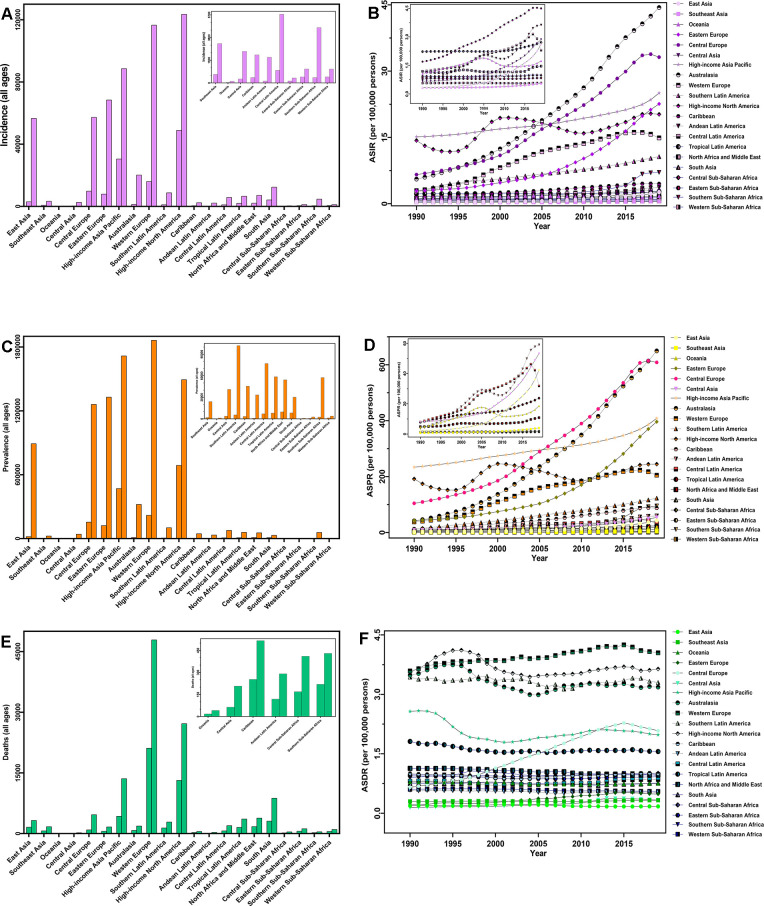
**The disease burden of CAVD in 21 GBD regions from 1990 to 2019.** (**A**) Incident cases; (**B**) ASIR; (**C**) prevalent cases; (**D**) ASPR; (**E**) deaths; (**F**) ASDR. Abbreviations: CAVD, calcific aortic valve disease; GBD, global burden of disease; ASIR, age-standardized incidence rate; ASPR, age-standardized prevalence rate; ASDR, age-standardized deaths rate.

In the five SDI quintiles, the ASIR remained basically unchanged from 1990 to 2019; however, CAVD presented a trend of more incident cases, from a 1.22-fold increase in low SDI quintiles to a 6.54-fold increase in middle quintiles. Moreover, in 2019, the highest numbers of incident cases (329,823) and ASIR (19.04/100,000 persons) were observed in the high SDI regions (Table 1 and Figure 1A, 1B). The incident cases of CAVD increased across the 21 GBD regions, and the largest change, an 18.07-fold increase, was recorded in East Asia. As for the ASIR, Australasia showed the largest increase (EAPC=7.42, 95% CI: 4.21–10.74). Furthermore, the most incident cases were observed in high-income North American countries (123,568) whereas Australasia had the highest ASIR (44.39/100,000 persons) in 2019 (Table 1 and Figure 2A, 2B).

**Figure 3:**
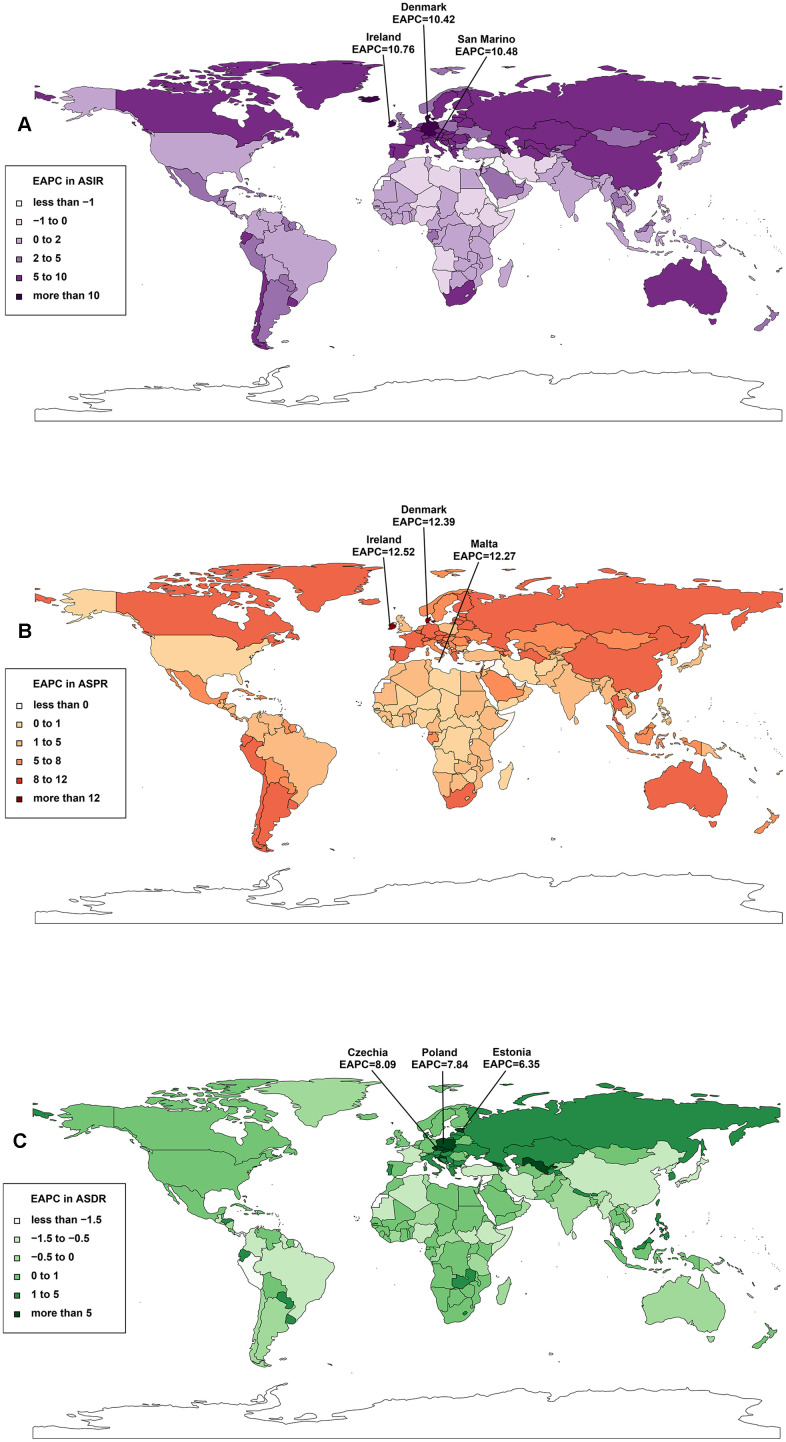
**The trends of CAVD for both sexes in 204 countries and territories from 1990 to 2019.** (**A**) The EAPC in ASIR; (**B**) the EAPC in ASPR; (**C**) the EAPC in ASDR. Abbreviatons: CAVD, calcific aortic valve disease; EAPC, estimated annual percentage change; ASIR, age-standardized incidence rate; ASPR, age-standardized prevalence rate; ASDR, age-standardized deaths rate.

The incidence of CAVD varied significantly from nation to nation. In 2019, the highest number of incident cases (117,080) was recorded in the United States, followed by Japan (71,837), China (54,965), and the Russian Federation (54,152) (Supplementary Tables 1, 2 and Supplementary Figure 1). Moreover, Slovenia, Hungary, and Romania had the highest ASIR, with 62.21/100,000 persons, 56.24/100,000 persons, and 54.92/100,000 persons, respectively (Supplementary Table 1 and Supplementary Figure 2). As for trends, Ireland and Andorra had the largest growth in ASIR (EAPC=10.76, 95% CI: 2.16–20.08) and cases (42.61-fold increase), respectively (Supplementary Table 1, Figure 3A and Supplementary Figure 3).

### Prevalence patterns of CAVD

Worldwide, 9,404,078 patients (male 5,027,261, female 4,376,817) had CAVD in 2019, a 4.43-fold increase from 1990. The ASPR also increased across the study period (EAP =3.29, 95% CI, 2.07–4.51), with 116.34/100,000 persons in 2019 (Table 1 and Figure 1C, 1D).

In 2019, low SDI quintiles had the lowest absolute value (9,675) and ASR (1.67/100,000 persons) of CAVD prevalence, with the highest value for the two metrics observed in high SDI quintiles (5,095,444 and 273.52/100,000 persons, respectively). Furthermore, the number of patients with CAVD increased in all SDI quintiles, with the largest increase observed in middle SDI quintiles (20.59-fold increase) whereas the ASPR increased in middle (EAPC=7.96, 95% CI: 3.47–12.63), high–middle (EAPC=5.81, 95% CI: 4.48–7.16), and high SDI quintiles (EAPC=2.69, 95% CI: 1.94–3.43) from 1990 to 2019 (Table 1 and Figure 1C, 1D). For the 21 GBD regions, the most significant increase was noticed in East Asia over the 30-year period, both in terms of cases (47.76-fold increase) and ASPR (EAPC=10.92, 95% CI: 5.75–16.35) (Table 1 and Figure 2C, 2D).

During the study, the temporal trend of the CAVD ASPR was significantly heterogeneous throughout the world, with the largest increases in Ireland (EAPC=12.52, 95% CI: 9.48–15.64), Denmark (EAPC=12.39, 95% CI: 8.93–15.96), and Malta (EAPC=12.27, 95% CI: 8.21–16.49) (Supplementary Table 3 and Figure 3B). The number of patients with CAVD increased across all 204 countries, with the most remarkable increase noted in Taiwan (Province of China) (64.05-fold increase), followed by Malta (63.03-fold increase), and Cyprus (62.05-fold increase). In 2019, the highest ASPR and highest number of CAVD patients in all age groups were in Slovenia (1,080.06/100,000 persons) and the United States (1,425,073), respectively (Supplementary Tables 2, 3 and Supplementary Figures 4–6).

### Mortality patterns of CAVD

The most CAVD deaths in 2019 were recorded in the United States, followed by Germany and Japan (248,256, 13,154, and 12,868, respectively). Cyprus, Slovenia, and Norway presented the largest ASDR with 8.20/100,000 persons, 6.77/100,000 persons, and 5.72/100,000 persons, respectively. Over the study period, the greatest change was a 14.88-fold increase in Poland (Supplementary Tables 2, 4 and Supplementary Figures 7–9). However, the ASDR was not significantly different among the 204 countries from 1990 to 2019 (Supplementary Table 4 and Figure 3C).

Since 1990, no SDI or GBD regions have shown a decreasing trend in CAVD deaths or ASDR (Table 1). In 2019, among the five SDI quintiles, the highest number of deaths (80,211) and highest ASDR (3.35/100,000 persons) were both identified in high SDI quintiles (Table 1 and Figure 1E, 1F). Across the 21 GBD regions, the number of deaths owing to CAVD increased from 41 in Oceania to 47,894 in Western Europe; the highest ASDR (4.05/100,000 persons) was also observed in Western Europe (Table 1 and Figure 2E, 2F).

Globally, a total of 126,827 patients (male 54,175 and female 72,652) died from CAVD in 2019, a 1.38-fold rise from 1990. Over all study years, the ASDR was relatively stable from 1.75/100,000 persons to 1.76/100,000 persons (Table 1 and Figure 2E, 2F).

### Decomposition analysis of incident cases

**Figure 4:**
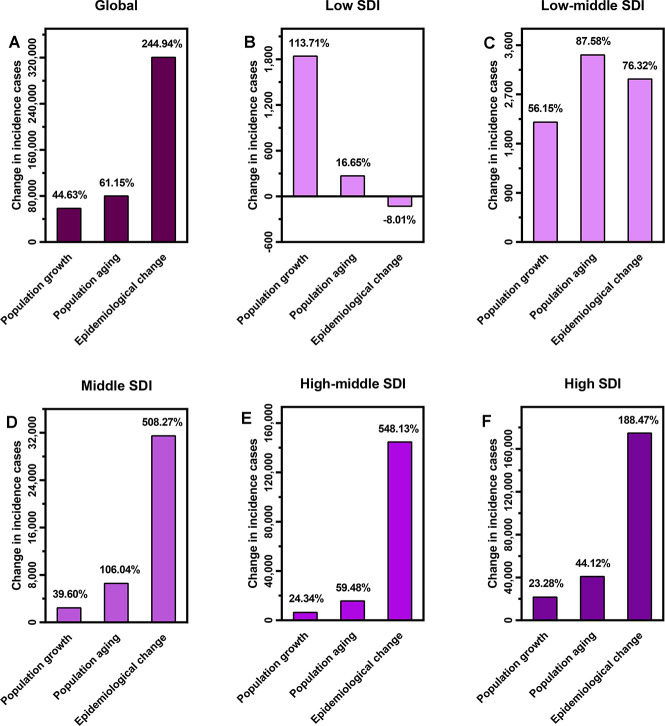
The proportions of CAVD incident cases varied from 1990 to 2019 attributed to population growth, population aging, and epidemiological change in (**A**) the globe; (**B**) low SDI quintiles; (**C**) low-middle SDI quintiles; (**D**) middle SDI quintiles; (**E**) high-middle SDI quintiles; and (**F**) high SDI quintiles. Epidemiological change refers to the CAVD incident cases variation when age structure and population remained constant. CAVD, calcific aortic valve disease; SDI: socio-demographic index.

Around the globe, the number of incident cases of CAVD increased from 130,822 in 1990 to 589,638 in 2019. According to decomposition analysis, population growth and population aging, and epidemiological changes accounted for 44.63%, 61.15%, and 244.94%, respectively. As for SDI regions, population growth accounted for 113.71%, 56.16%, 39.60%, 24.34%, and 23.28% of the changes of CAVD incident cases in low, low–middle, middle, high–middle, and high SDI quintiles, respectively. These proportions for population aging were 16.65%, 87.58%, 106.04%, 59.48%, and 44.12% in the five SDI quintiles, respectively. Furthermore, epidemiological changes accounted for −8.01%, −76.32%, 508.27%, 548.13%, and 188.47% in all SDI quintiles, respectively (Figure 4).

### Sex–age patterns of prevalence estimates

**Figure 5:**
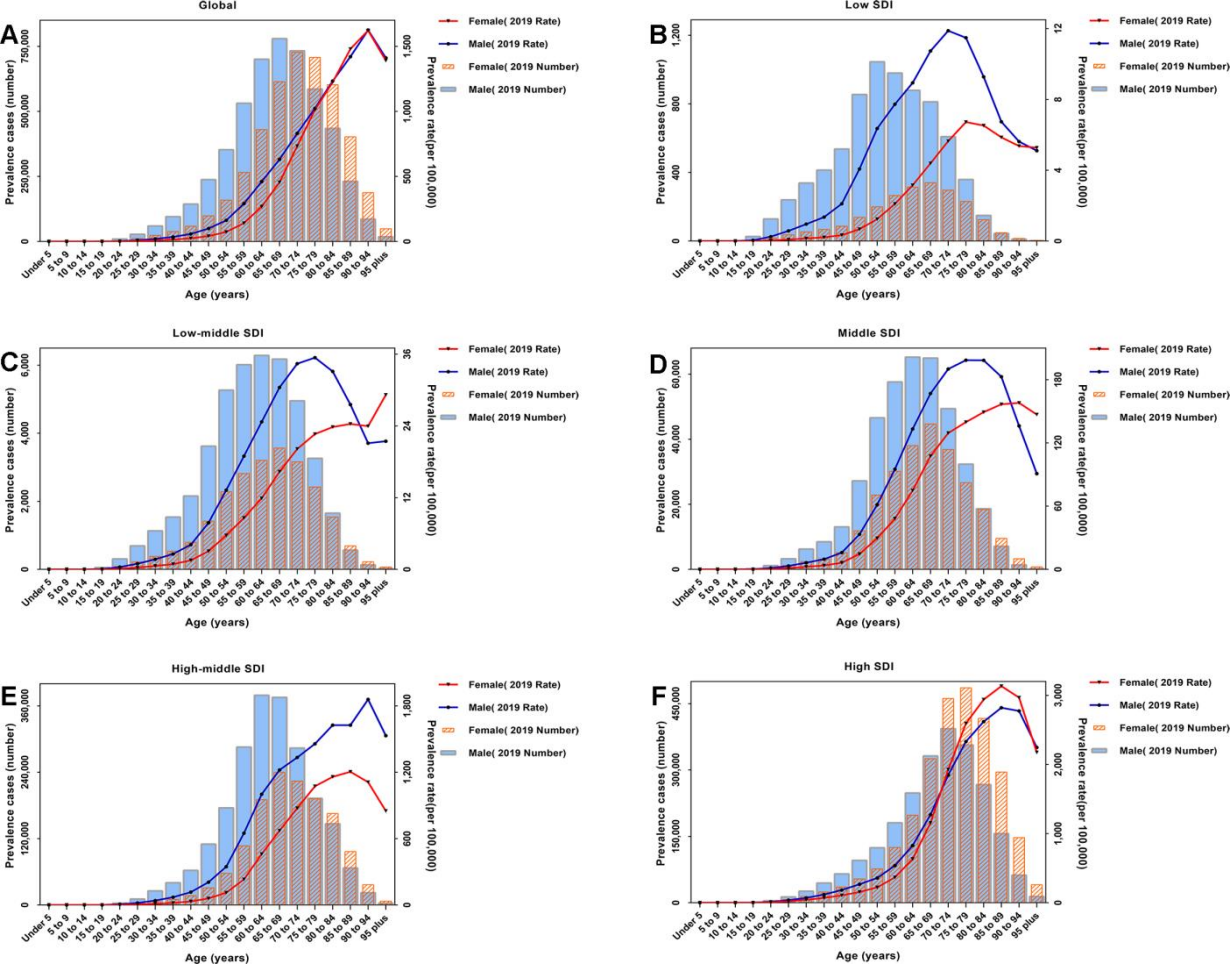
2019 absolute number of prevalent cases and prevalence rates per 100,000 persons of CAVD by sex and age in (**A**) the globe; (**B**) low SDI quintiles; (**C**) low-middle SDI quintiles; (**D**) middle SDI quintiles; (**E**) high-middle SDI quintiles; and (**F**) high SDI quintiles. Abbreviations: CAVD, calcific aortic valve disease; SDI: socio-demographic index.

Globally, the estimated prevalence of CAVD was higher in male individuals and increased with age, peaking in the age group 65–69 years for men and 70–74 years for women, followed by a downward trend. In 2019, the prevalence rate became much higher with age and approached the maximum among both men and women age 90–94 years. Among five SDI quintiles, patterns of prevalence estimates by sex varied dramatically across age groups. In low SDI quintiles, the number of prevalent cases reached the highest level among 50–54-year-old men and 65–69-year-old women; the prevalence rate peaked in men age 70–74 years and women age 75–79 years. As to high SDI quintiles, the prevalence rate reached the maximum in the age group 85–89 years for both sexes, whereas the number of prevalent cases peaked among 70–74-year-old men and 75–79-year-old women (Figure 5).

### Risk assessment for deaths

**Figure 6:**
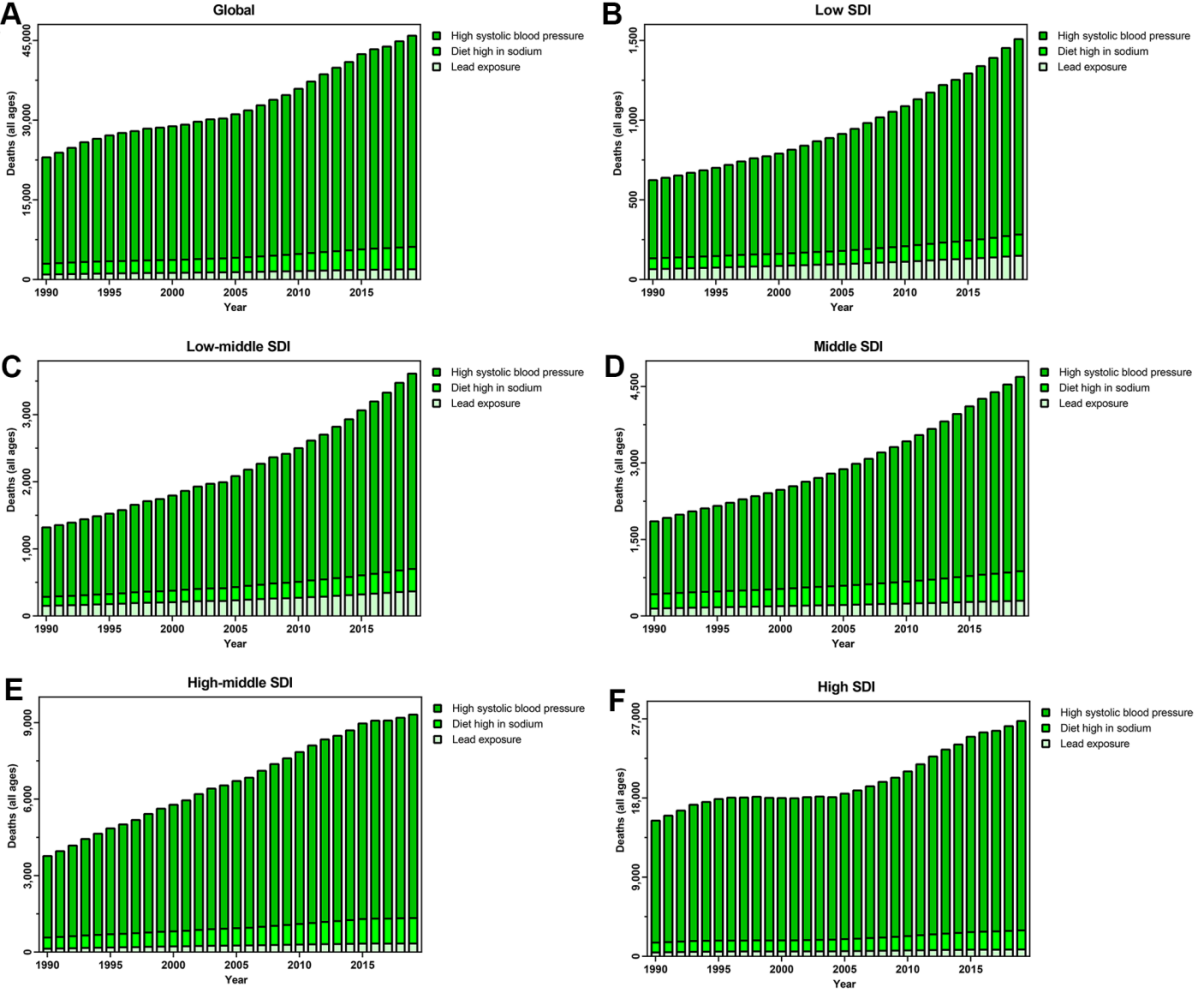
Risk factors contributing to CAVD-caused deaths in (**A**) the globe; (**B**) low SDI quintiles; (**C**) low-middle SDI quintiles; (**D**) middle SDI quintiles; (**E**) high-middle SDI quintiles; and (**F**) high SDI quintiles. Abbreviations: CAVD, calcific aortic valve disease; SDI: socio-demographic index.

On the basis of GBD research, CAVD deaths were mainly attributable to high SBP, diet high in sodium, and lead exposure. Worldwide, high SBP (the leading risk factor) led to 39,749 CAVD deaths in 2019. For SDI regions, deaths associated with this factor increased from 1,226 in low quintiles to 23,803 in high SDI quintiles. A high-sodium diet led to 4,227 global CAVD deaths in 2019, presenting an upward trend across all five SDI quintiles over the past three decades. Lead exposure resulted in 1,925 global CAVD deaths in 2019, an increase of 105.23% from 938 in 1990. Between 1990 and 2019, the change in deaths owing to lead exposure decreased from 148.88% in high-middle quintiles to 75.40% in high SDI quintiles (Figure 6).

### Correlation evaluation about EAPC

**Figure 7:**
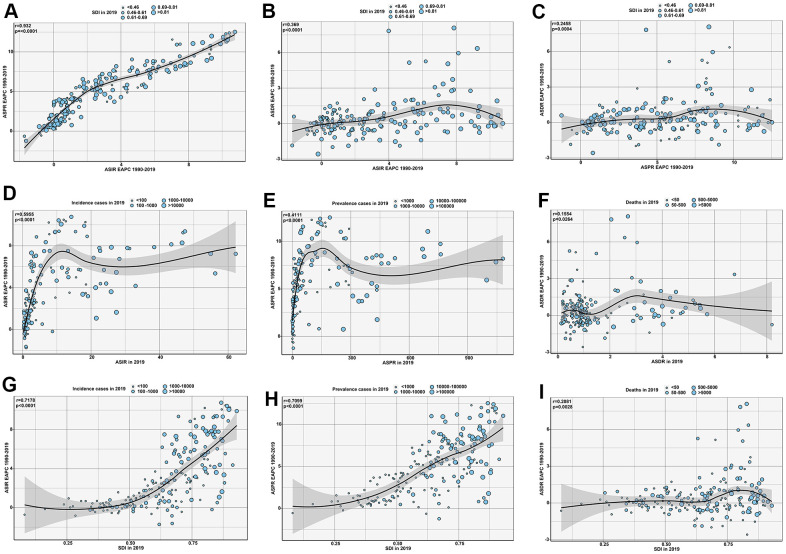
The correlation of CAVD between (**A**) EAPC of ASIR and EAPC of ASPR; (**B**) EAPC of ASIR and EAPC of ASDR; (**C**) EAPC of ASPR and EAPC of ASDR; (**D**) EAPC of ASIR and ASIR in 2019; (**E**) EAPC of ASPR and ASPR in 2019; (**F**) EAPC of ASDR and ASDR in 2019; (**G**) EAPC of ASIR and SDI in 2019; (**H**) EAPC of ASPR and SDI in 2019; and (**I**) EAPC of ASDR and ASDR in 2019. The circles represent countries that were available in the GBD 2019. The size of circle is increased with the SDI of countries in 2019 from (**A**) to (**C**), and the cases of CAVD in 2019 from (**D**) to (**I**). The r indices and p values presented were derived from Pearson correlation analysis. Abbreviations: CAVD, calcific aortic valve disease; EAPC, estimated annual percentage change; ASIR, age-standardized incidence rate; ASPR, age-standardized prevalence rate; ASDR, age-standardized deaths rate; SDI: socio-demographic index.; ASR, age standardized rate.

As shown in Figure 7, the EAPC in the ASIR showed an important correlation with the ASPR EAPC (r=0.932, p<0.0001) and ASDR EAPC (r=0.369, p<0.0001). Additionally, the ASIR EAPC was significantly related to the ASIR (r=0.5955, p<0.0001) and SDI (r=0.7178, p<0.0001) in 2019. Similarly, the ASPR EAPC was significantly associated with the ASPR (r=0.4111, p<0.0001) and SDI (r=0.7099, p<0.0001) in 2019 (Supplementary Figure 10). However, the ASDR EAPC had no significant correlation with the ASDR or SDI for all 204 countries in 2019.

## DISCUSSION

Our study used the most up-to-date information to demonstrate that (i) the spatial and temporal patterns of incidence, prevalence, and deaths owing to CAVD varied considerably across 204 nation-level units from 1990 to 2019, according to GBD 2019 findings; (ii) population aging contributed more to the CAVD incidence than population growth, according to decomposition analysis; (iii) the sex–age patterns of prevalence suggested that more men had CAVD and reached the highest prevalence at younger ages than women; (iv) CAVD deaths were mainly attributable to high SBP, diet high in sodium, and lead exposure, based on GBD 2019; and (v) the ASIR EAPC had an important correlation with the ASPR EAPC and ASDR EAPC whereas the ASIR and ASPR EAPC was significantly associated with ASR and SDI in 2019 throughout the 204 countries and territories.

Our results suggest that the global number of incident cases of CAVD have increased continuously, although the ASIR has remained relatively constant for the past three decades. Echocardiography, a noninvasive method to explore morphological and hemodynamic characteristics of the heart, can serve as an excellent source of information regarding valve anatomy and blood flow parameters in diagnosing CAVD [10, 11]. As this diagnostic tool has become more widely available, the reported incidence of CAVD has grown gradually. This may explain why higher SDI countries, such as Slovenia, Hungary, and Romania, had the highest ASIR. With more widespread screening, it is reasonable to speculate that the incidence of CAVD will continue to increase worldwide, especially in lower SDI regions. Additionally, compared with population growth, population aging may be a strong factor in the trends of CAVD incidence, according to our decomposition analysis. Despite disproval of the notion of CAVD is a degenerative disorder, owing to the advanced age of patients, age remains an important contributor to this disease. In a study by Tromsø, Cox proportional hazards regression was used, with age (hazard ratio 1.11, 95% CI: 1.08–1.14) identified as an independent predictor for incident CAVD [12]. Recently, a cohort study disclosed that aortic valve calcification density was related to age (β estimate±standard error: 6.5±1.8; p=0.0004) in a multivariate analysis [13]; the potential causes may include activation of the renin–angiotensin system and alteration of the phosphocalcic metabolism in older patients [14–16].

The CAVD prevalence increased globally from 1990 to 2019, not only in terms of number of cases but also ASR. This might partly reflect the increasing trend in incidence, according to the significant correlation between the ASIR EAPC and ASPR EAPC. With the exception of high SDI regions, the patterns by sex showed that more male than female individuals had CAVD. This finding is in keeping with earlier evidence. Median aortic valve calcification (2741 [1839–3858] AU vs. 1279 [882–1915] AU; p<0.0001) and mean valve weight (2.66±1.07 g vs. 1.87±0.58 g; p<0.0001) were higher in male than female patients in a study assessing sex differences in aortic valve fibro-calcific remodeling [17]. Similarly, in the Framingham Offspring Study, female patients had 56% lower odds (95% CI: 41%–76%; p=0.0003) of aortic valve calcium compared with their male counterparts in multivariable models adjusted for long-term risk factors [18]. Investigation of the intrinsic mechanisms of valves between male and female patients at cellular and genetic levels is suggested to clarify the difference in CAVD by sex. Relative to women, proinflammatory, proangiogenic, and procalcific actions of interferon gamma in valvular interstitial cells have been found to be more pronounced in men [19]. Furthermore, inhibition of vascular mineralization, upregulation of matrix Gla-protein, and higher expression of antiapoptotic gene *BCL2* were observed in aortic valve tissue from women [20]. Moreover, women manifest a lower expression level of pro-fibrotic TGF-β than men [21]. Additionally, the age patterns suggested that men reached the highest level of prevalence at younger ages than women, which was similar to previous findings. Male patients are reported to develop severe CAVD nearly 10 years earlier than female patients [22]. In an LDLr^−/−^/ApoB^100/100^/IGF-II CAVD mouse model, early initiation of CAVD in male mice is mediated by gonadal hormones to some degree, as validated through the attenuation effect of gonadectomy [23].

Although the prevalent cases and ASPR of CAVD showed alarming patterns worldwide, the trend in the global ASDR remained relatively steady throughout the study period, which might represent a balance between steadily improving therapeutic strategies and greater exposure to risk factors. In addition to increasingly perfected operative treatments, including the development and widely use of transcatheter aortic valve replacement, agents used to treat cardiovascular complications may work in CAVD [4, 24]. Tissue-engineered heart valves may exert a widespread and far-reaching impact on the field of heart valve prosthesis [25]. Moreover, the possibility that lipid-lowering therapy improves CAVD outcomes cannot be ruled out [26], albeit this specific drug strategy has failed to reduce disease progression. Anti-inflammatory medications also provide a promising pharmacological treatment, which warrants further research in patients with CAVD [27]. Denosumab, a human monoclonal antibody, has recently been reported to lower valvular calcium deposition [28].

In our study, three main risk factors have been identified. High SBP is the most well-known risk factor associated with CAVD. In the PROGRESSA study, compared with patients who did not have high SBP at baseline, those with high SBP had faster 2-year aortic valve calcification progression (median [25th percentile–75th percentile]: 157 [58–303] AU vs. 370 [126–824] AU; p=0.007, respectively); this association persisted in multivariable analysis [29]. The possibility that angiotensin II contributes to aortic valve fibrosis, remodeling, and calcification has been suggested in a large number of studies [30, 31]. Systolic hypertension may result from the activated renin–angiotensin–aldosterone system. Moreover, high SBP may increase mechanical stress on the valvular structure during systole, which leads to endothelial damage, further promoting the inflammatory response in the valve [3, 32]. Additionally, systolic hypertension may cause the activation of valve interstitial cells to develop a secretory phenotype by increasing the mechanical strain on these cells [33]. A diet high in sodium, one of the top 10 risk factors for disability [34], is another significant contributor to CAVD deaths. There is increasing evidence that a high-sodium diet enhances the risk of cardiovascular and cerebrovascular events, which is associated with the effect of aldosterone on renal mineralocorticoid receptors [35]. In a hyperlipidemic rabbit model, aldosterone receptors were demonstrated to be present in aortic valve tissue, and their selective blockade could suppress processes in the early stage of CAVD [36]. Furthermore, a number of studies have suggested that a high sodium intake contributes to the occurrence of high blood pressure [37–39], which is another possible interpretation for the relationship between CAVD and diet high in sodium. Lead exposure was established as a potential risk factor related to CAVD deaths, which may be explained by evidence that chronic exposure to lead is a factor contributing to atherosclerosis and hypertension [40–42].

In the present study, we also carried out a series of correlation analyses and found that the ASIR EAPC showed an important correlation with the ASPR EAPC and ASDR EAPC. The burden and outcome of all diseases, including CAVD, originate from the occurrence of disease. We found that the temporal trend in the ASR of incidence and prevalence—that is, the EAPC—from 1990 to 2019 was positively correlated with the ASR in 2019, which is not difficult to explain. The ASR of CAVD reflects the disease burden at that time, which is closely related to the level and effectiveness of strategies to prevent CAVD. Additionally, a significant correlation was found between the EAPC and SDI in 2019, not only for incidence but also for prevalence. Possible explanations for this observation include the following: (i) people in countries with lower SDI are more likely to be exposed to communicable, maternal, neonatal, and nutritional diseases; and (ii) the higher the SDI, the better the screening and treatment measures.

Ongoing objectives of the GBD include improving the level of detail in estimations, improving analytical strategies, and increasing the amount of high-quality data. Nevertheless, some limitations should be pointed out in the current study. First, the accuracy and reliability of the GBD study hinge on the quantity and quality of data, which played a significant role in our analysis. For example, the diagnosis of CAVD depends on the availability of echocardiography. This may lead to underestimation concerning the real burden of CAVD, especially in many low-income countries or less-developed areas. Second, the temporal trends in disease burden according to CAVD subtype, such as bicuspid aortic valve and tricuspid aortic valve, were not estimated in this study owing to a lack of related raw data. Thirdly, we did not check the association of ASR and SDI according to CAVD severity, due to the lack of available information. Finally, calcified aortic valve stenosis and regurgitation were not differentiated.

## CONCLUSIONS

Globally, cases of CAVD have increased continuously over the past 30 years, with no downward trends observed in the ASIR, ASPR, or ASDR. Population aging was an important contributor to incident cases. Moreover, differences were present in the sex and age distributions of prevalent cases. A high SBP, a diet high in sodium, and exposure to lead were the main risk factors of CAVD deaths. Moreover, the EAPC was significantly related to the ASR and SDI in 2019 for incidence and prevalence. Generally, the findings of the present study will be helpful in elucidating the global disease burden of CAVD, to develop more effective and efficient CAVD prevention and treatment strategies.

## MATERIALS AND METHODS

### Data sources

The GBD study, a cooperative effort among researchers from institutions around the world, provides a unique opportunity to evaluate many different diseases, injures, and risk factors according to location, age, and sex. Global health has gradually improved over the past 30 years. Relative to the GBD 2017, the GBD 2019 has expanded analysis of the burden of disease in all World Health Organization member states, with nine countries and territories added to the GBD location hierarchy [9]. Additionally, 12 new causes of disease were added to the GBD modeling framework, including nine new cancer sites, pulmonary hypertension, and osteoarthritis of the hands and other joints [8]. Moreover, a total of 87 risk factors were updated in the GBD 2019, in comparison with 84 risk factors in the GBD 2017 [7].

To estimate deaths owing to CAVD, the GBD 2019 used the Cause of Death Ensemble model (CODEm), a highly systematic tool to analyze cause-of-death data using an ensemble of linear and mixed effects regression to minimize model-specification bias [43]. CAVD incidence and prevalence were estimated using large-scale population-representative data sources identified in document retrieval and via study collaborations. Consistent disease estimates were produced using Disease Modeling–Meta Regression (DisMod-MR) 2.1, a Bayesian meta-regression software tool used to simultaneously model the population data. The overall prevalence was estimated according to the corresponding health states listed as follows: patients with hemodynamically moderate disease, patients with hemodynamically severe disease who were treated, and patients with untreated hemodynamically severe disease and one of the following four outcomes: (i) controlled, medically managed heart failure; (ii) mild heart failure; (iii) moderate heart failure; and (iv) severe heart failure [10, 44].

The sociodemographic index (SDI), a metric for measuring development, which actually captures three different but important aspects of each country and region—income, education, and fertility—allows for the comparison of health outcomes among countries and the performance of health systems, to better understand what the health landscape would look like in the future [45]. According to SDI value, countries are divided into five quintiles: high (>0.81), high–middle (0.69–0.81), middle (0.61–0.69), low–middle (0.46–0.61), and low SDI (<0.46).

### Data identification and extraction

CAVD was identified using codes of the International Statistical Classification of Diseases and Related Health Problems, Ninth Revision and Tenth Revision (ICD-9 and ICD-10, respectively). All cardiovascular diseases coded as 424.1 in the ICD-9 and I35, I35.0, I35.1, I35.2, I35.8, and I35.9 in the ICD-10 were mapped to CAVD in the study [44]. Consistent with previous GBD studies, the diagnosis of CAVD required an echocardiography demonstrating calcification of the bicuspid aortic valve [10]. The following data on CAVD were extracted from the GBD 2019 (http://ghdx.healthdata.org/gbd-results-tool): global, regional, and national population, incidence, prevalence and deaths attributable to CAVD by sex and age, as well as national SDI value from 1990 to 2019. All measures were expressed as initial data, rate per 100,000 persons, and age-standardized rate (ASR) per 100,000 persons, including age-standardized incidence rate (ASIR), age-standardized prevalence rate (ASPR) and age-standardized death rate (ASDR). Age standardization was according to the World Health Organization, using an average world population age structure [46].

### Statistical analysis and synthesis

For all ages, the fold change in the number and amplitude of variation in ASR, namely, the estimated annual percentage change (EAPC), was used to evaluate secular trends in CAVD. The EAPC, a concise, condensed measure of ASR variation during a specified interval, can represent a shift in the burden of disease among a certain demographic group and may serve as a key to changing risk factors. The EAPC was identified to be 100×(exp(β)−1) and the corresponding 95% confidence interval (CI) was calculated using a linear regression model: y=α+βx+ε, where y=ln(ASR) and x=calendar year [47]. Briefly, the ASR showed a decreasing trend if the EAPC estimate and the upper boundary of its 95% CI were both <0. In contrast, the ASR indicated an increasing trend if the EAPC estimate and lower boundary of its 95% CI were both >0. Otherwise, the ASR was regarded as stable over time.

We performed a decomposition analysis to investigate the influences of population growth, population aging, and changes in epidemiology on the variation in CAVD incidence between 1990 and 2019, as follows: by (i) calculating incident cases predicted in a scenario with the age structure in 2019 and without population growth since 1990; and (ii) calculating the incident cases predicted in another scenario with the age structure in 1990 and presuming the same population growth to 2019 [48].

Moreover, to explore the distribution of sex and age regarding CAVD prevalence, we obtained the crude number and rates of prevalence, to analyze the sex–age patterns for male and female individuals in 2019, according to the 20 age groups listed below: under 5, 5–9, 10–14, 15–19, 20–24, 25–29, 30–34, 35–39, 40–44, 45–49, 50–54, 55–59, 60–64, 65–69, 70–74, 75–79, 80–84, 85–89, 90–94, and 95 years and over.

Three primary risk factors of death owing to CAVD were identified in the GBD 2019, including high systolic blood pressure (SBP), diet high in sodium, and lead exposure. To assess the influences of high SBP, high-sodium diet, and lead exposure, we applied the theoretical minimum level, with 110–115 mmHg for SBP, 1–5 g per day for 24-h urinary sodium, and 2 μg/dL in the blood, corresponding to lead levels in pre-industrial humans owing to natural sources of lead, to prevent the possible exposure of zero [49].

Pearson correlation tests were used to assess correlations among the EAPC in the ASIR, ASPR, and ASDR of all 204 countries and territories. Additionally, to investigate possible factors affecting the EAPC, its correlation with the SDI and ASR was analyzed at national levels in 2019. Statistically significant correlation was considered with p<0.05 and r>0.3. All statistical analyses and visualizations were conducted using GraphPad Prism 7 (GraphPad Software, San Diego, CA, USA) and R version 3.5.2 (The R Project for Statistical Computing, Vienna, Austria).

## Supplementary Material

Supplementary Figures

Supplementary Table 1

Supplementary Table 2

Supplementary Table 3

Supplementary Table 4

## References

[1] Kostyunin AE, Yuzhalin AE, Ovcharenko EA, Kutikhin AG. Development of calcific aortic valve disease: Do we know enough for new clinical trials? J Mol Cell Cardiol. 2019; 132:189–209. 10.1016/j.yjmcc.2019.05.016 31136747

[2] Pasipoularides A. Calcific Aortic Valve Disease: Part 1--Molecular Pathogenetic Aspects, Hemodynamics, and Adaptive Feedbacks. J Cardiovasc Transl Res. 2016; 9:102–18. 10.1007/s12265-016-9679-z 26891845 PMC4833551

[3] Rajamannan NM, Evans FJ, Aikawa E, Grande-Allen KJ, Demer LL, Heistad DD, Simmons CA, Masters KS, Mathieu P, O’Brien KD, Schoen FJ, Towler DA, Yoganathan AP, Otto CM. Calcific aortic valve disease: not simply a degenerative process: A review and agenda for research from the National Heart and Lung and Blood Institute Aortic Stenosis Working Group. Executive summary: Calcific aortic valve disease-2011 update. Circulation. 2011; 124:1783–91. 10.1161/CIRCULATIONAHA.110.006767 22007101 PMC3306614

[4] Alushi B, Curini L, Christopher MR, Grubitzch H, Landmesser U, Amedei A, Lauten A. Calcific Aortic Valve Disease-Natural History and Future Therapeutic Strategies. Front Pharmacol. 2020; 11:685. 10.3389/fphar.2020.00685 32477143 PMC7237871

[5] Zilla P, Yacoub M, Zühlke L, Beyersdorf F, Sliwa K, Khubulava G, Bouzid A, Mocumbi AO, Velayoudam D, Shetty D, Ofoegbu C, Geldenhuys A, Brink J, et al. Global Unmet Needs in Cardiac Surgery. Glob Heart. 2018; 13:293–303. 10.1016/j.gheart.2018.08.002 30245177

[6] Nishimura RA, Otto CM, Bonow RO, Carabello BA, Erwin JP 3rd, Guyton RA, O’Gara PT, Ruiz CE, Skubas NJ, Sorajja P, Sundt TM 3rd, Thomas JD, and American College of Cardiology/American Heart Association Task Force on Practice Guidelines. 2014 AHA/ACC guideline for the management of patients with valvular heart disease: executive summary: a report of the American College of Cardiology/American Heart Association Task Force on Practice Guidelines. J Am Coll Cardiol. 2014; 63:2438–88. 10.1016/j.jacc.2014.02.537 24603192

[7] GBD 2019 Risk Factors Collaborators. Global burden of 87 risk factors in 204 countries and territories, 1990-2019: a systematic analysis for the Global Burden of Disease Study 2019. Lancet. 2020; 396:1223–49. 10.1016/S0140-6736(20)30752-2 33069327 PMC7566194

[8] GBD 2019 Diseases and Injuries Collaborators. Global burden of 369 diseases and injuries in 204 countries and territories, 1990-2019: a systematic analysis for the Global Burden of Disease Study 2019. Lancet. 2020; 396:1204–22. 10.1016/S0140-6736(20)30925-9 33069326 PMC7567026

[9] GBD 2019 Demographics Collaborators. Global age-sex-specific fertility, mortality, healthy life expectancy (HALE), and population estimates in 204 countries and territories, 1950-2019: a comprehensive demographic analysis for the Global Burden of Disease Study 2019. Lancet. 2020; 396:1160–203. 10.1016/S0140-6736(20)30977-6 33069325 PMC7566045

[10] Yadgir S, Johnson CO, Aboyans V, Adebayo OM, Adedoyin RA, Afarideh M, Alahdab F, Alashi A, Alipour V, Arabloo J, Azari S, Barthelemy CM, Benziger CP, et al, and Global Burden of Disease Study 2017 Nonrheumatic Valve Disease Collaborators. Global, Regional, and National Burden of Calcific Aortic Valve and Degenerative Mitral Valve Diseases, 1990-2017. Circulation. 2020; 141:1670–80. 10.1161/CIRCULATIONAHA.119.043391 32223336

[11] Baumgartner H, Hung J, Bermejo J, Chambers JB, Edvardsen T, Goldstein S, Lancellotti P, LeFevre M, Miller F Jr, Otto CM. Recommendations on the echocardiographic assessment of aortic valve stenosis: a focused update from the European Association of Cardiovascular Imaging and the American Society of Echocardiography. Eur Heart J Cardiovasc Imaging. 2017; 18:254–75. 10.1093/ehjci/jew335 28363204

[12] Eveborn GW, Schirmer H, Lunde P, Heggelund G, Hansen JB, Rasmussen K. Assessment of risk factors for developing incident aortic stenosis: the Tromsø Study. Eur J Epidemiol. 2014; 29:567–75. 10.1007/s10654-014-9936-x 25023627

[13] Voisine M, Hervault M, Shen M, Boilard AJ, Filion B, Rosa M, Bossé Y, Mathieu P, Côté N, Clavel MA. Age, Sex, and Valve Phenotype Differences in Fibro-Calcific Remodeling of Calcified Aortic Valve. J Am Heart Assoc. 2020; 9:e015610. 10.1161/JAHA.119.015610 32384012 PMC7660864

[14] Côté N, Mahmut A, Fournier D, Boulanger MC, Couture C, Després JP, Trahan S, Bossé Y, Pagé S, Pibarot P, Mathieu P. Angiotensin receptor blockers are associated with reduced fibrosis and interleukin-6 expression in calcific aortic valve disease. Pathobiology. 2014; 81:15–24. 10.1159/000350896 23969418

[15] Capoulade R, Clavel MA, Dumesnil JG, Chan KL, Teo KK, Tam JW, Côté N, Mathieu P, Després JP, Pibarot P, and ASTRONOMER Investigators. Impact of metabolic syndrome on progression of aortic stenosis: influence of age and statin therapy. J Am Coll Cardiol. 2012; 60:216–23. 10.1016/j.jacc.2012.03.052 22789885

[16] Mohty D, Pibarot P, Després JP, Cartier A, Arsenault B, Picard F, Mathieu P. Age-related differences in the pathogenesis of calcific aortic stenosis: the potential role of resistin. Int J Cardiol. 2010; 142:126–32. 10.1016/j.ijcard.2008.12.068 19162347

[17] Simard L, Côté N, Dagenais F, Mathieu P, Couture C, Trahan S, Bossé Y, Mohammadi S, Pagé S, Joubert P, Clavel MA. Sex-Related Discordance Between Aortic Valve Calcification and Hemodynamic Severity of Aortic Stenosis: Is Valvular Fibrosis the Explanation? Circ Res. 2017; 120:681–91. 10.1161/CIRCRESAHA.116.309306 27879282

[18] Thanassoulis G, Massaro JM, Cury R, Manders E, Benjamin EJ, Vasan RS, Cupple LA, Hoffmann U, O’Donnell CJ, Kathiresan S. Associations of long-term and early adult atherosclerosis risk factors with aortic and mitral valve calcium. J Am Coll Cardiol. 2010; 55:2491–98. 10.1016/j.jacc.2010.03.019 20510217 PMC3042249

[19] Parra-Izquierdo I, Castaños-Mollor I, López J, Gómez C, San Román JA, Sánchez Crespo M, García-Rodríguez C. Lipopolysaccharide and interferon-γ team up to activate HIF-1α via STAT1 in normoxia and exhibit sex differences in human aortic valve interstitial cells. Biochim Biophys Acta Mol Basis Dis. 2019; 1865:2168–79. 10.1016/j.bbadis.2019.04.014 31034990

[20] Parra-Izquierdo I, Castaños-Mollor I, López J, Gómez C, San Román JA, Sánchez Crespo M, García-Rodríguez C. Calcification Induced by Type I Interferon in Human Aortic Valve Interstitial Cells Is Larger in Males and Blunted by a Janus Kinase Inhibitor. Arterioscler Thromb Vasc Biol. 2018; 38:2148–59. 10.1161/ATVBAHA.118.311504 30026273

[21] Kararigas G, Dworatzek E, Petrov G, Summer H, Schulze TM, Baczko I, Knosalla C, Golz S, Hetzer R, Regitz-Zagrosek V. Sex-dependent regulation of fibrosis and inflammation in human left ventricular remodelling under pressure overload. Eur J Heart Fail. 2014; 16:1160–67. 10.1002/ejhf.171 25287281

[22] Fuchs C, Mascherbauer J, Rosenhek R, Pernicka E, Klaar U, Scholten C, Heger M, Wollenek G, Czerny M, Maurer G, Baumgartner H. Gender differences in clinical presentation and surgical outcome of aortic stenosis. Heart. 2010; 96:539–45. 10.1136/hrt.2009.186650 20350991

[23] Annabi MS, Clisson M, Fleury MA, Voisine M, Hervault M, Shen M, Boilard AJ, Marette A, Ong G, Côté N, Clavel MA. Sex-differences in echocardiographic assessment of aortic valve in young adult LDLr^-/-^/ApoB^100/100^/IGF-II^+/-^ mice. Exp Gerontol. 2020; 140:111075. 10.1016/j.exger.2020.111075 32861845

[24] Prendergast BD, Redwood SR. Transcatheter Aortic Valve Replacement. Circulation. 2019; 139:2724–27. 10.1161/CIRCULATIONAHA.119.040016 31180746

[25] Durko AP, Yacoub MH, Kluin J. Tissue Engineered Materials in Cardiovascular Surgery: The Surgeon’s Perspective. Front Cardiovasc Med. 2020; 7:55. 10.3389/fcvm.2020.00055 32351975 PMC7174659

[26] Rossebø AB, Pedersen TR, Boman K, Brudi P, Chambers JB, Egstrup K, Gerdts E, Gohlke-Bärwolf C, Holme I, Kesäniemi YA, Malbecq W, Nienaber CA, Ray S, et al, and SEAS Investigators. Intensive lipid lowering with simvastatin and ezetimibe in aortic stenosis. N Engl J Med. 2008; 359:1343–56. 10.1056/NEJMoa0804602 18765433

[27] Hurle MR, Yang L, Xie Q, Rajpal DK, Sanseau P, Agarwal P. Computational drug repositioning: from data to therapeutics. Clin Pharmacol Ther. 2013; 93:335–41. 10.1038/clpt.2013.1 23443757

[28] Lerman DA, Prasad S, Alotti N. Calcific Aortic Valve Disease: Molecular Mechanisms and Therapeutic Approaches. Eur Cardiol. 2015; 10:108–12. 10.15420/ecr.2015.10.2.108 27274771 PMC4888946

[29] Tastet L, Capoulade R, Clavel MA, Larose É, Shen M, Dahou A, Arsenault M, Mathieu P, Bédard É, Dumesnil JG, Tremblay A, Bossé Y, Després JP, Pibarot P. Systolic hypertension and progression of aortic valve calcification in patients with aortic stenosis: results from the PROGRESSA study. Eur Heart J Cardiovasc Imaging. 2017; 18:70–78. 10.1093/ehjci/jew013 26896413 PMC5217738

[30] Fujisaka T, Hoshiga M, Hotchi J, Takeda Y, Jin D, Takai S, Hanafusa T, Ishizaka N. Angiotensin II promotes aortic valve thickening independent of elevated blood pressure in apolipoprotein-E deficient mice. Atherosclerosis. 2013; 226:82–87. 10.1016/j.atherosclerosis.2012.10.055 23177972

[31] Akat K, Borggrefe M, Kaden JJ. Aortic valve calcification: basic science to clinical practice. Heart. 2009; 95:616–23. 10.1136/hrt.2007.134783 18632833

[32] Arjunon S, Rathan S, Jo H, Yoganathan AP. Aortic valve: mechanical environment and mechanobiology. Ann Biomed Eng. 2013; 41:1331–46. 10.1007/s10439-013-0785-7 23515935 PMC5460154

[33] Balachandran K, Sucosky P, Yoganathan AP. Hemodynamics and mechanobiology of aortic valve inflammation and calcification. Int J Inflam. 2011; 2011:263870. 10.4061/2011/263870 21760982 PMC3133012

[34] Paczula A, Wiecek A, Piecha G. Cardiotonic Steroids-A Possible Link Between High-Salt Diet and Organ Damage. Int J Mol Sci. 2019; 20:590. 10.3390/ijms20030590 30704040 PMC6386955

[35] Conlin PR. Interactions of high salt intake and the response of the cardiovascular system to aldosterone. Cardiol Rev. 2005; 13:118–24. 10.1097/01.crd.0000148845.74124.c0 15831143

[36] Gkizas S, Koumoundourou D, Sirinian X, Rokidi S, Mavrilas D, Koutsoukos P, Papalois A, Apostolakis E, Alexopoulos D, Papadaki H. Aldosterone receptor blockade inhibits degenerative processes in the early stage of calcific aortic stenosis. Eur J Pharmacol. 2010; 642:107–12. 10.1016/j.ejphar.2010.05.048 20553922

[37] Komnenov D, Levanovich PE, Rossi NF. Hypertension Associated with Fructose and High Salt: Renal and Sympathetic Mechanisms. Nutrients. 2019; 11:569. 10.3390/nu11030569 30866441 PMC6472002

[38] Titze J, Luft FC. Speculations on salt and the genesis of arterial hypertension. Kidney Int. 2017; 91:1324–35. 10.1016/j.kint.2017.02.034 28501304

[39] Pilic L, Pedlar CR, Mavrommatis Y. Salt-sensitive hypertension: mechanisms and effects of dietary and other lifestyle factors. Nutr Rev. 2016; 74:645–58. 10.1093/nutrit/nuw028 27566757

[40] Vaziri ND. Mechanisms of lead-induced hypertension and cardiovascular disease. Am J Physiol Heart Circ Physiol. 2008; 295:H454–65. 10.1152/ajpheart.00158.2008 18567711 PMC2519216

[41] Harari F, Barregard L, Östling G, Sallsten G, Hedblad B, Forsgard N, Borné Y, Fagerberg B, Engström G. Blood Lead Levels and Risk of Atherosclerosis in the Carotid Artery: Results from a Swedish Cohort. Environ Health Perspect. 2019; 127:127002. 10.1289/EHP5057 31808705 PMC6957277

[42] Cheung B, Tsoi MF, Lui K, Cheung TT. Blood Lead Level and Hypertension Risk in the United States National Health Nutrition and Examination Survey (NHANES) 1999-2016. Eur Cardiol. 2020; 15:e36. 10.15420/ecr.2020.15.1.PO13 32612696 PMC7312713

[43] Foreman KJ, Lozano R, Lopez AD, Murray CJ. Modeling causes of death: an integrated approach using CODEm. Popul Health Metr. 2012; 10:1. 10.1186/1478-7954-10-1 22226226 PMC3315398

[44] Roth GA, Mensah GA, Johnson CO, Addolorato G, Ammirati E, Baddour LM, Barengo NC, Beaton AZ, Benjamin EJ, Benziger CP, Bonny A, Brauer M, Brodmann M, et al, and GBD-NHLBI-JACC Global Burden of Cardiovascular Diseases Writing Group. Global Burden of Cardiovascular Diseases and Risk Factors, 1990-2019: Update From the GBD 2019 Study. J Am Coll Cardiol. 2020; 76:2982–3021. 10.1016/j.jacc.2020.11.010 33309175 PMC7755038

[45] Haagsma JA, James SL, Castle CD, Dingels ZV, Fox JT, Hamilton EB, Liu Z, Lucchesi LR, Roberts NL, Sylte DO, Adebayo OM, Ahmadi A, Ahmed MB, et al. Burden of injury along the development spectrum: associations between the Socio-demographic Index and disability-adjusted life year estimates from the Global Burden of Disease Study 2017. Inj Prev. 2020; 26:i12–26. 10.1136/injuryprev-2019-043296 31915273 PMC7571356

[46] Ahmad O, Boschi-Pinto C, Lopez A, Murray C, Lozano R, Inoue M. Age standardization of rates: a new WHO standard. GPE Discussion Paper Series: No.31 EIP/GPE/EBD. Geneva: World Health Organization, 2001. https://www.who.int/healthinfo/paper31.pdf

[47] Yi M, Zhou L, Li A, Luo S, Wu K. Global burden and trend of acute lymphoblastic leukemia from 1990 to 2017. Aging (Albany NY). 2020; 12:22869–91. 10.18632/aging.103982 33203796 PMC7746341

[48] Li H, Lu W, Wang A, Jiang H, Lyu J. Changing epidemiology of chronic kidney disease as a result of type 2 diabetes mellitus from 1990 to 2017: Estimates from Global Burden of Disease 2017. J Diabetes Investig. 2021; 12:346–56. 10.1111/jdi.13355 32654341 PMC7926234

[49] GBD 2017 Risk Factor Collaborators. Global, regional, and national comparative risk assessment of 84 behavioural, environmental and occupational, and metabolic risks or clusters of risks for 195 countries and territories, 1990-2017: a systematic analysis for the Global Burden of Disease Study 2017. Lancet. 2018; 392:1923–94. 10.1016/S0140-6736(18)32225-6 30496105 PMC6227755

