# Supplementary material for: Changing epidemiology of calcific aortic valve disease: 30-year trends of incidence, prevalence, and deaths across 204 countries and territories
Source: Aging (Albany NY). 2021 May 11;13(9):12710–32. doi: 10.18632/aging.202942 (PMC8148466; doi:10.18632/aging.202942)
Supplement: Supplementary Figures [file aging-13-202942-s001.pdf]

SUPPLEMENTARY FIGURES

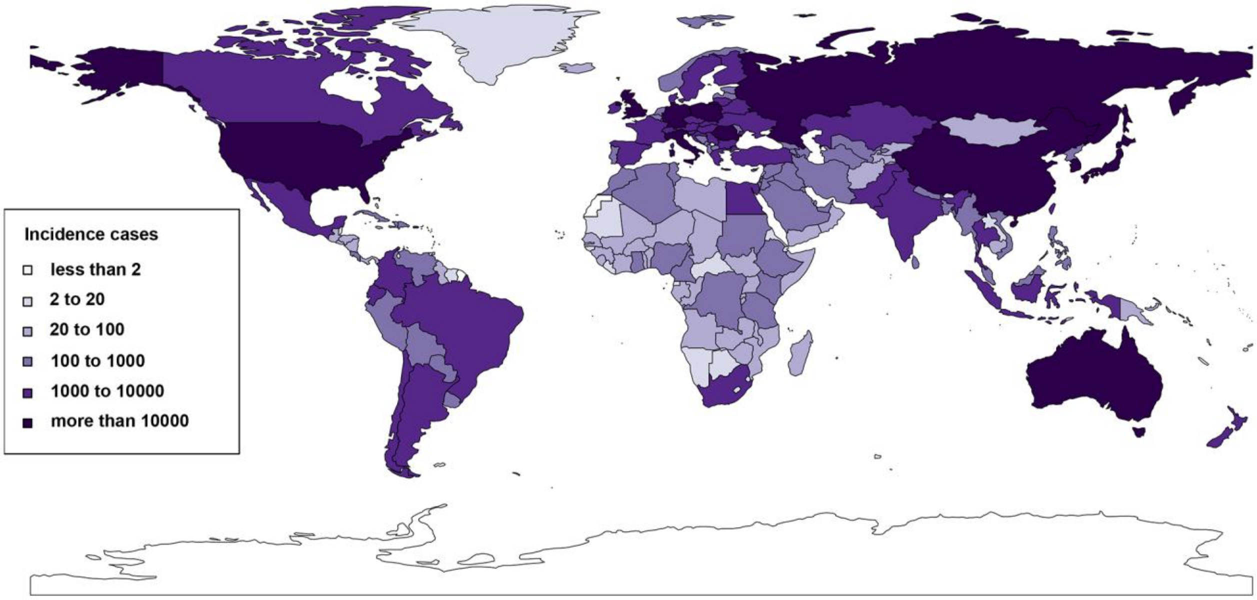

Supplementary Figure 1. The all-ages incidence cases of calcific aortic valve disease across 204 countries and territories in 2019.

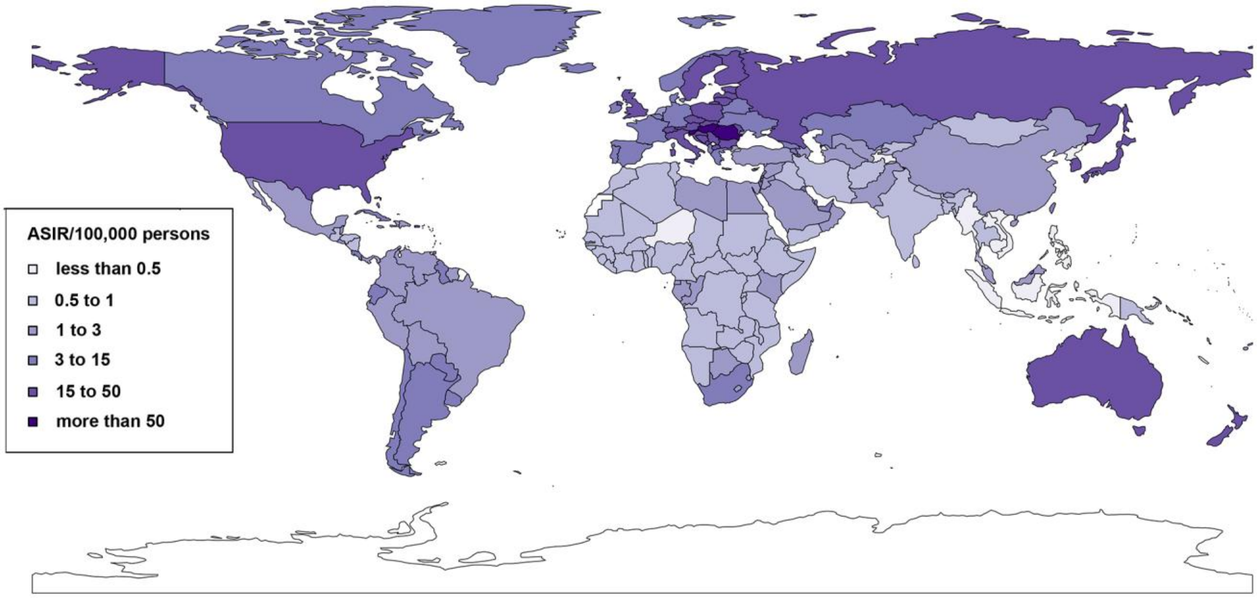

Supplementary Figure 2. The age standardized incidence rate of calcific aortic valve disease across 204 countries and territories in 2019.

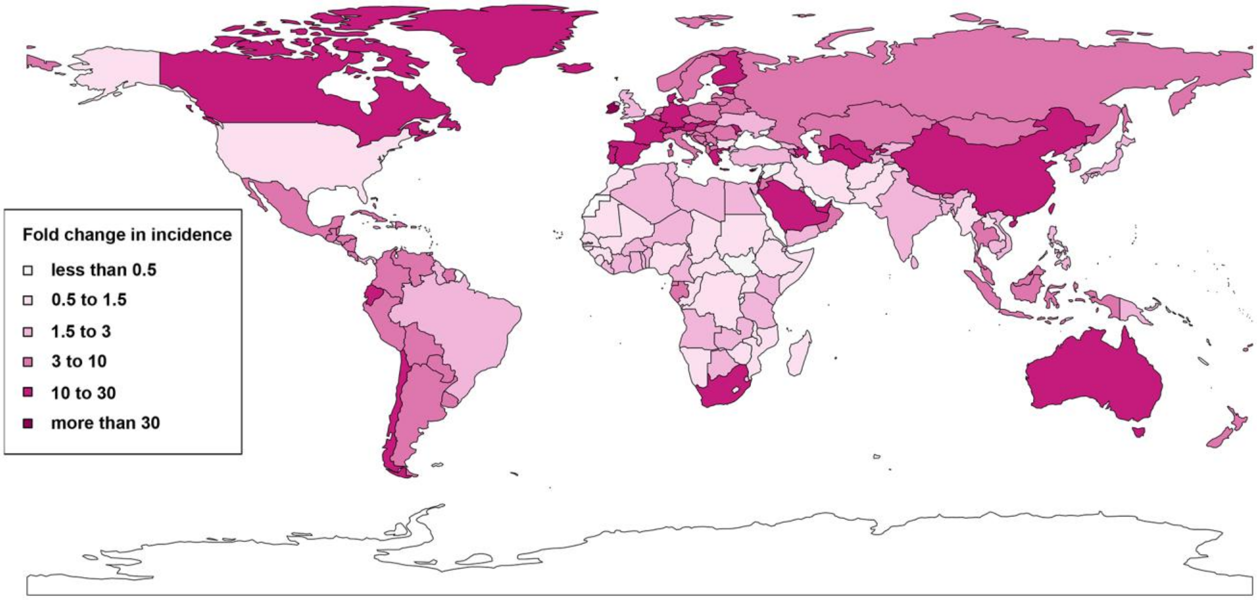

Supplementary Figure 3. The fold change in all-ages incidence cases of calcific aortic valve disease across 204 countries and territories between 1990 and 2019.

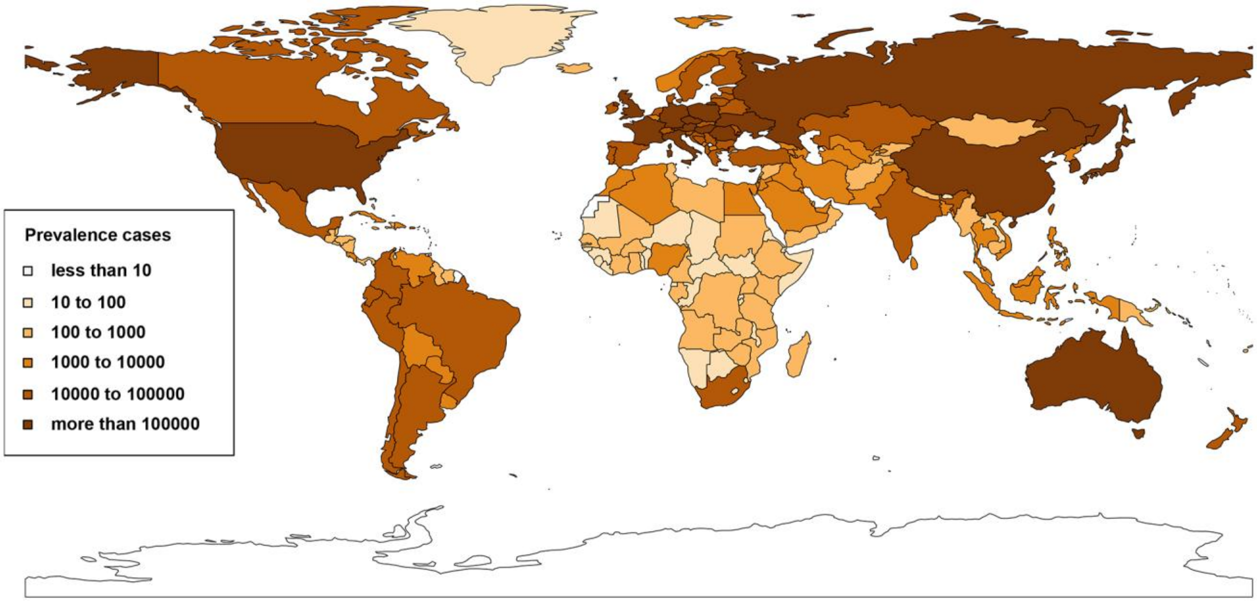

Supplementary Figure 4. The all-ages prevalence cases of calcific aortic valve disease across 204 countries and territories in 2019.

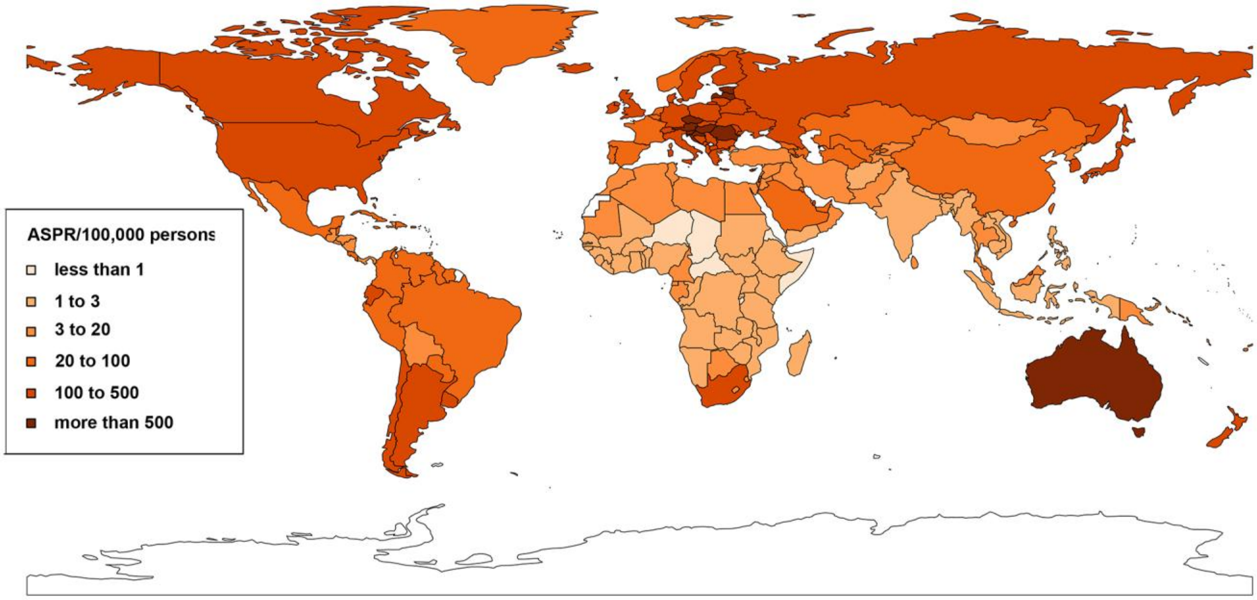

Supplementary Figure 5. The age standardized prevalence rate of calcific aortic valve disease across 204 countries and territories in 2019.

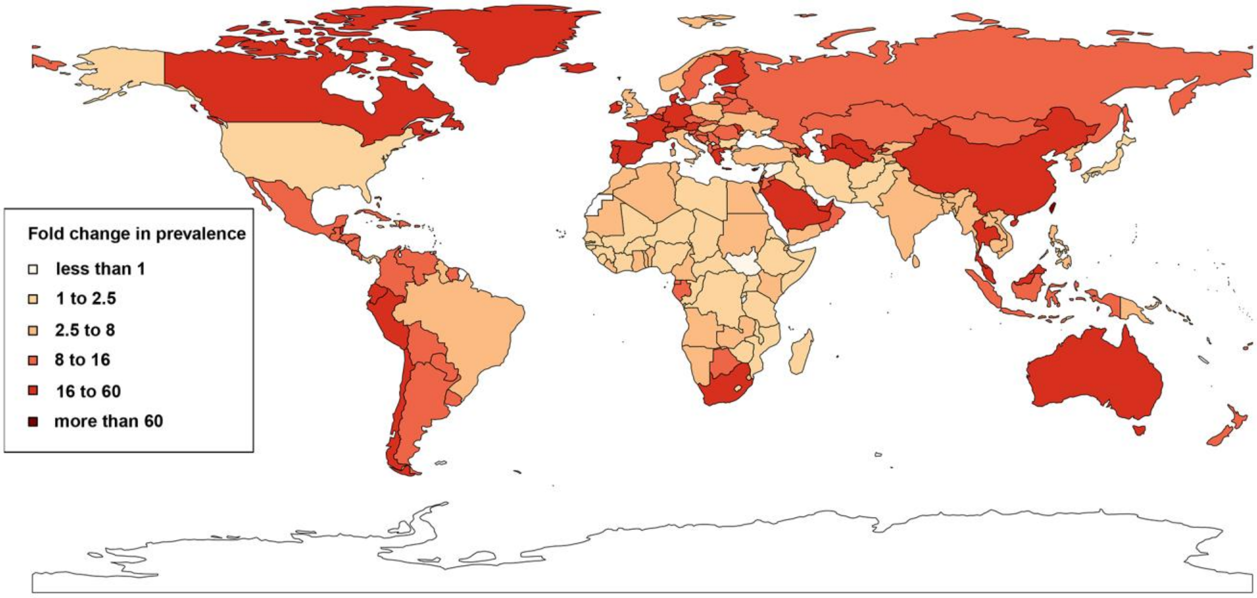

Supplementary Figure 6. The fold change in all-ages prevalence cases of calcific aortic valve disease across 204 countries and territories between 1990 and 2019.

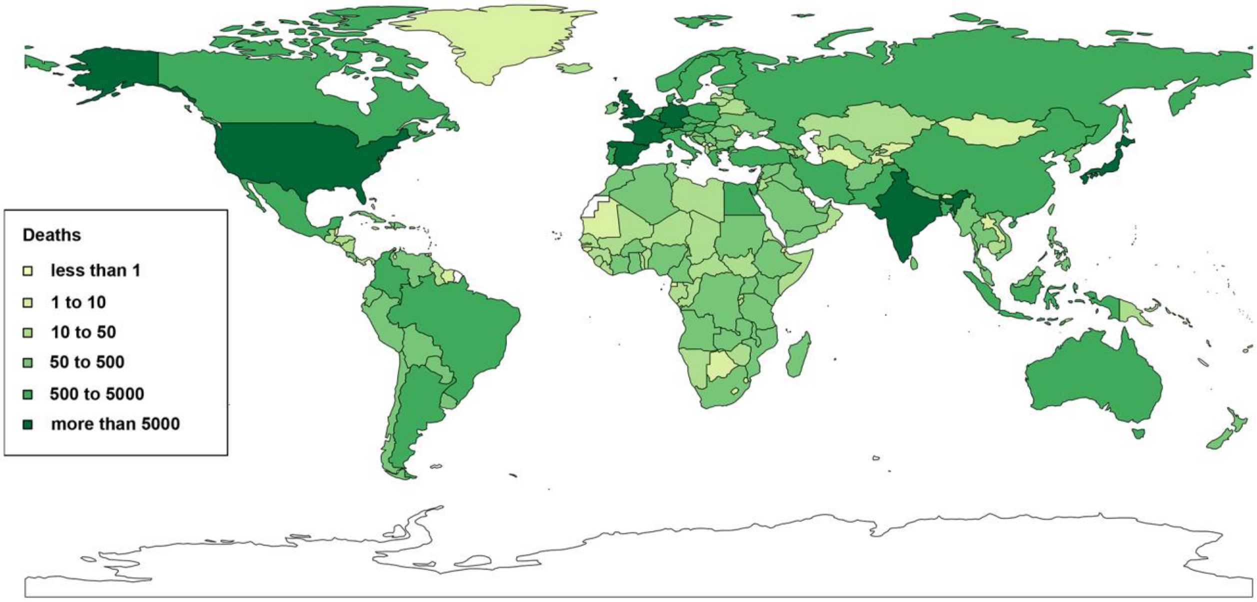

Supplementary Figure 7. The all-ages deaths of calcific aortic valve disease across 204 countries and territories in 2019.

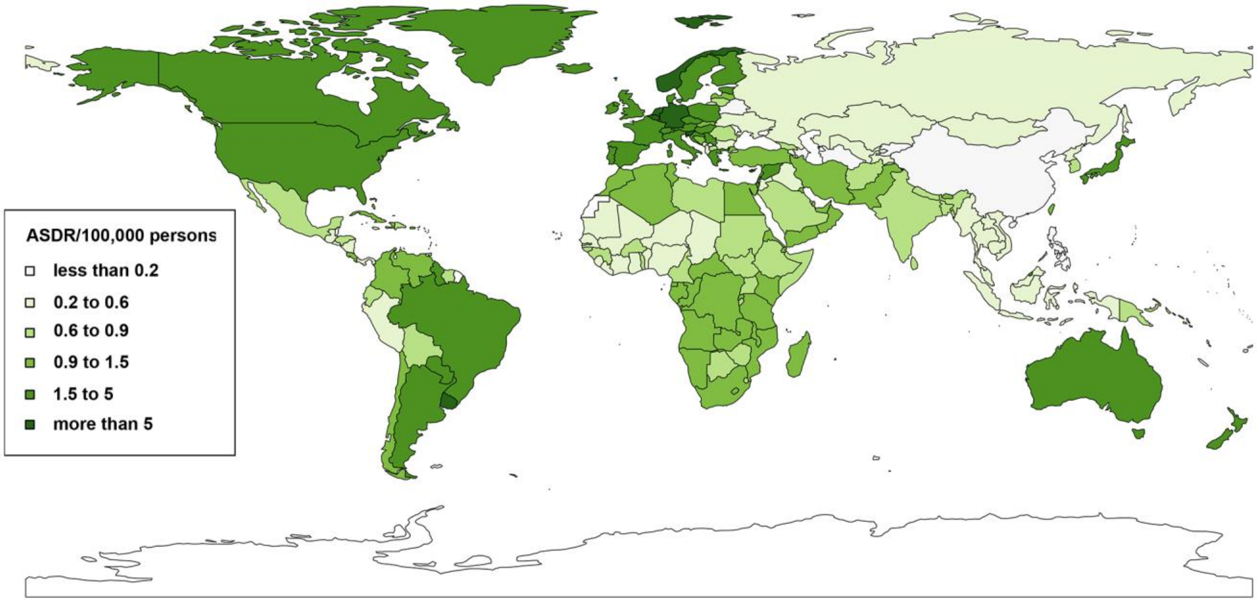

Supplementary Figure 8. The age standardized deaths rate of calcific aortic valve disease across 204 countries and territories in 2019.

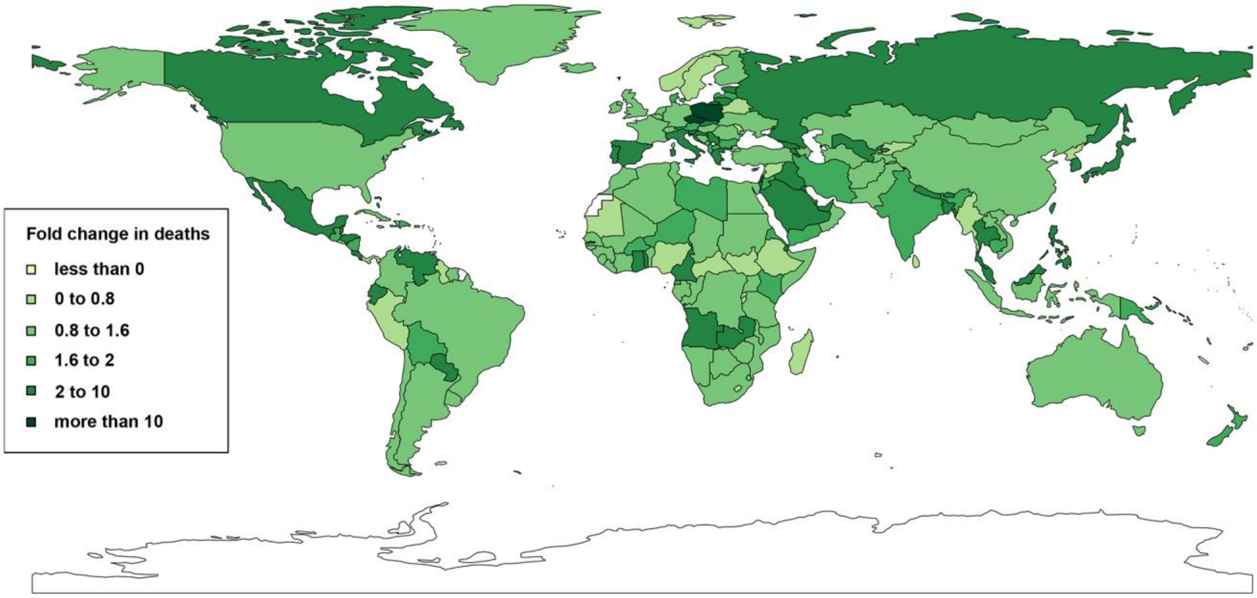

Supplementary Figure 9. The fold change in all-ages deaths of calcific aortic valve disease across 204 countries and territories between 1990 and 2019.

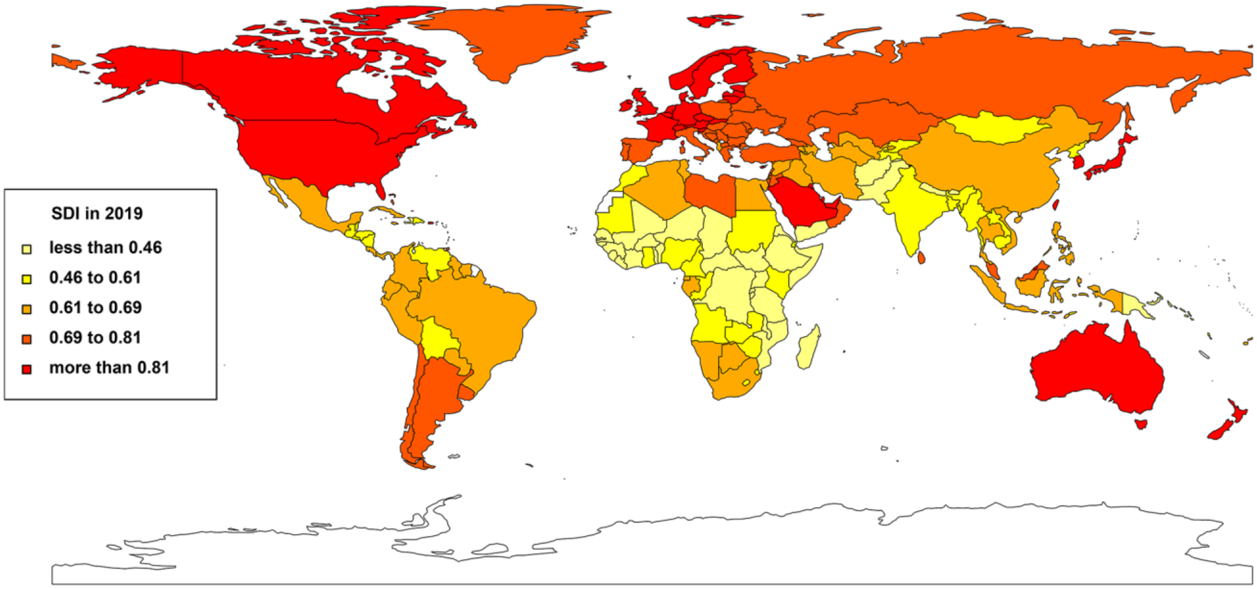

Supplementary Figure 10. The SDI across 204 countries and territories in 2019.
