# Supplementary material for: Changing epidemiology of calcific aortic valve disease: 30-year trends of incidence, prevalence, and deaths across 204 countries and territories
Source: Aging (Albany NY). 2021 May 11;13(9):12710–32. doi: 10.18632/aging.202942 (PMC8148466; doi:10.18632/aging.202942)
Supplement: Supplementary Table 1 [file aging-13-202942-s002.docx]

**Supplementary Table 1. The change of CAVD incidence between 1990 and 2019 and EAPC at 204 countries and territories**

|  |  | **All-ages Incidence** | | |  |  | **ASIR** | | |  |
| --- | --- | --- | --- | --- | --- | --- | --- | --- | --- | --- |
| **Location** | **1990 No. (95% UI)** | | **2019 No. (95% UI)** | **Change in Absolute Number (95% UI)** | | **1990 per 100,000 No. (95% UI)** | | **2019 per 100,000 No. (95% UI)** | **EAPC No. (95% CI)** | |
| **Afghanistan** | **38.67(33.18-45.6)** | | **70.75(58.09-86.81)** | **0.83(0.63-1.07)** | | **0.58(0.5-0.68)** | | **0.53(0.45-0.63)** | **-0.28(-12.32-13.4)** | |
| **Albania** | **58.09(50.49-66.72)** | | **523.5(423.21-634.97)** | **8.01(6.61-9.48)** | | **2.61(2.29-2.96)** | | **13.27(10.95-15.96)** | **5.77(1.04-10.72)** | |
| **Algeria** | **91.07(79.18-106.13)** | | **260.2(218.82-311.22)** | **1.86(1.6-2.15)** | | **0.82(0.73-0.93)** | | **0.76(0.65-0.91)** | **-0.26(-10.45-11.09)** | |
| **American Samoa** | **1.18(0.96-1.43)** | | **7.53(6-9.36)** | **5.39(4.18-6.9)** | | **5.03(4.08-6.1)** | | **13.7(11.11-16.81)** | **3.51(-0.07-7.23)** | |
| **Andorra** | **0.39(0.31-0.49)** | | **17.03(12.54-21.07)** | **42.62(32.1-53.83)** | | **0.73(0.6-0.9)** | | **12.63(9.34-15.64)** | **10.33(1.71-19.68)** | |
| **Angola** | **28.4(25.01-32.31)** | | **77.61(67.78-88.68)** | **1.73(1.57-1.9)** | | **0.95(0.85-1.06)** | | **0.92(0.83-1.03)** | **-0.08(-9.48-10.31)** | |
| **Antigua and Barbuda** | **0.8(0.74-0.87)** | | **2.71(2.35-3.08)** | **2.39(1.97-2.81)** | | **1.46(1.34-1.6)** | | **2.55(2.25-2.87)** | **1.94(-4.96-9.35)** | |
| **Argentina** | **775(614.49-1089.85)** | | **5322.72(4716.69-6056.79)** | **5.87(4.01-7.63)** | | **2.65(2.1-3.79)** | | **9.94(8.83-11.32)** | **4.66(-0.11-9.67)** | |
| **Armenia** | **13.38(10.58-16.78)** | | **131.6(107.37-164.55)** | **8.84(7.03-11.04)** | | **0.46(0.38-0.56)** | | **3.24(2.68-3.95)** | **6.96(-3.84-18.98)** | |
| **Australia** | **875.46(768.85-1000.2)** | | **17659.82(14847-20797.09)** | **19.17(16.1-22.95)** | | **4.65(4.14-5.24)** | | **46.56(39.52-54.82)** | **8.27(4.77-11.89)** | |
| **Austria** | **365.99(310.91-430.49)** | | **7270.25(6283.2-8223.11)** | **18.86(15.92-22.84)** | | **3.53(3.01-4.12)** | | **47.41(41.16-53.39)** | **9.37(5.37-13.53)** | |
| **Azerbaijan** | **26.17(20.46-33.21)** | | **306.82(243.55-373.62)** | **10.72(8.43-13.45)** | | **0.48(0.39-0.61)** | | **2.55(2.04-3.07)** | **5.9(-4.75-17.74)** | |
| **Bahamas** | **2.46(2.21-2.74)** | | **12.93(11.06-14.87)** | **4.25(3.73-4.86)** | | **1.55(1.41-1.7)** | | **2.98(2.6-3.37)** | **2.28(-4.35-9.37)** | |
| **Bahrain** | **7.13(5.77-8.55)** | | **58.71(45.57-74.61)** | **7.24(6.14-8.56)** | | **3.02(2.56-3.5)** | | **3.9(3.29-4.65)** | **0.89(-4.2-6.26)** | |
| **Bangladesh** | **331.37(289.93-379.86)** | | **973.94(854.1-1111.56)** | **1.94(1.77-2.12)** | | **0.82(0.73-0.93)** | | **0.84(0.74-0.95)** | **0.1(-9.87-11.17)** | |
| **Barbados** | **5.48(4.99-6.04)** | | **18.37(15.68-21.61)** | **2.35(1.92-2.89)** | | **1.94(1.77-2.14)** | | **4.08(3.5-4.73)** | **2.6(-3.28-8.83)** | |
| **Belarus** | **210.23(163.61-263.45)** | | **1939.18(1513.57-2462.08)** | **8.22(6.9-9.92)** | | **1.62(1.29-1.99)** | | **13.25(10.6-16.56)** | **7.51(1.63-13.73)** | |
| **Belgium** | **93.61(76.66-112.96)** | | **714.09(574.43-883.36)** | **6.63(5.47-8.11)** | | **0.62(0.51-0.74)** | | **3.39(2.73-4.2)** | **6.04(-3.42-16.42)** | |
| **Belize** | **0.99(0.87-1.12)** | | **9.84(8.04-12.75)** | **8.97(7.4-11.44)** | | **1.03(0.91-1.16)** | | **2.94(2.41-3.89)** | **3.7(-4.03-12.06)** | |
| **Benin** | **11.25(9.77-12.87)** | | **32.52(27.65-38.05)** | **1.89(1.67-2.13)** | | **0.55(0.48-0.63)** | | **0.63(0.55-0.72)** | **0.44(-11.32-13.77)** | |
| **Bermuda** | **4.24(3.62-5.05)** | | **21.13(17.75-25.14)** | **3.98(3.04-5)** | | **6.85(5.87-8.18)** | | **18.44(15.63-21.85)** | **3.47(0.39-6.65)** | |
| **Bhutan** | **1.75(1.49-2.05)** | | **7.01(5.59-9.58)** | **3.01(2.36-4.23)** | | **0.84(0.74-0.97)** | | **1.32(1.06-1.82)** | **1.57(-7.57-11.61)** | |
| **Bolivia (Plurinational State of)** | **25.18(22.28-28.64)** | | **128.96(111.79-149.78)** | **4.12(3.6-4.71)** | | **0.83(0.74-0.93)** | | **1.38(1.21-1.58)** | **1.74(-7.37-11.74)** | |
| **Bosnia and Herzegovina** | **147.09(122.92-174.93)** | | **924.47(750.63-1136.02)** | **5.29(4.46-6.46)** | | **3.4(2.94-3.96)** | | **16.92(13.97-20.38)** | **5.69(1.53-10.02)** | |
| **Botswana** | **3.91(3.42-4.48)** | | **15.27(12.91-17.85)** | **2.91(2.5-3.36)** | | **0.84(0.75-0.94)** | | **1.15(1.01-1.31)** | **1.07(-8.27-11.35)** | |
| **Brazil** | **1914.65(1641.08-2240.36)** | | **6396.68(5424.31-7484.51)** | **2.34(2.14-2.55)** | | **2.24(1.92-2.6)** | | **2.69(2.29-3.15)** | **0.63(-5.33-6.97)** | |
| **Brunei Darussalam** | **6.05(4.82-7.46)** | | **68.79(58.34-81.81)** | **10.36(8.68-12.64)** | | **5.46(4.2-6.89)** | | **18.5(15.71-21.74)** | **4.3(0.92-7.79)** | |
| **Bulgaria** | **958.51(765.01-1178.53)** | | **2023.76(1607.56-2495.24)** | **1.11(0.84-1.41)** | | **7.71(6.2-9.32)** | | **19.09(15.46-23.28)** | **3.18(0.24-6.2)** | |
| **Burkina Faso** | **21.55(18.26-25.06)** | | **60.96(53.1-70.47)** | **1.83(1.62-2.07)** | | **0.5(0.43-0.57)** | | **0.65(0.58-0.74)** | **0.95(-11.13-14.66)** | |
| **Burundi** | **17.71(15.68-19.92)** | | **25.05(21.43-29.32)** | **0.41(0.3-0.55)** | | **0.88(0.79-0.98)** | | **0.69(0.61-0.78)** | **-0.82(-11.02-10.54)** | |
| **Cabo Verde** | **1.17(1.01-1.34)** | | **3.2(2.76-3.7)** | **1.73(1.49-2)** | | **0.52(0.45-0.6)** | | **0.69(0.6-0.79)** | **0.95(-10.82-14.27)** | |
| **Cambodia** | **9.8(8.07-12.18)** | | **31.58(25.64-38.84)** | **2.22(1.86-2.66)** | | **0.26(0.22-0.31)** | | **0.28(0.24-0.34)** | **0.34(-16.55-20.65)** | |
| **Cameroon** | **36.02(30.65-42.21)** | | **113(97.12-131.22)** | **2.14(1.9-2.42)** | | **0.78(0.68-0.9)** | | **0.85(0.75-0.97)** | **0.32(-9.77-11.54)** | |
| **Canada** | **227.35(188.74-275.04)** | | **6482(5621.67-7429.8)** | **27.51(22.87-32.62)** | | **0.72(0.6-0.85)** | | **9.51(8.25-10.9)** | **9.33(0.64-18.77)** | |
| **Central African Republic** | **8.13(7.14-9.29)** | | **15.59(13.72-17.8)** | **0.92(0.82-1.03)** | | **0.91(0.81-1.01)** | | **0.95(0.85-1.05)** | **0.14(-9.32-10.59)** | |
| **Chad** | **13.15(11.25-15.37)** | | **30.66(26.31-35.89)** | **1.33(1.18-1.52)** | | **0.47(0.41-0.55)** | | **0.52(0.45-0.59)** | **0.33(-12.44-14.95)** | |
| **Chile** | **207.41(185.78-230.67)** | | **2705.77(2275.5-3221.83)** | **12.05(10.17-14.29)** | | **2.18(1.96-2.4)** | | **11.52(9.69-13.59)** | **5.91(0.75-11.34)** | |
| **China** | **2866.26(2280.67-3664.67)** | | **54965.28(43882.1-67453.88)** | **18.18(15.09-21.65)** | | **0.34(0.27-0.42)** | | **2.61(2.11-3.17)** | **7.31(-5.16-21.43)** | |
| **Colombia** | **248.18(206.4-282.09)** | | **1232.56(1083.27-1401.27)** | **3.97(3.34-4.93)** | | **1.38(1.15-1.56)** | | **2.3(2.03-2.62)** | **1.78(-5.36-9.46)** | |
| **Comoros** | **1.93(1.72-2.17)** | | **3.69(3.25-4.22)** | **0.92(0.79-1.05)** | | **1(0.9-1.11)** | | **0.85(0.75-0.96)** | **-0.57(-10-9.86)** | |
| **Congo** | **9.44(8.38-10.66)** | | **23.23(20.44-26.6)** | **1.46(1.31-1.64)** | | **1.11(1-1.23)** | | **1.11(1-1.24)** | **0.02(-8.65-9.51)** | |
| **Cook Islands** | **0.19(0.16-0.23)** | | **2.12(1.69-2.59)** | **10.06(8.23-12.24)** | | **1.57(1.31-1.87)** | | **8.3(6.68-10.06)** | **5.92(-0.14-12.34)** | |
| **Costa Rica** | **27.84(25.02-30.91)** | | **185.79(162.32-213.8)** | **5.67(5.02-6.42)** | | **1.56(1.41-1.72)** | | **3.48(3.05-4)** | **2.81(-3.67-9.73)** | |
| **Croatia** | **296.12(241.97-357.62)** | | **2971.86(2503.84-3452.15)** | **9.04(7.6-10.73)** | | **4.5(3.68-5.36)** | | **43.43(37.15-50.15)** | **8.13(4.57-11.81)** | |
| **Cuba** | **128.49(116.32-141.99)** | | **597.63(516.25-693.6)** | **3.65(3.12-4.25)** | | **1.26(1.15-1.39)** | | **3.39(2.95-3.9)** | **3.46(-3.58-11.01)** | |
| **Cyprus** | **5.68(4.54-7.07)** | | **210.48(167.44-260.04)** | **36.04(29.39-44.11)** | | **0.79(0.66-0.95)** | | **10.62(8.57-13.03)** | **9.38(1.08-18.36)** | |
| **Czechia** | **553.84(438.55-703.66)** | | **6051.4(4999.35-7331.12)** | **9.93(7.76-12.51)** | | **4.27(3.39-5.4)** | | **38.43(32.04-46.39)** | **7.87(4.21-11.65)** | |
| **Côte d'Ivoire** | **32.37(28.05-37.28)** | | **85.7(73.86-98.76)** | **1.65(1.44-1.9)** | | **0.7(0.62-0.78)** | | **0.72(0.63-0.81)** | **0.08(-10.67-12.14)** | |
| **Democratic People's Republic of Korea** | **33.09(26.74-41.25)** | | **115.58(94.31-142.78)** | **2.49(1.98-3.09)** | | **0.22(0.18-0.26)** | | **0.36(0.29-0.44)** | **1.75(-15.35-22.3)** | |
| **Democratic Republic of the Congo** | **109.8(94.34-127.84)** | | **263.4(229.21-304.44)** | **1.4(1.23-1.57)** | | **0.92(0.81-1.05)** | | **0.93(0.82-1.05)** | **0.03(-9.44-10.49)** | |
| **Denmark** | **47.36(38.54-58.16)** | | **1117.23(898.51-1392.35)** | **22.59(18.12-27.78)** | | **0.59(0.49-0.72)** | | **10.52(8.64-12.87)** | **10.42(0.9-20.84)** | |
| **Djibouti** | **1.06(0.93-1.23)** | | **4.13(3.55-4.8)** | **2.89(2.63-3.2)** | | **0.98(0.88-1.09)** | | **0.88(0.77-0.99)** | **-0.37(-9.8-10.04)** | |
| **Dominica** | **1.1(0.99-1.21)** | | **2.87(2.53-3.28)** | **1.61(1.31-1.95)** | | **1.56(1.42-1.71)** | | **3.27(2.9-3.72)** | **2.58(-3.95-9.55)** | |
| **Dominican Republic** | **32.6(28.69-37.01)** | | **191.04(166.86-219.85)** | **4.86(4.24-5.56)** | | **0.89(0.79-1)** | | **1.94(1.7-2.22)** | **2.73(-5.79-12.03)** | |
| **Ecuador** | **54.22(47.11-62.2)** | | **1277.86(1113.34-1458.8)** | **22.57(19.44-26.01)** | | **0.97(0.86-1.11)** | | **7.93(6.91-9.07)** | **7.51(-0.03-15.61)** | |
| **Egypt** | **500.32(395.07-612.82)** | | **1366.07(1099.21-1681.96)** | **1.73(1.51-2.03)** | | **1.65(1.32-2.02)** | | **2(1.61-2.42)** | **0.66(-6.24-8.07)** | |
| **El Salvador** | **17.18(14.32-20.69)** | | **62.68(49.53-81.84)** | **2.65(2.15-3.38)** | | **0.55(0.46-0.67)** | | **1.06(0.83-1.39)** | **2.26(-8.59-14.39)** | |
| **Equatorial Guinea** | **1.39(1.23-1.58)** | | **6.48(5.4-7.76)** | **3.66(3.08-4.22)** | | **0.89(0.8-0.99)** | | **1.49(1.27-1.74)** | **1.8(-7.01-11.44)** | |
| **Eritrea** | **6.48(5.63-7.49)** | | **15.7(13.62-18.1)** | **1.42(1.25-1.6)** | | **0.87(0.78-0.97)** | | **0.8(0.71-0.89)** | **-0.31(-10.22-10.7)** | |
| **Estonia** | **72.8(59.96-87.59)** | | **922.71(682.13-1194)** | **11.67(8.94-14.85)** | | **3.6(2.97-4.31)** | | **46.84(34.97-59.58)** | **9.25(5.29-13.36)** | |
| **Eswatini** | **3.37(2.65-4.46)** | | **10.48(7.55-13.44)** | **2.11(1.59-3.05)** | | **1.27(1.03-1.68)** | | **1.77(1.31-2.26)** | **1.15(-6.49-9.42)** | |
| **Ethiopia** | **106.41(87.01-130.04)** | | **231.15(193.22-278.1)** | **1.17(1.02-1.36)** | | **0.67(0.56-0.8)** | | **0.62(0.52-0.74)** | **-0.25(-11.44-12.36)** | |
| **Fiji** | **3.08(2.56-3.76)** | | **18.03(14.67-22.12)** | **4.86(3.98-5.9)** | | **0.98(0.82-1.19)** | | **2.21(1.83-2.68)** | **2.84(-5.25-11.62)** | |
| **Finland** | **248.23(203.67-300.79)** | | **3300.6(2412.18-4090.63)** | **12.3(9.08-16.33)** | | **3.48(2.86-4.17)** | | **30.59(22.93-37.64)** | **7.79(3.74-11.99)** | |
| **France** | **637.27(525.32-771.74)** | | **7634.41(5963.29-9549.14)** | **10.98(8.84-13.54)** | | **0.78(0.65-0.93)** | | **6.3(5.01-7.85)** | **7.49(-0.91-16.59)** | |
| **Gabon** | **5.68(5.06-6.35)** | | **24.82(20.56-29.83)** | **3.37(2.72-4.01)** | | **1.19(1.07-1.33)** | | **2.46(2.04-2.91)** | **2.52(-4.93-10.55)** | |
| **Gambia** | **2.16(1.86-2.51)** | | **6.36(5.48-7.27)** | **1.95(1.72-2.19)** | | **0.58(0.51-0.66)** | | **0.62(0.54-0.71)** | **0.26(-11.4-13.45)** | |
| **Georgia** | **80.34(64.44-98.72)** | | **294.62(248.16-346.42)** | **2.67(2.11-3.31)** | | **1.27(1.03-1.53)** | | **5.53(4.67-6.48)** | **5.22(-1.56-12.46)** | |
| **Germany** | **846.78(694.51-1022.64)** | | **18128.72(14796.73-22315.99)** | **20.41(16.81-24.63)** | | **0.7(0.58-0.83)** | | **11.26(9.25-13.68)** | **10.07(1.26-19.64)** | |
| **Ghana** | **28.95(24.12-34.76)** | | **106.62(90.66-124.01)** | **2.68(2.37-3.07)** | | **0.46(0.4-0.54)** | | **0.64(0.56-0.73)** | **1.15(-11.21-15.24)** | |
| **Greece** | **105.71(85.89-131.52)** | | **1836.28(1457.92-2290.91)** | **16.37(13.47-20.2)** | | **0.72(0.59-0.88)** | | **9.47(7.67-11.66)** | **9.3(0.62-18.73)** | |
| **Greenland** | **0.2(0.16-0.24)** | | **4.42(3.85-5.04)** | **21.65(17.87-25.63)** | | **0.62(0.52-0.75)** | | **7.8(6.81-8.79)** | **9.1(-0.18-19.25)** | |
| **Grenada** | **1.1(0.77-1.3)** | | **2.55(2.09-3.06)** | **1.3(0.81-2.29)** | | **1.47(1.03-1.72)** | | **2.16(1.8-2.58)** | **1.33(-5.73-8.92)** | |
| **Guam** | **2.47(2.08-2.94)** | | **26.89(21.36-32.95)** | **9.88(8.03-12.16)** | | **3.26(2.76-3.86)** | | **13.2(10.61-16.11)** | **4.94(0.65-9.42)** | |
| **Guatemala** | **19.63(16.26-23.65)** | | **82.35(68.31-97.8)** | **3.2(2.76-3.72)** | | **0.54(0.46-0.64)** | | **0.68(0.58-0.8)** | **0.81(-10.85-14.01)** | |
| **Guinea** | **16.44(14.13-19.21)** | | **35.02(30.6-40.1)** | **1.13(0.96-1.31)** | | **0.51(0.44-0.59)** | | **0.61(0.54-0.7)** | **0.67(-11.45-14.45)** | |
| **Guinea-Bissau** | **3.02(2.57-3.47)** | | **5.19(4.49-5.93)** | **0.72(0.58-0.9)** | | **0.72(0.62-0.82)** | | **0.67(0.59-0.75)** | **-0.25(-11.07-11.88)** | |
| **Guyana** | **7.08(6.15-8.21)** | | **23.5(19.56-26.81)** | **2.32(1.82-2.85)** | | **1.92(1.68-2.21)** | | **3.48(2.92-3.91)** | **2.07(-3.94-8.46)** | |
| **Haiti** | **42.01(34.22-47.39)** | | **101.86(79.19-126.59)** | **1.42(0.97-1.82)** | | **1.47(1.19-1.64)** | | **1.48(1.14-1.85)** | **0.03(-7.54-8.23)** | |
| **Honduras** | **13.83(11.86-16.02)** | | **60.08(51.45-68.94)** | **3.34(2.97-3.78)** | | **0.65(0.57-0.74)** | | **0.89(0.77-1.02)** | **1.09(-9.46-12.87)** | |
| **Hungary** | **1744.59(1511.6-2070.91)** | | **8293.27(7100.73-9884.45)** | **3.75(3.12-4.58)** | | **12.39(10.74-14.49)** | | **56.24(48.55-66.61)** | **5.36(3.14-7.61)** | |
| **Iceland** | **2(1.65-2.42)** | | **59.12(47.81-72.1)** | **28.56(23.5-35.44)** | | **0.7(0.58-0.85)** | | **11.36(9.29-13.71)** | **10.07(1.3-19.6)** | |
| **India** | **3023.11(2509.79-3662.36)** | | **9878.99(8321.07-11759.5)** | **2.27(2.07-2.47)** | | **0.86(0.72-1.02)** | | **0.99(0.83-1.17)** | **0.48(-9.04-10.99)** | |
| **Indonesia** | **250.44(200.8-311.55)** | | **1013.51(819.53-1261.67)** | **3.05(2.64-3.52)** | | **0.3(0.24-0.36)** | | **0.46(0.38-0.56)** | **1.55(-13.37-19.04)** | |
| **Iran (Islamic Republic of)** | **207.53(170.23-251.97)** | | **466.3(385.19-564.74)** | **1.25(1.09-1.42)** | | **0.74(0.62-0.89)** | | **0.58(0.48-0.7)** | **-0.84(-11.92-11.64)** | |
| **Iraq** | **130.7(106.73-159.45)** | | **252.01(202.26-313.87)** | **0.93(0.75-1.1)** | | **1.58(1.3-1.93)** | | **0.98(0.81-1.2)** | **-1.62(-9.8-7.29)** | |
| **Ireland** | **28.94(23.57-36.35)** | | **1031.37(762.69-1243.31)** | **34.64(25.56-43.61)** | | **0.74(0.61-0.9)** | | **14.23(10.67-17.1)** | **10.76(2.16-20.08)** | |
| **Israel** | **26.17(21.31-32.02)** | | **378.97(303.41-471.45)** | **13.48(11.22-16.19)** | | **0.57(0.47-0.69)** | | **3.32(2.69-4.09)** | **6.24(-3.55-17.01)** | |
| **Italy** | **4810(3996.89-5781.5)** | | **30306.88(25378.47-35943.09)** | **5.3(4.72-5.93)** | | **5.86(4.91-7.01)** | | **27.37(23.23-32.13)** | **5.46(2.26-8.75)** | |
| **Jamaica** | **11.91(10.2-13.91)** | | **45.59(36.27-61.03)** | **2.83(2.21-3.78)** | | **0.66(0.56-0.78)** | | **1.49(1.19-2)** | **2.84(-6.92-13.61)** | |
| **Japan** | **28092.56(23235.52-34105.37)** | | **71837.44(58159.53-87203.45)** | **1.56(1.26-1.87)** | | **17.1(14.22-20.52)** | | **27.4(23.13-32.7)** | **1.64(-0.46-3.78)** | |
| **Jordan** | **18.34(14.67-24.51)** | | **165.68(130.51-205.77)** | **8.03(6.43-9.88)** | | **1.24(1.03-1.64)** | | **1.97(1.6-2.4)** | **1.61(-5.96-9.79)** | |
| **Kazakhstan** | **130.5(104.13-163.12)** | | **1061.61(847.72-1291.64)** | **7.13(5.92-8.6)** | | **0.95(0.77-1.19)** | | **5.29(4.25-6.4)** | **6.09(-1.6-14.37)** | |
| **Kenya** | **64.09(53.61-76.55)** | | **219.53(183.08-263.21)** | **2.43(2.17-2.77)** | | **0.91(0.76-1.08)** | | **1.1(0.92-1.31)** | **0.66(-8.52-10.77)** | |
| **Kiribati** | **0.26(0.21-0.31)** | | **0.73(0.59-0.9)** | **1.84(1.5-2.24)** | | **0.79(0.66-0.94)** | | **1.04(0.87-1.27)** | **0.97(-8.72-11.69)** | |
| **Kuwait** | **56.85(45.85-70.79)** | | **271.88(210.92-340.11)** | **3.78(3.21-4.39)** | | **4.95(4.06-6)** | | **5.74(4.7-6.98)** | **0.51(-3.57-4.77)** | |
| **Kyrgyzstan** | **12.06(9.64-15)** | | **43.69(34.97-52.94)** | **2.62(2.09-3.28)** | | **0.38(0.31-0.47)** | | **0.77(0.63-0.93)** | **2.47(-10.37-17.15)** | |
| **Lao People's Democratic Republic** | **5.65(4.73-6.76)** | | **12.94(10.61-15.78)** | **1.29(1.03-1.56)** | | **0.32(0.28-0.37)** | | **0.33(0.28-0.39)** | **0.04(-15.44-18.35)** | |
| **Latvia** | **125.18(104.86-149.18)** | | **925.28(740.85-1152.39)** | **6.39(5.36-7.78)** | | **3.6(3.06-4.28)** | | **31.24(25.5-38.07)** | **7.73(3.76-11.86)** | |
| **Lebanon** | **23.79(18.25-34.79)** | | **63.42(47.77-84.8)** | **1.67(1.32-2.15)** | | **1.02(0.79-1.48)** | | **1.22(0.91-1.63)** | **0.61(-8.11-10.17)** | |
| **Lesotho** | **7.05(6.21-8.08)** | | **12.17(10.67-13.87)** | **0.73(0.6-0.88)** | | **0.86(0.77-0.97)** | | **1.1(0.98-1.23)** | **0.83(-8.51-11.12)** | |
| **Liberia** | **5.8(4.84-6.85)** | | **15.85(12.99-19.16)** | **1.73(1.46-2.07)** | | **0.53(0.45-0.63)** | | **0.65(0.55-0.76)** | **0.69(-11.12-14.07)** | |
| **Libya** | **35.91(28.98-43.56)** | | **90.83(72.25-113.56)** | **1.53(1.31-1.77)** | | **1.73(1.39-2.1)** | | **1.53(1.24-1.87)** | **-0.43(-7.62-7.32)** | |
| **Lithuania** | **114.2(92.83-137.8)** | | **1001.79(815.03-1213.44)** | **7.77(6.4-9.6)** | | **2.55(2.08-3.03)** | | **22.44(18.52-26.94)** | **7.79(3.08-12.71)** | |
| **Luxembourg** | **8.38(6.8-10.39)** | | **173.43(138.66-213.08)** | **19.71(16.91-24.02)** | | **1.59(1.31-1.95)** | | **18.85(15.13-23.14)** | **8.89(2.99-15.14)** | |
| **Madagascar** | **48.73(43.78-54.23)** | | **94.77(84.53-106.62)** | **0.94(0.85-1.07)** | | **1.09(0.98-1.2)** | | **1.09(0.98-1.2)** | **0.01(-8.75-9.61)** | |
| **Malawi** | **22.85(19.72-26.4)** | | **51.45(44.61-59.09)** | **1.25(1.12-1.4)** | | **0.75(0.67-0.85)** | | **0.85(0.74-0.96)** | **0.4(-9.79-11.75)** | |
| **Malaysia** | **40.49(34.45-47.97)** | | **315.26(263.76-376.01)** | **6.79(5.85-8.01)** | | **0.49(0.43-0.57)** | | **1.14(0.97-1.33)** | **2.94(-8.29-15.54)** | |
| **Maldives** | **0.29(0.24-0.36)** | | **2.64(2.04-3.27)** | **8.07(6.62-9.89)** | | **0.38(0.32-0.45)** | | **0.87(0.65-1.06)** | **2.89(-9.77-17.33)** | |
| **Mali** | **20.02(17.01-23.57)** | | **43.71(37.24-51.41)** | **1.18(1.02-1.35)** | | **0.5(0.43-0.58)** | | **0.51(0.44-0.58)** | **0.03(-12.58-14.45)** | |
| **Malta** | **1.85(1.5-2.3)** | | **68.15(57.67-80.78)** | **35.74(29.56-43.5)** | | **0.45(0.37-0.55)** | | **7.64(6.56-8.99)** | **10.22(-0.59-22.2)** | |
| **Marshall Islands** | **0.14(0.12-0.17)** | | **0.43(0.36-0.53)** | **2.08(1.71-2.54)** | | **0.93(0.77-1.11)** | | **1.29(1.07-1.57)** | **1.15(-7.74-10.89)** | |
| **Mauritania** | **6.99(6.1-8)** | | **16.6(13.81-19.77)** | **1.37(1.14-1.68)** | | **0.69(0.61-0.78)** | | **0.74(0.62-0.87)** | **0.23(-10.49-12.23)** | |
| **Mauritius** | **3.33(2.77-3.99)** | | **24.64(20.27-29.94)** | **6.41(5.37-7.98)** | | **0.49(0.42-0.57)** | | **1.35(1.13-1.61)** | **3.56(-7.47-15.91)** | |
| **Mexico** | **576.68(485.55-681.96)** | | **3502.89(2905.68-4101.12)** | **5.07(4.58-5.6)** | | **1.23(1.04-1.45)** | | **2.74(2.29-3.2)** | **2.79(-4.48-10.6)** | |
| **Micronesia (Federated States of)** | **0.63(0.52-0.77)** | | **1.92(1.56-2.38)** | **2.05(1.59-2.6)** | | **1.49(1.25-1.79)** | | **2.55(2.11-3.11)** | **1.87(-4.98-9.21)** | |
| **Monaco** | **0.69(0.54-0.87)** | | **13.76(11.1-17.12)** | **19.08(15.27-23.61)** | | **1.08(0.87-1.37)** | | **18.35(15.25-22.61)** | **10.26(3.12-17.89)** | |
| **Mongolia** | **4.32(3.51-5.29)** | | **25.34(20.74-30.62)** | **4.87(4.03-5.94)** | | **0.39(0.32-0.47)** | | **0.81(0.67-0.97)** | **2.55(-10.1-16.99)** | |
| **Montenegro** | **43.73(34.44-54.96)** | | **201.57(159.71-245.46)** | **3.61(2.99-4.34)** | | **6.57(5.2-8.16)** | | **22.74(18.3-27.51)** | **4.38(1.3-7.55)** | |
| **Morocco** | **86.83(74.31-102.11)** | | **213.94(177.96-259.32)** | **1.46(1.26-1.68)** | | **0.64(0.55-0.76)** | | **0.67(0.56-0.81)** | **0.14(-11.01-12.69)** | |
| **Mozambique** | **38.17(33.47-43.82)** | | **88.84(79.34-99.81)** | **1.33(1.18-1.49)** | | **0.81(0.72-0.9)** | | **0.98(0.88-1.09)** | **0.67(-9.05-11.43)** | |
| **Myanmar** | **56.11(46.44-68.43)** | | **129.27(103.6-159.02)** | **1.3(1.03-1.58)** | | **0.28(0.24-0.33)** | | **0.29(0.24-0.35)** | **0.16(-16.27-19.8)** | |
| **Namibia** | **5.7(5.03-6.44)** | | **12.42(10.97-14.03)** | **1.18(1.06-1.33)** | | **1(0.89-1.11)** | | **0.98(0.87-1.1)** | **-0.05(-9.2-10.02)** | |
| **Nauru** | **0.04(0.03-0.05)** | | **0.08(0.07-0.1)** | **1.18(0.9-1.53)** | | **1.23(1.04-1.46)** | | **1.81(1.52-2.14)** | **1.35(-6.34-9.69)** | |
| **Nepal** | **54.49(47.04-63.35)** | | **174.35(152.5-200.42)** | **2.2(1.97-2.42)** | | **0.71(0.63-0.81)** | | **0.89(0.79-1.01)** | **0.79(-9.47-12.21)** | |
| **Netherlands** | **121.77(101.2-147.21)** | | **976.83(790.13-1199.83)** | **7.02(5.86-8.37)** | | **0.62(0.52-0.74)** | | **2.96(2.43-3.6)** | **5.54(-3.97-16)** | |
| **New Zealand** | **384.94(317.15-458.05)** | | **2513.3(2095.54-2998.78)** | **5.53(4.92-6.22)** | | **10.11(8.39-11.95)** | | **32.99(27.83-39)** | **4.16(1.66-6.72)** | |
| **Nicaragua** | **9.2(7.75-10.85)** | | **41.25(33.57-50.41)** | **3.48(2.98-4.13)** | | **0.54(0.45-0.63)** | | **0.76(0.62-0.94)** | **1.23(-10.26-14.19)** | |
| **Niger** | **14.38(12.12-16.97)** | | **37.28(31.46-44.71)** | **1.59(1.39-1.81)** | | **0.5(0.43-0.58)** | | **0.48(0.41-0.55)** | **-0.16(-12.94-14.51)** | |
| **Nigeria** | **256.68(213.8-308.9)** | | **480.55(399.8-583.73)** | **0.87(0.8-0.95)** | | **0.58(0.49-0.69)** | | **0.51(0.43-0.62)** | **-0.4(-12.5-13.38)** | |
| **Niue** | **0.02(0.02-0.03)** | | **0.09(0.07-0.11)** | **2.61(2.04-3.27)** | | **1.09(0.93-1.28)** | | **3.93(3.24-4.74)** | **4.52(-2.85-12.46)** | |
| **North Macedonia** | **66.09(53.35-80.57)** | | **511.23(421.12-622.75)** | **6.73(5.43-8.71)** | | **3.25(2.64-3.94)** | | **16.36(13.51-19.71)** | **5.73(1.48-10.16)** | |
| **Northern Mariana Islands** | **0.69(0.57-0.84)** | | **6.49(5.06-8.11)** | **8.39(6.76-10.6)** | | **4.01(3.29-4.92)** | | **9.58(7.77-11.52)** | **3.05(-1.01-7.27)** | |
| **Norway** | **102.01(81.51-126.81)** | | **485.67(383.5-615.91)** | **3.76(3.21-4.53)** | | **1.51(1.23-1.83)** | | **5.21(4.16-6.58)** | **4.37(-1.96-11.1)** | |
| **Oman** | **8.14(7.07-9.31)** | | **40.97(32.21-51.11)** | **4.04(3.15-5.09)** | | **1.14(1.02-1.28)** | | **1.43(1.18-1.81)** | **0.79(-7.4-9.71)** | |
| **Pakistan** | **718.69(600.19-850.26)** | | **1336.11(1128.72-1570.79)** | **0.86(0.77-0.96)** | | **1.4(1.17-1.64)** | | **1.43(1.2-1.68)** | **0.08(-7.65-8.46)** | |
| **Palau** | **0.1(0.08-0.13)** | | **1.1(0.87-1.38)** | **9.75(7.5-12.31)** | | **1.05(0.84-1.29)** | | **4.3(3.47-5.32)** | **4.98(-2.46-12.98)** | |
| **Palestine** | **6.47(4.8-8.14)** | | **15.89(11.98-20.52)** | **1.45(1.01-1.91)** | | **0.72(0.52-0.92)** | | **0.6(0.45-0.79)** | **-0.64(-11.7-11.8)** | |
| **Panama** | **14.47(12.33-16.95)** | | **48.38(41.66-56.11)** | **2.34(1.92-2.84)** | | **0.94(0.8-1.1)** | | **1.15(0.99-1.32)** | **0.67(-8.35-10.58)** | |
| **Papua New Guinea** | **13.22(10.93-16.04)** | | **45.97(38.21-55.65)** | **2.48(2.14-2.85)** | | **0.84(0.7-0.99)** | | **0.96(0.8-1.13)** | **0.46(-9.2-11.14)** | |
| **Paraguay** | **35.01(31.84-38.65)** | | **188.49(170.9-207.3)** | **4.38(3.97-4.8)** | | **1.62(1.48-1.76)** | | **3.34(3.06-3.64)** | **2.53(-3.9-9.39)** | |
| **Peru** | **72.31(62.82-83.16)** | | **739.98(611.24-886.55)** | **9.23(7.77-11.02)** | | **0.59(0.52-0.67)** | | **2.22(1.83-2.67)** | **4.66(-5.19-15.53)** | |
| **Philippines** | **69.55(55.31-88.39)** | | **249.98(198.5-315.98)** | **2.59(2.27-2.98)** | | **0.26(0.21-0.33)** | | **0.33(0.27-0.4)** | **0.73(-15.59-20.2)** | |
| **Poland** | **2944.01(2367.53-3570.39)** | | **12398.84(10113.6-14847.98)** | **3.21(2.87-3.61)** | | **6.84(5.52-8.22)** | | **21.33(17.56-25.32)** | **4(0.96-7.13)** | |
| **Portugal** | **45.79(36.42-57.52)** | | **882.27(701.52-1090.87)** | **18.27(14.9-22.28)** | | **0.35(0.28-0.43)** | | **4.06(3.3-4.99)** | **8.85(-3.41-22.66)** | |
| **Puerto Rico** | **170.09(148.24-197.23)** | | **1131.86(915.92-1369.87)** | **5.65(4.54-6.75)** | | **4.77(4.16-5.53)** | | **23.76(19.11-29.19)** | **5.69(2.17-9.34)** | |
| **Qatar** | **30.38(23.67-38.19)** | | **251.74(195.85-313.6)** | **7.29(6.4-8.36)** | | **9.23(7.56-11.14)** | | **8.73(7.08-10.63)** | **-0.19(-3.32-3.05)** | |
| **Republic of Korea** | **2202.91(1820.13-2650.65)** | | **14718.91(12134.8-17671.54)** | **5.68(4.58-7.09)** | | **6.74(5.58-8.2)** | | **17.58(14.74-20.76)** | **3.36(0.24-6.57)** | |
| **Republic of Moldova** | **53.5(43.76-64.05)** | | **841.62(653.83-1055.2)** | **14.73(12.04-17.38)** | | **1.16(0.96-1.37)** | | **15.45(12.29-18.94)** | **9.35(2.45-16.71)** | |
| **Romania** | **2097.1(1707.36-2571.16)** | | **15734.3(12568.64-19709.52)** | **6.5(5.07-8.16)** | | **7.22(5.93-8.72)** | | **54.92(44.76-68.05)** | **7.25(4.42-10.15)** | |
| **Russian Federation** | **5138.41(4089.39-6194.68)** | | **54151.84(43433.2-65578.91)** | **9.54(8.78-10.5)** | | **2.89(2.32-3.53)** | | **25.93(20.93-31.18)** | **7.85(3.43-12.47)** | |
| **Rwanda** | **23.59(20.81-26.48)** | | **40.82(35.28-47.33)** | **0.73(0.61-0.86)** | | **1.03(0.91-1.15)** | | **0.88(0.77-1)** | **-0.54(-9.85-9.73)** | |
| **Saint Kitts and Nevis** | **0.48(0.43-0.53)** | | **2.25(1.91-2.66)** | **3.7(3.03-4.45)** | | **1.39(1.26-1.52)** | | **3.05(2.65-3.5)** | **2.75(-4.12-10.12)** | |
| **Saint Lucia** | **1.38(1.21-1.53)** | | **6.14(5.39-7.04)** | **3.45(2.93-4.1)** | | **1.66(1.46-1.82)** | | **2.8(2.49-3.18)** | **1.81(-4.71-8.77)** | |
| **Saint Vincent and the Grenadines** | **0.94(0.81-1.1)** | | **3.68(3.22-4.24)** | **2.91(2.32-3.65)** | | **1.34(1.15-1.55)** | | **2.71(2.4-3.08)** | **2.47(-4.59-10.06)** | |
| **Samoa** | **1.88(1.58-2.27)** | | **3.92(3.24-4.78)** | **1.08(0.84-1.39)** | | **2.25(1.89-2.69)** | | **2.61(2.17-3.14)** | **0.51(-5.49-6.89)** | |
| **San Marino** | **0.22(0.17-0.27)** | | **6.56(5.11-7.85)** | **29.25(24.05-35.42)** | | **0.67(0.53-0.82)** | | **12(9.34-14.36)** | **10.48(1.47-20.29)** | |
| **Sao Tome and Principe** | **0.33(0.28-0.4)** | | **0.79(0.66-0.92)** | **1.39(1.17-1.64)** | | **0.53(0.46-0.62)** | | **0.67(0.58-0.77)** | **0.79(-10.99-14.12)** | |
| **Saudi Arabia** | **68.23(51.68-92.61)** | | **862.72(668.29-1076.3)** | **11.64(9.02-14.44)** | | **1.02(0.8-1.39)** | | **2.91(2.4-3.47)** | **3.67(-4.07-12.05)** | |
| **Senegal** | **16.64(14.12-19.51)** | | **39.21(33.16-46.46)** | **1.36(1.2-1.54)** | | **0.5(0.43-0.58)** | | **0.5(0.43-0.58)** | **0.01(-12.62-14.47)** | |
| **Serbia** | **527.86(455.78-608.37)** | | **3706.98(3131.25-4426.46)** | **6.02(4.98-7.38)** | | **4.27(3.76-4.85)** | | **28.27(24.09-33.46)** | **6.74(3.06-10.55)** | |
| **Seychelles** | **0.39(0.34-0.45)** | | **2.2(1.68-2.89)** | **4.68(3.59-6.09)** | | **0.69(0.6-0.8)** | | **1.8(1.39-2.34)** | **3.33(-6.08-13.69)** | |
| **Sierra Leone** | **10.58(9.18-12.12)** | | **21.52(18.44-25.03)** | **1.03(0.9-1.19)** | | **0.54(0.48-0.62)** | | **0.56(0.48-0.64)** | **0.1(-12-13.86)** | |
| **Singapore** | **171.65(142.44-206.77)** | | **1976.18(1606.22-2391.68)** | **10.51(9.38-11.84)** | | **7.02(5.85-8.49)** | | **24.41(19.96-29.29)** | **4.39(1.41-7.46)** | |
| **Slovakia** | **162.38(132.24-201.46)** | | **1824.48(1541.47-2171.36)** | **10.24(8.25-12.55)** | | **2.78(2.26-3.43)** | | **22.65(19.02-26.87)** | **7.51(2.98-12.23)** | |
| **Slovenia** | **201.71(162.47-249.78)** | | **2114.86(1517.01-2755.28)** | **9.48(7.12-12.85)** | | **8.23(6.64-10.17)** | | **62.21(45.88-79.83)** | **7.22(4.57-9.95)** | |
| **Solomon Islands** | **1.05(0.86-1.28)** | | **3.37(2.77-4.06)** | **2.21(1.9-2.56)** | | **0.96(0.79-1.15)** | | **1.12(0.94-1.34)** | **0.53(-8.49-10.45)** | |
| **Somalia** | **17(14.76-19.59)** | | **34.95(30.09-40.88)** | **1.06(0.9-1.22)** | | **0.86(0.76-0.96)** | | **0.7(0.62-0.79)** | **-0.72(-10.98-10.71)** | |
| **South Africa** | **391.46(330.16-462.23)** | | **4529.28(3612.05-5584.28)** | **10.57(8.78-12.62)** | | **1.84(1.56-2.19)** | | **8.83(7.04-10.76)** | **5.55(-0.07-11.48)** | |
| **South Sudan** | **18.57(16.41-21.15)** | | **26.12(22.24-30.59)** | **0.41(0.29-0.52)** | | **0.92(0.82-1.03)** | | **0.81(0.71-0.92)** | **-0.45(-10.2-10.37)** | |
| **Spain** | **343.52(280.42-427.45)** | | **5645.53(4544.73-6886.05)** | **15.43(12.85-18.38)** | | **0.66(0.55-0.8)** | | **6.76(5.44-8.26)** | **8.34(-0.69-18.19)** | |
| **Sri Lanka** | **57.99(46.98-67.64)** | | **154.13(129.68-181.31)** | **1.66(1.41-2.01)** | | **0.59(0.49-0.68)** | | **0.59(0.51-0.69)** | **0.02(-11.67-13.25)** | |
| **Sudan** | **58.62(50.79-67.84)** | | **124.09(104.37-147.06)** | **1.12(0.94-1.32)** | | **0.65(0.57-0.75)** | | **0.63(0.54-0.74)** | **-0.13(-11.39-12.56)** | |
| **Suriname** | **3.03(2.71-3.4)** | | **15.17(13.15-17.54)** | **4.01(3.51-4.58)** | | **1.17(1.05-1.3)** | | **2.4(2.1-2.74)** | **2.51(-5.02-10.64)** | |
| **Sweden** | **427.41(339.43-540.84)** | | **3764.91(3049.82-4642.64)** | **7.81(6.72-9.17)** | | **3.04(2.46-3.76)** | | **20.75(17.04-25.3)** | **6.84(2.5-11.37)** | |
| **Switzerland** | **117.22(95.13-148.55)** | | **2729.98(2309.89-3178.81)** | **22.29(18.59-27.03)** | | **1.16(0.95-1.48)** | | **18(15.31-20.94)** | **9.9(3.02-17.24)** | |
| **Syrian Arab Republic** | **90.22(76.89-101.86)** | | **128.11(111.4-146.21)** | **0.42(0.26-0.61)** | | **1.72(1.47-1.93)** | | **1.04(0.93-1.16)** | **-1.72(-9.63-6.88)** | |
| **Taiwan (Province of China)** | **71.23(56.87-89.23)** | | **1560.15(1302.8-1885.7)** | **20.9(17.34-25.37)** | | **0.55(0.45-0.66)** | | **3.94(3.3-4.71)** | **7.05(-2.91-18.02)** | |
| **Tajikistan** | **8.57(6.83-10.72)** | | **29.84(23.79-36.98)** | **2.48(1.98-3.1)** | | **0.28(0.23-0.35)** | | **0.44(0.36-0.52)** | **1.51(-13.78-19.51)** | |
| **Thailand** | **108.52(91-129.67)** | | **1050.08(886.22-1252.09)** | **8.68(7.4-10.32)** | | **0.35(0.3-0.4)** | | **1(0.85-1.17)** | **3.72(-9.23-18.51)** | |
| **Timor-Leste** | **0.63(0.51-0.8)** | | **2.28(1.87-2.77)** | **2.6(2.14-3.13)** | | **0.28(0.23-0.33)** | | **0.31(0.26-0.36)** | **0.37(-15.94-19.83)** | |
| **Togo** | **7.59(6.56-8.79)** | | **25.19(21.93-28.97)** | **2.32(2.12-2.54)** | | **0.58(0.51-0.66)** | | **0.65(0.58-0.73)** | **0.43(-11.1-13.47)** | |
| **Tokelau** | **0.01(0.01-0.01)** | | **0.03(0.02-0.03)** | **1.41(1.07-1.88)** | | **0.77(0.65-0.9)** | | **1.83(1.53-2.19)** | **3.05(-6-12.98)** | |
| **Tonga** | **0.82(0.69-0.99)** | | **2.56(2.11-3.09)** | **2.1(1.72-2.52)** | | **1.6(1.35-1.9)** | | **3.21(2.64-3.88)** | **2.42(-4.06-9.35)** | |
| **Trinidad and Tobago** | **10.55(9.08-12.71)** | | **57.7(46.68-71.38)** | **4.47(3.61-5.51)** | | **1.29(1.11-1.54)** | | **3.17(2.61-3.89)** | **3.15(-3.88-10.7)** | |
| **Tunisia** | **40.03(34.39-47.06)** | | **106.3(88.57-128.55)** | **1.66(1.43-1.93)** | | **0.8(0.7-0.93)** | | **0.83(0.7-1.02)** | **0.15(-9.9-11.33)** | |
| **Turkey** | **478.07(426.09-533.8)** | | **1545.85(1389.78-1730.6)** | **2.23(2.01-2.48)** | | **1.4(1.26-1.55)** | | **1.76(1.6-1.95)** | **0.79(-6.63-8.81)** | |
| **Turkmenistan** | **8.41(6.69-10.59)** | | **124.03(99.95-150.01)** | **13.75(11.16-16.92)** | | **0.41(0.33-0.5)** | | **2.52(2.04-3.03)** | **6.5(-5.01-19.41)** | |
| **Tuvalu** | **0.04(0.03-0.05)** | | **0.12(0.1-0.14)** | **1.98(1.69-2.33)** | | **0.68(0.58-0.79)** | | **1.17(0.97-1.38)** | **1.89(-8.1-12.96)** | |
| **Uganda** | **40.27(35.21-46.55)** | | **89.78(78.22-104.49)** | **1.23(1.11-1.36)** | | **0.75(0.66-0.85)** | | **0.77(0.68-0.88)** | **0.1(-10.27-11.66)** | |
| **Ukraine** | **2218.3(1761.44-2708.9)** | | **8632.81(6820.62-10662.16)** | **2.89(2.56-3.32)** | | **3.26(2.61-3.92)** | | **13.38(10.64-16.36)** | **4.99(0.7-9.47)** | |
| **United Arab Emirates** | **25.81(19.59-33.34)** | | **625.63(467.92-818.87)** | **23.24(19.59-27.86)** | | **2.92(2.35-3.56)** | | **6.05(4.97-7.4)** | **2.54(-2.28-7.6)** | |
| **United Kingdom** | **7550.59(6059.49-9356.9)** | | **29659.88(24842.48-35286.42)** | **2.93(2.53-3.49)** | | **9.25(7.5-11.4)** | | **25.86(21.74-30.53)** | **3.61(0.96-6.33)** | |
| **United Republic of Tanzania** | **73.78(64.31-84.6)** | | **190.42(165.95-220.43)** | **1.58(1.41-1.77)** | | **0.86(0.77-0.97)** | | **0.91(0.8-1.04)** | **0.17(-9.51-10.88)** | |
| **United States of America** | **48676.7(39529.86-59738.32)** | | **117079.89(100604.05-135189.82)** | **1.41(1.12-1.75)** | | **15.77(12.89-19.16)** | | **21.52(18.69-24.6)** | **1.08(-1.16-3.37)** | |
| **United States Virgin Islands** | **3.33(2.93-3.86)** | | **23.97(19.74-28.65)** | **6.2(5.18-7.33)** | | **3.82(3.41-4.33)** | | **15.62(12.96-18.56)** | **4.98(1-9.11)** | |
| **Uruguay** | **105.44(81.14-136.57)** | | **698.52(616.12-793.66)** | **5.62(4.19-7.49)** | | **2.79(2.17-3.59)** | | **12.95(11.39-14.81)** | **5.43(0.83-10.24)** | |
| **Uzbekistan** | **46.22(36.68-57.13)** | | **627.92(509.75-745.22)** | **12.59(10.15-15.53)** | | **0.38(0.31-0.47)** | | **2.17(1.81-2.55)** | **6.19(-5.7-19.59)** | |
| **Vanuatu** | **0.62(0.51-0.76)** | | **2.31(1.92-2.77)** | **2.7(2.35-3.11)** | | **1.1(0.91-1.31)** | | **1.36(1.13-1.63)** | **0.76(-7.61-9.89)** | |
| **Venezuela (Bolivarian Republic of)** | **123.67(111.58-137.64)** | | **534.37(466.56-611.16)** | **3.32(2.92-3.73)** | | **1.26(1.14-1.38)** | | **1.75(1.54-1.99)** | **1.16(-6.53-9.47)** | |
| **Viet Nam** | **108.88(92.13-129.85)** | | **301.88(248.08-367.25)** | **1.77(1.44-2.19)** | | **0.29(0.25-0.34)** | | **0.33(0.28-0.4)** | **0.42(-15.38-19.16)** | |
| **Yemen** | **31.45(27.22-36.52)** | | **81.69(70.29-95.55)** | **1.6(1.42-1.79)** | | **0.65(0.57-0.75)** | | **0.6(0.53-0.7)** | **-0.25(-11.63-12.6)** | |
| **Zambia** | **18.94(16.35-21.9)** | | **48.63(42.1-56.43)** | **1.57(1.4-1.75)** | | **0.83(0.73-0.93)** | | **0.88(0.78-0.99)** | **0.21(-9.65-11.16)** | |
| **Zimbabwe** | **29.26(25.52-33.49)** | | **57.3(50.59-65.08)** | **0.96(0.84-1.1)** | | **0.87(0.77-0.97)** | | **0.96(0.86-1.07)** | **0.35(-9.21-10.92)** | |
| **Abbreviations: CAVD, calcific aortic valve disease; EAPC, estimated annual percentage change; ASIR, age standardized incidence rate; UI, uncertainty interval; CI, confidence interval.** | | | | | | | | | | |
