# Supplementary material for: Changing epidemiology of calcific aortic valve disease: 30-year trends of incidence, prevalence, and deaths across 204 countries and territories
Source: Aging (Albany NY). 2021 May 11;13(9):12710–32. doi: 10.18632/aging.202942 (PMC8148466; doi:10.18632/aging.202942)
Supplement: Supplementary Table 2 [file aging-13-202942-s003.docx]

**Supplementary Table 2. The population, incidence cases, prevalence cases and deaths across 204 countries and territories in 2019.**

| **Location** | **Population** | **Incidence Cases** | **Prevalence Cases** | **Deaths** |
| --- | --- | --- | --- | --- |
|  | **2019 No./10,000 (95% UI)** | **2019 No. (95% UI)** | **2019 No. (95% UI)** | **2019 No. (95% UI)** |
| **Afghanistan** | **3827.75(2616.18-5046.86)** | **70.75(58.09-86.81)** | **170.37(124.87-228.24)** | **112.26(57.48-179.7)** |
| **Albania** | **272.04(241.83-302.18)** | **523.5(423.21-634.97)** | **10141.92(8376.99-12306.05)** | **23.25(16.18-31.33)** |
| **Algeria** | **4184.73(3602.05-4746.19)** | **260.2(218.82-311.22)** | **1728.94(1336.93-2185.01)** | **294.01(191.27-400.16)** |
| **American Samoa** | **5.55(4.84-6.28)** | **7.53(6-9.36)** | **91.13(71.72-114.05)** | **0.42(0.34-0.52)** |
| **Andorra** | **8.31(7.62-8.97)** | **17.03(12.54-21.07)** | **237.12(179.76-294.75)** | **3.13(2.27-4.11)** |
| **Angola** | **3013.85(2705.48-3311.66)** | **77.61(67.78-88.68)** | **148.99(114.56-189.88)** | **86.54(65.52-114.66)** |
| **Antigua and Barbuda** | **8.85(7.76-9.89)** | **2.71(2.35-3.08)** | **31.02(25.53-36.82)** | **0.85(0.7-1.08)** |
| **Argentina** | **4511.53(3950.72-5107.34)** | **5322.72(4716.69-6056.79)** | **55366.98(47035.94-66507.15)** | **2165.88(1869.33-2414.45)** |
| **Armenia** | **301.97(265.19-338.59)** | **131.6(107.37-164.55)** | **2287.98(1858.52-2807.21)** | **14.93(12.15-17.93)** |
| **Australia** | **2456.81(2251.01-2677.92)** | **17659.82(14847-20797.09)** | **288496.62(243984.57-345487.19)** | **1434.82(1166.87-1648.12)** |
| **Austria** | **891.62(816.96-966.65)** | **7270.25(6283.2-8223.11)** | **121170.14(104957.54-137387.47)** | **979.92(787.42-1133)** |
| **Azerbaijan** | **1027.87(895.35-1164.01)** | **306.82(243.55-373.62)** | **4647.19(3661.18-5738.56)** | **15.8(12.44-20.21)** |
| **Bahamas** | **37.69(33.04-42.47)** | **12.93(11.06-14.87)** | **156.2(127.86-186.95)** | **3.32(2.7-4.15)** |
| **Bahrain** | **144.27(125.48-160.59)** | **58.71(45.57-74.61)** | **603.32(456.76-768.22)** | **6.86(5.23-8.89)** |
| **Bangladesh** | **15925.98(14119.98-17785.28)** | **973.94(854.1-1111.56)** | **2240.38(1745.33-2814.81)** | **932.92(615.85-1326.07)** |
| **Barbados** | **29.78(26.36-33.46)** | **18.37(15.68-21.61)** | **276.15(222.44-331.96)** | **5.37(4.44-6.42)** |
| **Belarus** | **950.08(834.54-1067.77)** | **1939.18(1513.57-2462.08)** | **35853.75(28627.66-44269.13)** | **29.07(22.3-37.94)** |
| **Belgium** | **1141.92(1053.69-1231.8)** | **714.09(574.43-883.36)** | **8835.75(6943.98-11221.16)** | **1703.05(1323.47-1960.29)** |
| **Belize** | **41.01(35.88-45.91)** | **9.84(8.04-12.75)** | **124.89(99.65-159.65)** | **1.32(1.09-1.69)** |
| **Benin** | **1266.58(1131.69-1398.32)** | **32.52(27.65-38.05)** | **98.98(75.61-128.34)** | **29.61(17.19-46.7)** |
| **Bermuda** | **6.4(5.83-6.97)** | **21.13(17.75-25.14)** | **347.25(291-414.43)** | **4.74(3.86-5.78)** |
| **Bhutan** | **75.42(69.7-81.54)** | **7.01(5.59-9.58)** | **29.64(21.72-39.88)** | **4.32(2.91-5.94)** |
| **Bolivia (Plurinational State of)** | **1201.17(1064.17-1341.82)** | **128.96(111.79-149.78)** | **1442.21(1184.09-1734.73)** | **71.36(50.41-100.18)** |
| **Bosnia and Herzegovina** | **330(294.96-364.92)** | **924.47(750.63-1136.02)** | **17469.25(14381.32-21149.95)** | **80.81(61.75-106.79)** |
| **Botswana** | **233.87(208.47-260.78)** | **15.27(12.91-17.85)** | **90.07(68.43-114.02)** | **9.24(6.24-13.95)** |
| **Brazil** | **21666.48(18987.92-24250.24)** | **6396.68(5424.31-7484.51)** | **57152.9(45926.57-70348.39)** | **3467.29(3000.8-4002.58)** |
| **Brunei Darussalam** | **43.71(38.2-49.17)** | **68.79(58.34-81.81)** | **899.49(747.64-1088.82)** | **4.99(4.08-5.84)** |
| **Bulgaria** | **693.46(636-755.39)** | **2023.76(1607.56-2495.24)** | **48313.6(39016.85-58507.8)** | **69.08(52.01-88.83)** |
| **Burkina Faso** | **2269.18(1938.33-2617.33)** | **60.96(53.1-70.47)** | **153.5(118.91-195.88)** | **65.1(37.37-101.62)** |
| **Burundi** | **1193.44(1030.46-1353.21)** | **25.05(21.43-29.32)** | **40.75(30.5-53.04)** | **28.53(19.13-40.71)** |
| **Cabo Verde** | **56.36(49.46-63.21)** | **3.2(2.76-3.7)** | **16.3(12.78-20.31)** | **3.12(2.42-4)** |
| **Cambodia** | **1660.31(1420.62-1886.77)** | **31.58(25.64-38.84)** | **137.41(99.61-188.34)** | **22.67(16.52-34.83)** |
| **Cameroon** | **2910.19(2478.3-3360.48)** | **113(97.12-131.22)** | **450.61(344.02-577.48)** | **82.27(49.37-131.72)** |
| **Canada** | **3651.98(3333.15-3959.98)** | **6482(5621.67-7429.8)** | **67764.69(56039.81-80665.54)** | **2358.59(1900.88-2649.44)** |
| **Central African Republic** | **529.99(445.93-619.26)** | **15.59(13.72-17.8)** | **19.47(15.18-24.25)** | **16.8(9-26.99)** |
| **Chad** | **1639.89(1432.74-1868.01)** | **30.66(26.31-35.89)** | **69.68(53.88-89.53)** | **30.73(17.01-49.8)** |
| **Chile** | **1819.84(1675.35-1961.72)** | **2705.77(2275.5-3221.83)** | **39520.69(32889.04-47405.09)** | **334.95(293.92-384.51)** |
| **China** | **142235.04(123930.24-159706.35)** | **54965.28(43882.1-67453.88)** | **867916.68(687947.96-1064921.33)** | **2798.63(2224.69-3395.63)** |
| **Colombia** | **4777.67(4417.43-5153.95)** | **1232.56(1083.27-1401.27)** | **14538.68(11904.3-17575.66)** | **663.55(478.02-933.29)** |
| **Comoros** | **71.44(59.33-83.72)** | **3.69(3.25-4.22)** | **7.65(5.9-9.76)** | **3.6(2.65-4.88)** |
| **Congo** | **526.58(450.71-600.88)** | **23.23(20.44-26.6)** | **49.37(37.81-62.64)** | **22.35(15.94-31.39)** |
| **Cook Islands** | **1.8(1.66-1.96)** | **2.12(1.69-2.59)** | **29.89(23.04-36.94)** | **0.07(0.06-0.09)** |
| **Costa Rica** | **471.67(416.48-527.15)** | **185.79(162.32-213.8)** | **2541.53(2115.02-3037.08)** | **67.39(51.3-86.75)** |
| **Croatia** | **424.79(374.84-476.42)** | **2971.86(2503.84-3452.15)** | **63553.55(54339.03-73922.25)** | **297.74(207.67-381.75)** |
| **Cuba** | **1135.85(1009.47-1273.88)** | **597.63(516.25-693.6)** | **8981.54(7468.06-10721.56)** | **195.39(152.26-258.03)** |
| **Cyprus** | **131.35(116.21-147.62)** | **210.48(167.44-260.04)** | **2657(2136.06-3279.02)** | **134.17(112.16-158.02)** |
| **Czechia** | **1064.35(977.91-1150.01)** | **6051.4(4999.35-7331.12)** | **135355.52(112016.5-163024)** | **597.9(436.91-776.84)** |
| **Côte d'Ivoire** | **2617.15(2357.31-2887.3)** | **85.7(73.86-98.76)** | **242.54(188.2-309.12)** | **60.31(35.96-93.73)** |
| **Democratic People's Republic of Korea** | **2623.29(2262.89-2991.05)** | **115.58(94.31-142.78)** | **1190.27(915.91-1530.54)** | **62.24(46.01-85.92)** |
| **Democratic Republic of the Congo** | **8767.04(6174.86-11259.04)** | **263.4(229.21-304.44)** | **444.97(342.2-575.93)** | **269.52(173.94-399.89)** |
| **Denmark** | **580.27(533-626.22)** | **1117.23(898.51-1392.35)** | **16873.48(13725.97-20994.02)** | **603.66(485.47-686.82)** |
| **Djibouti** | **120.28(105.05-136.27)** | **4.13(3.55-4.8)** | **10.95(8.35-14.11)** | **4.21(2.84-6.16)** |
| **Dominica** | **6.87(6.01-7.71)** | **2.87(2.53-3.28)** | **33.29(27.25-40.31)** | **0.93(0.72-1.19)** |
| **Dominican Republic** | **1088.19(962.98-1227.98)** | **191.04(166.86-219.85)** | **2205.61(1823.69-2649.79)** | **75.12(57.57-97.24)** |
| **Ecuador** | **1758.84(1540.39-1974.99)** | **1277.86(1113.34-1458.8)** | **20348.22(17505.14-23403.86)** | **122.5(93.38-159.79)** |
| **Egypt** | **9906.96(9057.19-10751.58)** | **1366.07(1099.21-1681.96)** | **9705.76(7368.99-12609.96)** | **657.7(471.27-928.72)** |
| **El Salvador** | **625.61(539.31-710.29)** | **62.68(49.53-81.84)** | **842.83(651.44-1101.43)** | **16.14(12.13-20.94)** |
| **Equatorial Guinea** | **141.98(129.06-155.29)** | **6.48(5.4-7.76)** | **20.83(15.57-26.94)** | **4.27(2.76-6.16)** |
| **Eritrea** | **671.12(478.06-859.57)** | **15.7(13.62-18.1)** | **26.52(20.18-34.3)** | **18.5(11.91-28.07)** |
| **Estonia** | **131.24(120.44-141.55)** | **922.71(682.13-1194)** | **18715.11(14075.47-23511.91)** | **80.46(59.07-108.48)** |
| **Eswatini** | **114.21(105.11-123.03)** | **10.48(7.55-13.44)** | **65.62(44.87-89.49)** | **3.67(2.38-5.63)** |
| **Ethiopia** | **10759.12(9202.43-12277.62)** | **231.15(193.22-278.1)** | **512.23(387.25-657.25)** | **241.07(177.1-333.91)** |
| **Fiji** | **91.12(83.88-98.42)** | **18.03(14.67-22.12)** | **158.01(123.96-199.49)** | **2.42(1.89-3.04)** |
| **Finland** | **553.41(508.65-599.2)** | **3300.6(2412.18-4090.63)** | **53982.03(40850.42-67091.72)** | **744.08(594.53-863.21)** |
| **France** | **6620.43(6009.38-7243.37)** | **7634.41(5963.29-9549.14)** | **109064.62(86214.37-138503.2)** | **8028.8(6387-9224.84)** |
| **Gabon** | **175(156.63-193.92)** | **24.82(20.56-29.83)** | **112.43(84.31-146.91)** | **10.41(7.95-13.41)** |
| **Gambia** | **224.59(202.8-247.62)** | **6.36(5.48-7.27)** | **18.2(14.16-23.08)** | **6.03(3.84-9.1)** |
| **Georgia** | **366.48(330.62-404.33)** | **294.62(248.16-346.42)** | **5384.31(4500.68-6431.52)** | **44.11(30.79-67)** |
| **Germany** | **8491.41(7768.86-9221.95)** | **18128.72(14796.73-22315.99)** | **287038.54(235176.32-348616.54)** | **13154.48(11098.62-15131.62)** |
| **Ghana** | **3153.62(2744.53-3518.58)** | **106.62(90.66-124.01)** | **366.3(281.8-462.95)** | **88.72(55.29-139.03)** |
| **Greece** | **1033.72(907.06-1148.93)** | **1836.28(1457.92-2290.91)** | **30219.92(24351.58-37184.33)** | **1077.25(906.59-1241.47)** |
| **Greenland** | **5.62(5.15-6.08)** | **4.42(3.85-5.04)** | **29.71(24.39-35.56)** | **2.1(1.27-2.95)** |
| **Grenada** | **10.32(9.07-11.55)** | **2.55(2.09-3.06)** | **25.89(20.44-31.95)** | **1.16(0.96-1.5)** |
| **Guam** | **17.06(14.91-19.15)** | **26.89(21.36-32.95)** | **359.86(282.49-446.02)** | **2.76(2.21-3.33)** |
| **Guatemala** | **1777.65(1465.21-2092.6)** | **82.35(68.31-97.8)** | **761.49(588.96-937.74)** | **34.89(26.94-44.32)** |
| **Guinea** | **1264.31(1136.59-1395.19)** | **35.02(30.6-40.1)** | **81.21(63.4-102.89)** | **33.82(19.63-53.25)** |
| **Guinea-Bissau** | **190.12(166.63-214.62)** | **5.19(4.49-5.93)** | **11.05(8.56-14.12)** | **5.33(2.66-9.19)** |
| **Guyana** | **77.07(68.38-85.71)** | **23.5(19.56-26.81)** | **194.93(155.62-238.46)** | **11.15(8.49-14.57)** |
| **Haiti** | **1240.21(1037.36-1471.34)** | **101.86(79.19-126.59)** | **552.07(417.62-701.04)** | **73.19(41.74-113.98)** |
| **Honduras** | **981.44(882.3-1083.34)** | **60.08(51.45-68.94)** | **570.47(458.89-696.93)** | **43.66(34.56-54.65)** |
| **Hungary** | **967.44(851.55-1078.9)** | **8293.27(7100.73-9884.45)** | **184447.06(159113.67-218514.62)** | **819.55(640.56-996.56)** |
| **Iceland** | **34.49(31.69-37.32)** | **59.12(47.81-72.1)** | **861.05(703.61-1044.49)** | **25.31(19.89-29.12)** |
| **India** | **139070.7(123777.34-155877.17)** | **9878.99(8321.07-11759.5)** | **23315.01(18285.71-29291.82)** | **6861.86(5180.92-8944.67)** |
| **Indonesia** | **25946.58(22684.34-29199.79)** | **1013.51(819.53-1261.67)** | **6015.23(4543.36-7786.62)** | **539.98(361.66-748.11)** |
| **Iran (Islamic Republic of)** | **8429.79(7733.06-9193.59)** | **466.3(385.19-564.74)** | **3463.69(2696.79-4337.22)** | **629.01(563.85-761.1)** |
| **Iraq** | **4211.95(3142.93-5298.19)** | **252.01(202.26-313.87)** | **1491.58(1114-1948.71)** | **78.96(54.08-104.37)** |
| **Ireland** | **491.04(448.38-535.59)** | **1031.37(762.69-1243.31)** | **14640.12(11042.03-17824.91)** | **238.6(199.8-272.21)** |
| **Israel** | **930.96(816.47-1055.09)** | **378.97(303.41-471.45)** | **4365.9(3423.19-5539.25)** | **488.43(395.87-555.36)** |
| **Italy** | **6031.32(5535.61-6498.39)** | **30306.88(25378.47-35943.09)** | **560971.32(474687.66-662132.23)** | **4375.67(3436.01-4957.02)** |
| **Jamaica** | **281.08(248.26-313.2)** | **45.59(36.27-61.03)** | **604.09(466-802.95)** | **9.84(7.54-13.05)** |
| **Japan** | **12778.84(11577.41-13987.85)** | **71837.44(58159.53-87203.45)** | **1411873.99(1186417.96-1700724.88)** | **12868.42(8511.89-15732.07)** |
| **Jordan** | **1163.67(1058.83-1267.89)** | **165.68(130.51-205.77)** | **1509.87(1149.82-1917.41)** | **27.28(22.15-33.56)** |
| **Kazakhstan** | **1839.21(1679.41-1992.16)** | **1061.61(847.72-1291.64)** | **17880.06(14237.52-21613.07)** | **33.32(26.66-43.21)** |
| **Kenya** | **5022.77(4365.11-5675.12)** | **219.53(183.08-263.21)** | **708.75(536.59-898.05)** | **151(116.23-195.97)** |
| **Kiribati** | **11.86(10.74-12.89)** | **0.73(0.59-0.9)** | **4.15(3.12-5.39)** | **0.4(0.28-0.62)** |
| **Kuwait** | **442.66(392.65-492.95)** | **271.88(210.92-340.11)** | **3192.62(2487.57-4024.46)** | **20.71(16.78-24.89)** |
| **Kyrgyzstan** | **653.55(569.78-731.52)** | **43.69(34.97-52.94)** | **603.65(470.84-738.45)** | **7.82(6.27-9.54)** |
| **Lao People's Democratic Republic** | **715.82(646.96-782.61)** | **12.94(10.61-15.78)** | **47.88(35.1-65.82)** | **9.83(7.1-14.4)** |
| **Latvia** | **191.53(176.02-207.14)** | **925.28(740.85-1152.39)** | **19331.09(15856.73-23481.41)** | **42.33(33.94-51.33)** |
| **Lebanon** | **517.71(445.53-592.85)** | **63.42(47.77-84.8)** | **588.48(437.06-782.75)** | **57.68(38.63-84.35)** |
| **Lesotho** | **209.16(191.03-227.33)** | **12.17(10.67-13.87)** | **44.54(35.08-56.15)** | **9.01(5.73-14.38)** |
| **Liberia** | **478.99(413.1-542.09)** | **15.85(12.99-19.16)** | **58.15(43.11-76.17)** | **11.42(6.72-17.85)** |
| **Libya** | **673.55(570.54-767.08)** | **90.83(72.25-113.56)** | **645.65(489.39-827.66)** | **38.13(22.89-53.94)** |
| **Lithuania** | **279.42(257.48-302.6)** | **1001.79(815.03-1213.44)** | **20323.89(16802.92-24467.58)** | **45.2(35.41-55.36)** |
| **Luxembourg** | **61.86(56.81-66.64)** | **173.43(138.66-213.08)** | **2621.11(2132.76-3210.05)** | **55.99(44.51-66.65)** |
| **Madagascar** | **2669.03(2037.38-3284.43)** | **94.77(84.53-106.62)** | **187.74(149.18-234.57)** | **104.8(69.41-147.76)** |
| **Malawi** | **1844.22(1714.98-1974.52)** | **51.45(44.61-59.09)** | **94.51(70.85-122.71)** | **54(39.47-73.15)** |
| **Malaysia** | **3130.14(2733.93-3519.05)** | **315.26(263.76-376.01)** | **2872.11(2240.33-3645.64)** | **119.72(92.97-154.59)** |
| **Maldives** | **49.84(44.85-54.64)** | **2.64(2.04-3.27)** | **19.26(14.81-24.64)** | **1.71(1.36-2.16)** |
| **Mali** | **2191.75(1912.61-2486.84)** | **43.71(37.24-51.41)** | **115.23(89.04-146.46)** | **45.01(25.86-76.46)** |
| **Malta** | **43.92(38.92-48.96)** | **68.15(57.67-80.78)** | **957.68(806.37-1156)** | **19.16(15.58-22.29)** |
| **Marshall Islands** | **5.68(4.98-6.34)** | **0.43(0.36-0.53)** | **2.57(1.99-3.24)** | **0.27(0.17-0.42)** |
| **Mauritania** | **401.43(356.19-444.2)** | **16.6(13.81-19.77)** | **79.53(60.72-100.81)** | **10(7.05-14.41)** |
| **Mauritius** | **127.67(111.37-144.29)** | **24.64(20.27-29.94)** | **252.47(194.87-325.82)** | **8.91(7.21-11.2)** |
| **Mexico** | **12494.02(10860.71-14063.03)** | **3502.89(2905.68-4101.12)** | **50230.15(40976.03-60266.02)** | **823.46(686.02-980.26)** |
| **Micronesia (Federated States of)** | **10.21(8.98-11.44)** | **1.92(1.56-2.38)** | **14.35(11.06-18.31)** | **0.59(0.35-0.92)** |
| **Monaco** | **3.76(3.43-4.08)** | **13.76(11.1-17.12)** | **266.03(219.65-329.05)** | **1.24(0.91-1.62)** |
| **Mongolia** | **338.76(297.75-379.54)** | **25.34(20.74-30.62)** | **311.91(245.61-387.69)** | **6.35(4.4-10.76)** |
| **Montenegro** | **62.03(54.59-69.56)** | **201.57(159.71-245.46)** | **4187.44(3401.7-5087.17)** | **4.57(3.7-5.67)** |
| **Morocco** | **3595.22(3233.87-3942.74)** | **213.94(177.96-259.32)** | **1124.52(868.78-1412.75)** | **283.33(196.16-376.27)** |
| **Mozambique** | **2952.8(2705.79-3180.83)** | **88.84(79.34-99.81)** | **147.97(115.79-187.11)** | **96.93(67.04-131.18)** |
| **Myanmar** | **5467.69(4890.78-6023.63)** | **129.27(103.6-159.02)** | **553.76(398.81-756.33)** | **102.09(78.04-143.57)** |
| **Namibia** | **240.31(211.27-268.51)** | **12.42(10.97-14.03)** | **43.12(34.19-52.51)** | **10.58(7.88-14.41)** |
| **Nauru** | **1.06(0.93-1.18)** | **0.08(0.07-0.1)** | **0.56(0.44-0.72)** | **0.04(0.02-0.06)** |
| **Nepal** | **3041.64(2661.14-3423.88)** | **174.35(152.5-200.42)** | **497.06(381.93-625.63)** | **158.92(110.61-220.55)** |
| **Netherlands** | **1715.68(1567.52-1861.33)** | **976.83(790.13-1199.83)** | **10494(8326.54-13411.48)** | **2053.9(1613.79-2411.65)** |
| **New Zealand** | **449.57(400.55-496.81)** | **2513.3(2095.54-2998.78)** | **32327.95(27212.44-38142.77)** | **430.74(346.36-489.52)** |
| **Nicaragua** | **651.04(551.44-757.43)** | **41.25(33.57-50.41)** | **512.04(402.51-632.37)** | **15.68(12.54-20.28)** |
| **Niger** | **2329.54(2079.77-2593.16)** | **37.28(31.46-44.71)** | **96.44(72.74-125.09)** | **38.54(21.87-63.09)** |
| **Nigeria** | **21482.38(19313.25-23657.36)** | **480.55(399.8-583.73)** | **1762.22(1344.56-2233.3)** | **427.09(292.25-616.67)** |
| **Niue** | **0.17(0.15-0.19)** | **0.09(0.07-0.11)** | **1.07(0.84-1.33)** | **0.02(0.01-0.02)** |
| **North Macedonia** | **215.27(178.55-252.76)** | **511.23(421.12-622.75)** | **9897.65(8139.41-11956.66)** | **19.28(14.91-24.67)** |
| **Northern Mariana Islands** | **4.25(3.72-4.79)** | **6.49(5.06-8.11)** | **64.53(48.87-81.74)** | **0.85(0.68-1.02)** |
| **Norway** | **534.88(493.67-575.48)** | **485.67(383.5-615.91)** | **5517.47(4328.9-7065.83)** | **691.2(548.31-784.6)** |
| **Oman** | **458.4(420.96-495.23)** | **40.97(32.21-51.11)** | **320.17(237.3-413.5)** | **20.92(13.63-29.32)** |
| **Pakistan** | **22406.28(20707.73-24165.74)** | **1336.11(1128.72-1570.79)** | **4105.9(3215.7-5109.79)** | **790.81(510.68-1071.57)** |
| **Palau** | **1.8(1.63-1.96)** | **1.1(0.87-1.38)** | **11.5(8.87-14.46)** | **0.05(0.04-0.06)** |
| **Palestine** | **495.66(456.18-532.97)** | **15.89(11.98-20.52)** | **96.39(70.33-127.67)** | **7.29(5.95-9.26)** |
| **Panama** | **416.05(365.93-467.92)** | **48.38(41.66-56.11)** | **635(517.46-772.47)** | **20.45(15.57-26.84)** |
| **Papua New Guinea** | **986.66(868.82-1095.24)** | **45.97(38.21-55.65)** | **241.03(186.43-300.26)** | **26.08(15.26-40.54)** |
| **Paraguay** | **693.05(569.92-811.1)** | **188.49(170.9-207.3)** | **1447.74(1202.96-1725.02)** | **115.65(86.37-148.6)** |
| **Peru** | **3399.54(3112.01-3662.62)** | **739.98(611.24-886.55)** | **11561.59(9339.29-14012.79)** | **96.9(70.05-131.49)** |
| **Philippines** | **11214.28(10158.09-12186.88)** | **249.98(198.5-315.98)** | **1364.17(996.99-1820.8)** | **136.13(110.83-165.76)** |
| **Poland** | **3843.44(3537.9-4136.49)** | **12398.84(10113.6-14847.98)** | **258294.1(209260.22-305719.67)** | **1522.06(1121.8-1931.62)** |
| **Portugal** | **1065.13(943.32-1190.9)** | **882.27(701.52-1090.87)** | **11252.44(9002.53-14286.93)** | **954.84(778.15-1081.4)** |
| **Puerto Rico** | **352.14(310.69-398.6)** | **1131.86(915.92-1369.87)** | **28773.38(22813.31-35815.28)** | **93.59(71.1-118.63)** |
| **Qatar** | **286.45(259.54-312.37)** | **251.74(195.85-313.6)** | **2668.05(2054.81-3338.88)** | **4.6(3.18-6.61)** |
| **Republic of Korea** | **5339.83(4844.1-5840.71)** | **14718.91(12134.8-17671.54)** | **268834.29(226639.75-317267.62)** | **664.47(536.44-786.9)** |
| **Republic of Moldova** | **368.82(309.57-432.74)** | **841.62(653.83-1055.2)** | **15713.66(12437.15-19127.86)** | **9.2(7.24-11.7)** |
| **Romania** | **1923.71(1703.01-2154.25)** | **15734.3(12568.64-19709.52)** | **369312.63(300495.81-454223.07)** | **298.74(236.3-364.34)** |
| **Russian Federation** | **14671.74(12885.02-16517.18)** | **54151.84(43433.2-65578.91)** | **1049883.7(839793.39-1270533.85)** | **1248.79(1015.32-1503.04)** |
| **Rwanda** | **1268.81(1134.43-1407.62)** | **40.82(35.28-47.33)** | **79.37(59.96-101.08)** | **47.99(34.15-68.99)** |
| **Saint Kitts and Nevis** | **5.95(4.8-7.09)** | **2.25(1.91-2.66)** | **27.81(22.28-33.41)** | **0.59(0.48-0.75)** |
| **Saint Lucia** | **17.46(15.36-19.5)** | **6.14(5.39-7.04)** | **70.94(58.58-85.79)** | **1.93(1.59-2.33)** |
| **Saint Vincent and the Grenadines** | **11.31(10.12-12.56)** | **3.68(3.22-4.24)** | **41.59(34.22-50.19)** | **1.39(1.16-1.72)** |
| **Samoa** | **21.14(19.3-22.93)** | **3.92(3.24-4.78)** | **36.25(28.54-45.46)** | **1.12(0.83-1.53)** |
| **San Marino** | **3.31(2.89-3.72)** | **6.56(5.11-7.85)** | **109.42(85.33-131.74)** | **2.04(1.32-2.91)** |
| **Sao Tome and Principe** | **20.54(18.24-22.91)** | **0.79(0.66-0.92)** | **3.63(2.74-4.52)** | **0.62(0.43-0.82)** |
| **Saudi Arabia** | **3573.2(3117.57-4019.2)** | **862.72(668.29-1076.3)** | **7647.25(5831.24-9605.12)** | **97.53(73.92-132.65)** |
| **Senegal** | **1513.41(1350.4-1685.25)** | **39.21(33.16-46.46)** | **119.68(92.06-152.01)** | **37.86(25.47-53.56)** |
| **Serbia** | **874.68(782.98-973.06)** | **3706.98(3131.25-4426.46)** | **78339.46(66344.62-92902.76)** | **443.34(339.04-574.46)** |
| **Seychelles** | **10.21(8.95-11.45)** | **2.2(1.68-2.89)** | **22.84(16.94-29.81)** | **0.68(0.54-0.83)** |
| **Sierra Leone** | **828.48(752.63-908.02)** | **21.52(18.44-25.03)** | **57.44(44.46-74.02)** | **20.04(12.04-30.74)** |
| **Singapore** | **566.75(523.31-605.87)** | **1976.18(1606.22-2391.68)** | **34091.97(28074.26-40795.77)** | **35.16(27.17-42.09)** |
| **Slovakia** | **543.72(496.99-592.36)** | **1824.48(1541.47-2171.36)** | **36995.22(30865.34-43898.52)** | **135.1(101.43-172.4)** |
| **Slovenia** | **207.43(191.44-224.32)** | **2114.86(1517.01-2755.28)** | **44250.92(33263.15-56436.76)** | **363.7(262.3-482.96)** |
| **Solomon Islands** | **65.56(57.07-74.26)** | **3.37(2.77-4.06)** | **18.92(14.75-23.82)** | **2.26(1.18-3.82)** |
| **Somalia** | **2034.31(1520.14-2570.36)** | **34.95(30.09-40.88)** | **52.55(39.93-69.57)** | **41.97(24.23-67.01)** |
| **South Africa** | **5558.84(4916.97-6272.47)** | **4529.28(3612.05-5584.28)** | **56993.16(43896.39-72883.15)** | **348.55(283.57-394.58)** |
| **South Sudan** | **928.3(805.08-1061.35)** | **26.12(22.24-30.59)** | **53.28(39.7-70.07)** | **19.82(13.1-28.8)** |
| **Spain** | **4602.12(4208.8-4998.15)** | **5645.53(4544.73-6886.05)** | **82883.96(66763.67-102701.56)** | **5153.06(4177-6116.47)** |
| **Sri Lanka** | **2185.45(1944.51-2414)** | **154.13(129.68-181.31)** | **1070.27(818.46-1354.2)** | **151.83(110.77-205.59)** |
| **Sudan** | **4080.84(3535.61-4600.97)** | **124.09(104.37-147.06)** | **508.25(390.38-646.09)** | **162.98(107.71-242.36)** |
| **Suriname** | **57.59(51.22-64.86)** | **15.17(13.15-17.54)** | **169.77(136.83-206.5)** | **4.78(3.86-5.94)** |
| **Sweden** | **1022.25(931.23-1112.75)** | **3764.91(3049.82-4642.64)** | **60974.82(49804.38-74278.39)** | **1209.17(979.36-1405.81)** |
| **Switzerland** | **877.52(802.17-956.46)** | **2729.98(2309.89-3178.81)** | **43793.56(36987.37-51576.63)** | **746.29(581.12-880.27)** |
| **Syrian Arab Republic** | **1449.12(1217.37-1679.56)** | **128.11(111.4-146.21)** | **759.71(593.83-946.67)** | **186.43(129.15-268.47)** |
| **Taiwan (Province of China)** | **2362.02(2165.86-2544.32)** | **1560.15(1302.8-1885.7)** | **21911.17(18162.23-26764.15)** | **370.2(279.7-471.91)** |
| **Tajikistan** | **949.24(821.39-1067.48)** | **29.84(23.79-36.98)** | **353.38(272.98-460.78)** | **9.76(7.74-12.19)** |
| **Thailand** | **7011.16(6132.9-7890.95)** | **1050.08(886.22-1252.09)** | **9793.35(7633.48-12302.78)** | **360.43(264.09-472.85)** |
| **Timor-Leste** | **133.48(120.88-144.74)** | **2.28(1.87-2.77)** | **9.77(7.19-13.11)** | **1.7(1.11-2.59)** |
| **Togo** | **792.15(694.3-889.79)** | **25.19(21.93-28.97)** | **69.66(54.01-88.02)** | **22.85(13.38-37.01)** |
| **Tokelau** | **0.14(0.13-0.15)** | **0.03(0.02-0.03)** | **0.24(0.19-0.31)** | **0.01(0.01-0.01)** |
| **Tonga** | **10.24(9.36-11.12)** | **2.56(2.11-3.09)** | **27.28(21.71-33.86)** | **0.59(0.43-0.8)** |
| **Trinidad and Tobago** | **138.75(122.63-154.94)** | **57.7(46.68-71.38)** | **825.74(656.35-1019.48)** | **10.41(7.85-13.61)** |
| **Tunisia** | **1157.16(1042.37-1275.79)** | **106.3(88.57-128.55)** | **847.5(664.22-1070.55)** | **107.99(69.15-153.7)** |
| **Turkey** | **8135.97(7136.61-9123.67)** | **1545.85(1389.78-1730.6)** | **11166.87(8896.99-13574.35)** | **851.22(632.49-1092.68)** |
| **Turkmenistan** | **508.31(461.4-554.49)** | **124.03(99.95-150.01)** | **1858.73(1475.43-2276.77)** | **7.02(5.37-9.26)** |
| **Tuvalu** | **1.18(1.05-1.32)** | **0.12(0.1-0.14)** | **0.92(0.7-1.16)** | **0.08(0.06-0.11)** |
| **Uganda** | **4111.79(3702.37-4495.55)** | **89.78(78.22-104.49)** | **165.75(126.62-213)** | **101.55(74.99-138.75)** |
| **Ukraine** | **4404.24(3574.55-5226.8)** | **8632.81(6820.62-10662.16)** | **168865.68(133883.47-206428.54)** | **185.21(154.47-221.29)** |
| **United Arab Emirates** | **924.17(776.46-1059.27)** | **625.63(467.92-818.87)** | **5738.16(4365.13-7301.28)** | **51.58(26.75-79.78)** |
| **United Kingdom** | **6722.04(6046.87-7392.54)** | **29659.88(24842.48-35286.42)** | **431375.62(360324.87-516764.55)** | **5408.66(4572.07-5893.47)** |
| **United Republic of Tanzania** | **5673.61(5049.55-6323.04)** | **190.42(165.95-220.43)** | **409.41(310.76-524.15)** | **213.95(146.12-303.32)** |
| **United States of America** | **32797.87(28595.93-36932.42)** | **117079.89(100604.05-135189.82)** | **1425072.93(1243341.34-1649908.1)** | **24825.95(20353.87-27717.51)** |
| **United States Virgin Islands** | **10.4(9.12-11.67)** | **23.97(19.74-28.65)** | **484.32(387.75-594.92)** | **3.48(2.87-4.18)** |
| **Uruguay** | **343.61(303.12-387.7)** | **698.52(616.12-793.66)** | **7000.64(5879.22-8385.87)** | **342.85(286.93-391.79)** |
| **Uzbekistan** | **3367.71(2541.1-4231.94)** | **627.92(509.75-745.22)** | **7733.27(6101.91-9524.8)** | **67.74(49.95-95.55)** |
| **Vanuatu** | **29.46(26.8-32.06)** | **2.31(1.92-2.77)** | **14.06(10.98-17.79)** | **1.28(0.83-1.87)** |
| **Venezuela (Bolivarian Republic of)** | **2806.9(2476.94-3142.38)** | **534.37(466.56-611.16)** | **6324.83(5184.92-7603.21)** | **270.12(201.86-364.14)** |
| **Viet Nam** | **9637.29(8306.64-10900.7)** | **301.88(248.08-367.25)** | **1795.72(1332.69-2367.95)** | **244.66(178.52-340.87)** |
| **Yemen** | **3150.29(2659.69-3677.51)** | **81.69(70.29-95.55)** | **268.12(207.88-346.59)** | **118.51(80.2-167.54)** |
| **Zambia** | **1823.77(1588.59-2047.33)** | **48.63(42.1-56.43)** | **102.89(77.94-133.08)** | **66.6(49.01-89.89)** |
| **Zimbabwe** | **1501.09(1331.72-1665.07)** | **57.3(50.59-65.08)** | **172.4(137.63-210)** | **48.54(35.26-68.86)** |
| **Abbreviations: CAVD, calcific aortic valve disease; UI, uncertainty interval.** | | | | |
