# Supplementary material for: Changing epidemiology of calcific aortic valve disease: 30-year trends of incidence, prevalence, and deaths across 204 countries and territories
Source: Aging (Albany NY). 2021 May 11;13(9):12710–32. doi: 10.18632/aging.202942 (PMC8148466; doi:10.18632/aging.202942)
Supplement: Supplementary Table 3 [file aging-13-202942-s004.docx]

**Supplementary Table 3. The change of CAVD prevalence between 1990 and 2019 and EAPC at 204 countries and territories.**

|  |  | **All-ages Prevalence** | | |  |  | **ASPR** | | |  |
| --- | --- | --- | --- | --- | --- | --- | --- | --- | --- | --- |
| **Location** | **1990 No. (95% UI)** | | **2019 No. (95% UI)** | **Change in Absolute Number (95% UI)** | | **1990 per 100,000 No. (95% UI)** | | **2019 per 100,000 No. (95% UI)** | **EAPC No. (95% CI)** | |
| **Afghanistan** | **60.58(45.82-79.48)** | | **170.37(124.87-228.24)** | **1.81(1.35-2.36)** | | **0.78(0.6-1.02)** | | **1.04(0.8-1.34)** | **0.98(-8.74-11.73)** | |
| **Albania** | **654.36(542.52-782.08)** | | **10141.92(8376.99-12306.05)** | **14.5(12.28-16.94)** | | **31.31(26.06-37.57)** | | **239.18(199.2-289.35)** | **7.26(5.89-8.65)** | |
| **Algeria** | **403.24(311.18-503.34)** | | **1728.94(1336.93-2185.01)** | **3.29(2.81-3.91)** | | **2.92(2.28-3.66)** | | **4.64(3.63-5.84)** | **1.61(-3.39-6.87)** | |
| **American Samoa** | **9.11(7.14-11.43)** | | **91.13(71.72-114.05)** | **9(7.24-11.42)** | | **45.2(35.49-57.11)** | | **195.91(153.9-245.53)** | **5.19(4.02-6.37)** | |
| **Andorra** | **4.04(3.14-5.17)** | | **237.12(179.76-294.75)** | **57.72(43.55-73.76)** | | **7.18(5.66-9.14)** | | **171.77(130.03-213.02)** | **11.57(8.73-14.48)** | |
| **Angola** | **39.54(30.49-51.06)** | | **148.99(114.56-189.88)** | **2.77(2.37-3.23)** | | **0.91(0.72-1.13)** | | **1.2(0.95-1.5)** | **0.96(-8.09-10.9)** | |
| **Antigua and Barbuda** | **3.33(2.75-3.98)** | | **31.02(25.53-36.82)** | **8.32(6.91-9.91)** | | **6.4(5.23-7.74)** | | **29.65(24.38-35.08)** | **5.43(2.37-8.58)** | |
| **Argentina** | **3538.53(2751.08-4663.87)** | | **55366.98(47035.94-66507.15)** | **14.65(11.34-18.47)** | | **10.96(8.53-14.45)** | | **102.43(86.97-122.81)** | **8.01(5.72-10.36)** | |
| **Armenia** | **141.52(108.17-181.86)** | | **2287.98(1858.52-2807.21)** | **15.17(11.89-18.88)** | | **5.05(3.89-6.4)** | | **55.18(44.9-67.49)** | **8.6(5.24-12.06)** | |
| **Australia** | **6564.98(5425.09-7893.21)** | | **288496.62(243984.57-345487.19)** | **42.94(35.92-51.27)** | | **33.21(27.6-39.89)** | | **695.19(590.19-832.23)** | **11.06(9.73-12.4)** | |
| **Austria** | **6098.71(5183.62-7207.33)** | | **121170.14(104957.54-137387.47)** | **18.87(16.07-22.63)** | | **52.03(44.12-61.23)** | | **668.14(576.67-759.72)** | **9.2(8.14-10.27)** | |
| **Azerbaijan** | **249.02(185.69-328.05)** | | **4647.19(3661.18-5738.56)** | **17.66(13.72-22.45)** | | **4.86(3.63-6.28)** | | **45.27(36.12-55.52)** | **8(4.57-11.54)** | |
| **Bahamas** | **14.35(11.69-17.17)** | | **156.2(127.86-186.95)** | **9.89(8.49-11.5)** | | **8.71(7.08-10.42)** | | **38.24(31.29-45.62)** | **5.23(2.6-7.94)** | |
| **Bahrain** | **52.22(39.49-66.04)** | | **603.32(456.76-768.22)** | **10.55(9.15-12.3)** | | **19.08(14.93-23.99)** | | **40.24(31.18-50.8)** | **2.61(0.7-4.55)** | |
| **Bangladesh** | **565.35(433.92-730.85)** | | **2240.38(1745.33-2814.81)** | **2.96(2.53-3.49)** | | **1.09(0.85-1.4)** | | **1.64(1.28-2.04)** | **1.41(-6.72-10.24)** | |
| **Barbados** | **34.83(28.84-41.41)** | | **276.15(222.44-331.96)** | **6.93(5.76-8.24)** | | **12.59(10.35-15)** | | **57.18(46.41-68.28)** | **5.36(3.16-7.6)** | |
| **Belarus** | **3026.18(2368.09-3726.06)** | | **35853.75(28627.66-44269.13)** | **10.85(9.29-12.7)** | | **23.35(18.4-28.59)** | | **229.73(183.94-280.39)** | **8.2(6.63-9.8)** | |
| **Belgium** | **517.39(406.55-665.5)** | | **8835.75(6943.98-11221.16)** | **16.08(13.11-20.45)** | | **3.35(2.62-4.33)** | | **39(30.82-48.99)** | **8.83(4.72-13.1)** | |
| **Belize** | **5.02(4-6.13)** | | **124.89(99.65-159.65)** | **23.86(19.69-30.77)** | | **5.17(4.13-6.34)** | | **42.7(33.9-55.26)** | **7.55(4.22-10.99)** | |
| **Benin** | **24.27(18.99-30.53)** | | **98.98(75.61-128.34)** | **3.08(2.58-3.66)** | | **1.08(0.85-1.34)** | | **1.65(1.27-2.08)** | **1.47(-6.68-10.34)** | |
| **Bermuda** | **34.3(27.35-41.93)** | | **347.25(291-414.43)** | **9.12(7.33-11.48)** | | **53.42(42.76-65.48)** | | **274.8(231.59-328.97)** | **5.81(4.75-6.88)** | |
| **Bhutan** | **4.43(3.35-5.83)** | | **29.64(21.72-39.88)** | **5.69(4.52-7.77)** | | **1.58(1.22-2.03)** | | **4.95(3.67-6.65)** | **4.01(-2.21-10.64)** | |
| **Bolivia (Plurinational State of)** | **87.33(70.1-108.07)** | | **1442.21(1184.09-1734.73)** | **15.52(13.14-18.42)** | | **2.49(2-3.06)** | | **15.7(12.98-19.01)** | **6.56(1.76-11.59)** | |
| **Bosnia and Herzegovina** | **1612.72(1300.25-1965.27)** | | **17469.25(14381.32-21149.95)** | **9.83(8.44-11.78)** | | **40.23(32.33-48.85)** | | **294.68(245.88-354.85)** | **7.11(5.9-8.33)** | |
| **Botswana** | **9.64(7.53-12.08)** | | **90.07(68.43-114.02)** | **8.35(6.79-10.23)** | | **1.58(1.26-1.96)** | | **5.98(4.64-7.49)** | **4.69(-1.45-11.22)** | |
| **Brazil** | **7905.37(6332.97-9659.23)** | | **57152.9(45926.57-70348.39)** | **6.23(5.6-7.01)** | | **7.85(6.26-9.62)** | | **23.7(19.11-29)** | **3.88(1.03-6.81)** | |
| **Brunei Darussalam** | **66.76(53.09-82.71)** | | **899.49(747.64-1088.82)** | **12.47(10.68-14.84)** | | **62.64(48.9-79.56)** | | **289.41(246.36-342.68)** | **5.42(4.43-6.42)** | |
| **Bulgaria** | **15625.97(12391-19290.29)** | | **48313.6(39016.85-58507.8)** | **2.09(1.73-2.48)** | | **124.24(98.96-152.51)** | | **357.88(289.22-429.53)** | **3.72(2.99-4.45)** | |
| **Burkina Faso** | **44.31(34.68-56.13)** | | **153.5(118.91-195.88)** | **2.46(2.07-2.93)** | | **0.89(0.7-1.13)** | | **1.39(1.08-1.74)** | **1.55(-7.36-11.31)** | |
| **Burundi** | **21.41(16.89-26.58)** | | **40.75(30.5-53.04)** | **0.9(0.65-1.17)** | | **0.83(0.67-1.03)** | | **0.78(0.61-0.98)** | **-0.23(-10.32-10.98)** | |
| **Cabo Verde** | **3.76(2.95-4.63)** | | **16.3(12.78-20.31)** | **3.33(2.83-3.95)** | | **1.74(1.36-2.13)** | | **3.42(2.71-4.24)** | **2.37(-3.88-9.02)** | |
| **Cambodia** | **16.75(12.31-22.56)** | | **137.41(99.61-188.34)** | **7.2(5.89-8.96)** | | **0.37(0.28-0.5)** | | **1.15(0.85-1.53)** | **3.95(-8.46-18.05)** | |
| **Cameroon** | **111.03(84.16-141.41)** | | **450.61(344.02-577.48)** | **3.06(2.53-3.73)** | | **2.13(1.64-2.72)** | | **3.05(2.37-3.86)** | **1.24(-4.68-7.54)** | |
| **Canada** | **1908.95(1505.65-2399.45)** | | **67764.69(56039.81-80665.54)** | **34.5(27.87-42.61)** | | **5.86(4.64-7.34)** | | **100.8(83.69-119.91)** | **10.31(7.18-13.52)** | |
| **Central African Republic** | **9.51(7.41-12.03)** | | **19.47(15.18-24.25)** | **1.05(0.84-1.28)** | | **0.76(0.6-0.94)** | | **0.82(0.65-1.01)** | **0.25(-9.97-11.63)** | |
| **Chad** | **24.26(18.88-30.85)** | | **69.68(53.88-89.53)** | **1.87(1.55-2.22)** | | **0.78(0.61-0.99)** | | **0.99(0.78-1.26)** | **0.82(-8.98-11.68)** | |
| **Chile** | **1464.05(1208.7-1765.07)** | | **39520.69(32889.04-47405.09)** | **25.99(22.28-30.87)** | | **14.25(11.79-17.1)** | | **166.28(138.23-198.59)** | **8.84(6.83-10.89)** | |
| **China** | **17791.1(13302.48-23691.17)** | | **867916.68(687947.96-1064921.33)** | **47.78(38.57-59.02)** | | **2.12(1.58-2.76)** | | **42.75(34.03-52.12)** | **10.92(5.77-16.33)** | |
| **Colombia** | **1304.78(1010.31-1602.52)** | | **14538.68(11904.3-17575.66)** | **10.14(8.21-12.53)** | | **7.02(5.43-8.66)** | | **27.56(22.58-33.42)** | **4.83(1.88-7.87)** | |
| **Comoros** | **3.08(2.43-3.86)** | | **7.65(5.9-9.76)** | **1.49(1.2-1.81)** | | **1.3(1.02-1.61)** | | **1.47(1.14-1.85)** | **0.43(-7.43-8.95)** | |
| **Congo** | **16.04(12.57-20.26)** | | **49.37(37.81-62.64)** | **2.08(1.74-2.49)** | | **1.42(1.13-1.77)** | | **1.77(1.37-2.22)** | **0.76(-6.63-8.73)** | |
| **Cook Islands** | **1.29(1.02-1.64)** | | **29.89(23.04-36.94)** | **22.19(18.09-27.56)** | | **10.9(8.57-13.69)** | | **118.44(91.5-146.01)** | **8.57(6.28-10.92)** | |
| **Costa Rica** | **193.28(160.31-232.83)** | | **2541.53(2115.02-3037.08)** | **12.15(10.28-14.33)** | | **10.84(8.98-13.04)** | | **48.79(40.59-58.17)** | **5.32(2.96-7.74)** | |
| **Croatia** | **4421.05(3569.95-5316.01)** | | **63553.55(54339.03-73922.25)** | **13.38(11.57-15.69)** | | **68.76(55.68-82.56)** | | **761.43(654.39-881.08)** | **8.65(7.72-9.57)** | |
| **Cuba** | **774.15(638.05-929.09)** | | **8981.54(7468.06-10721.56)** | **10.6(8.82-12.56)** | | **7.47(6.16-8.98)** | | **49.03(40.91-58.48)** | **6.7(3.91-9.57)** | |
| **Cyprus** | **42.14(32.59-53.37)** | | **2657(2136.06-3279.02)** | **62.05(50.78-77.03)** | | **4.93(3.85-6.17)** | | **131.73(106.46-162.28)** | **12(8.58-15.53)** | |
| **Czechia** | **8927.72(7022.05-11254.07)** | | **135355.52(112016.5-163024)** | **14.16(11.49-17.52)** | | **65.24(51.59-81.37)** | | **676.09(559.92-802)** | **8.4(7.45-9.35)** | |
| **Côte d'Ivoire** | **74.82(57.41-95.65)** | | **242.54(188.2-309.12)** | **2.24(1.85-2.7)** | | **1.37(1.06-1.71)** | | **1.72(1.35-2.16)** | **0.81(-6.7-8.92)** | |
| **Democratic People's Republic of Korea** | **143.91(106.22-193.41)** | | **1190.27(915.91-1530.54)** | **7.27(5.73-9.28)** | | **0.91(0.69-1.19)** | | **3.66(2.84-4.66)** | **4.93(-3.06-13.58)** | |
| **Democratic Republic of the Congo** | **167.58(129.57-216.67)** | | **444.97(342.2-575.93)** | **1.66(1.39-1.96)** | | **0.97(0.77-1.24)** | | **1.12(0.88-1.41)** | **0.5(-8.48-10.37)** | |
| **Denmark** | **394.99(304.1-510.76)** | | **16873.48(13725.97-20994.02)** | **41.72(34.38-50.6)** | | **4.83(3.71-6.18)** | | **142.9(116.33-175.93)** | **12.39(8.93-15.96)** | |
| **Djibouti** | **2.14(1.64-2.74)** | | **10.95(8.35-14.11)** | **4.11(3.53-4.86)** | | **1.22(0.97-1.52)** | | **1.55(1.22-1.94)** | **0.84(-7.09-9.44)** | |
| **Dominica** | **4.82(3.96-5.8)** | | **33.29(27.25-40.31)** | **5.9(4.87-7.2)** | | **7.06(5.81-8.49)** | | **37.31(30.66-44.91)** | **5.91(3.01-8.89)** | |
| **Dominican Republic** | **136.85(109.75-166.18)** | | **2205.61(1823.69-2649.79)** | **15.12(12.64-18.35)** | | **3.35(2.69-4.14)** | | **23.21(19.2-27.72)** | **6.9(2.76-11.2)** | |
| **Ecuador** | **366.38(291.55-453.56)** | | **20348.22(17505.14-23403.86)** | **54.54(44.88-65.74)** | | **6.48(5.17-8)** | | **132.9(114.58-153.07)** | **10.98(8-14.04)** | |
| **Egypt** | **2376.1(1757.58-3087.19)** | | **9705.76(7368.99-12609.96)** | **3.08(2.58-3.68)** | | **7.14(5.3-9.32)** | | **13.43(10.24-17.4)** | **2.2(-0.95-5.45)** | |
| **El Salvador** | **91.49(70.74-114.5)** | | **842.83(651.44-1101.43)** | **8.21(6.65-10.55)** | | **2.98(2.31-3.76)** | | **14.48(11.13-18.99)** | **5.6(1.16-10.24)** | |
| **Equatorial Guinea** | **1.75(1.36-2.22)** | | **20.83(15.57-26.94)** | **10.9(9.01-13.33)** | | **0.84(0.66-1.06)** | | **4.01(3.01-5.09)** | **5.55(-2.68-14.49)** | |
| **Eritrea** | **8.86(6.88-11.21)** | | **26.52(20.18-34.3)** | **1.99(1.61-2.39)** | | **0.75(0.6-0.91)** | | **0.86(0.68-1.07)** | **0.49(-9.7-11.83)** | |
| **Estonia** | **1051.53(853.96-1273.72)** | | **18715.11(14075.47-23511.91)** | **16.8(13.46-20.89)** | | **51.27(41.94-61.85)** | | **759.94(578.58-943.34)** | **9.74(8.68-10.82)** | |
| **Eswatini** | **11.29(7.93-15.56)** | | **65.62(44.87-89.49)** | **4.81(3.67-6.77)** | | **3.66(2.59-5.11)** | | **10.39(7.14-13.98)** | **3.66(-0.51-8.01)** | |
| **Ethiopia** | **156.3(118.38-203.38)** | | **512.23(387.25-657.25)** | **2.28(1.99-2.61)** | | **0.68(0.52-0.86)** | | **1.05(0.81-1.35)** | **1.53(-8.61-12.8)** | |
| **Fiji** | **12.42(9.79-15.89)** | | **158.01(123.96-199.49)** | **11.72(9.35-14.29)** | | **3.96(3.09-5.19)** | | **22.15(17.46-27.72)** | **6.12(2.28-10.11)** | |
| **Finland** | **3009.13(2470.62-3644.8)** | | **53982.03(40850.42-67091.72)** | **16.94(13.03-22.31)** | | **40.11(33.2-48.45)** | | **432.08(330.68-536.75)** | **8.54(7.34-9.76)** | |
| **France** | **4797.29(3788.62-6019.03)** | | **109064.62(86214.37-138503.2)** | **21.73(17.72-26.66)** | | **5.8(4.6-7.32)** | | **79.49(63.32-99.16)** | **9.45(6.31-12.68)** | |
| **Gabon** | **10.97(8.52-13.88)** | | **112.43(84.31-146.91)** | **9.25(7.36-11.59)** | | **1.9(1.49-2.38)** | | **10.01(7.57-12.9)** | **5.9(0.38-11.72)** | |
| **Gambia** | **5.33(4.16-6.87)** | | **18.2(14.16-23.08)** | **2.41(2-2.88)** | | **1.21(0.96-1.54)** | | **1.57(1.23-1.96)** | **0.9(-7.01-9.49)** | |
| **Georgia** | **1122.21(876.96-1373.59)** | | **5384.31(4500.68-6431.52)** | **3.8(3.11-4.74)** | | **17.93(14.11-21.82)** | | **92.12(76.88-110.29)** | **5.81(3.98-7.67)** | |
| **Germany** | **7099.14(5580.95-8884.81)** | | **287038.54(235176.32-348616.54)** | **39.43(31.97-47.65)** | | **5.71(4.5-7.16)** | | **147.9(121.44-179.61)** | **11.88(8.7-15.15)** | |
| **Ghana** | **72.55(55.14-94.93)** | | **366.3(281.8-462.95)** | **4.05(3.41-4.92)** | | **0.95(0.73-1.22)** | | **1.89(1.47-2.33)** | **2.4(-5.95-11.5)** | |
| **Greece** | **1001.84(779.1-1296.28)** | | **30219.92(24351.58-37184.33)** | **29.16(23.18-36.1)** | | **6.5(5.1-8.34)** | | **126.19(103.74-154.94)** | **10.77(7.8-13.82)** | |
| **Greenland** | **1.5(1.16-1.9)** | | **29.71(24.39-35.56)** | **18.8(14.73-23.73)** | | **3.79(3.02-4.71)** | | **45.34(37.49-54.11)** | **8.94(5.07-12.95)** | |
| **Grenada** | **3.2(2.31-3.97)** | | **25.89(20.44-31.95)** | **7.1(5.48-9.69)** | | **4.7(3.45-5.82)** | | **22.06(17.49-27.16)** | **5.47(1.91-9.16)** | |
| **Guam** | **17.76(14.27-21.81)** | | **359.86(282.49-446.02)** | **19.26(15.82-23.5)** | | **26.5(21.68-32.57)** | | **189.76(149.17-234.59)** | **7.02(5.53-8.53)** | |
| **Guatemala** | **80.07(60.58-103.14)** | | **761.49(588.96-937.74)** | **8.51(7.17-10.28)** | | **1.96(1.49-2.5)** | | **6.42(5.02-7.93)** | **4.18(-1.41-10.09)** | |
| **Guinea** | **31.5(24.41-40.58)** | | **81.21(63.4-102.89)** | **1.58(1.25-1.96)** | | **0.87(0.68-1.12)** | | **1.25(0.99-1.57)** | **1.25(-7.88-11.28)** | |
| **Guinea-Bissau** | **5.06(3.82-6.44)** | | **11.05(8.56-14.12)** | **1.18(0.94-1.55)** | | **1.04(0.8-1.31)** | | **1.16(0.92-1.45)** | **0.39(-8.36-9.97)** | |
| **Guyana** | **21.67(17.22-26.67)** | | **194.93(155.62-238.46)** | **8(6.49-10.15)** | | **5.08(4.06-6.25)** | | **29.2(23.48-35.73)** | **6.22(2.82-9.72)** | |
| **Haiti** | **89.2(69.29-108.68)** | | **552.07(417.62-701.04)** | **5.19(4.11-6.27)** | | **2.51(1.95-3.08)** | | **7.17(5.36-9.18)** | **3.69(-1.33-8.95)** | |
| **Honduras** | **60.43(47.18-74.52)** | | **570.47(458.89-696.93)** | **8.44(7.23-9.96)** | | **2.73(2.14-3.4)** | | **8.74(7.08-10.67)** | **4.09(-0.68-9.08)** | |
| **Hungary** | **27752.34(23433.17-32723.26)** | | **184447.06(159113.67-218514.62)** | **5.65(4.8-6.69)** | | **186.85(157.65-219.63)** | | **997.07(861.74-1167.68)** | **5.94(5.37-6.52)** | |
| **Iceland** | **18.21(14.28-23.16)** | | **861.05(703.61-1044.49)** | **46.3(38.06-57.38)** | | **6.38(5.01-8.12)** | | **155.92(128.08-189.36)** | **11.65(8.65-14.74)** | |
| **India** | **6161.75(4676.11-7872.63)** | | **23315.01(18285.71-29291.82)** | **2.78(2.54-3.04)** | | **1.21(0.93-1.51)** | | **1.94(1.52-2.42)** | **1.64(-6.02-9.93)** | |
| **Indonesia** | **558.23(405.28-747.78)** | | **6015.23(4543.36-7786.62)** | **9.78(8.36-11.47)** | | **0.55(0.41-0.73)** | | **2.72(2.09-3.5)** | **5.63(-4.38-16.69)** | |
| **Iran (Islamic Republic of)** | **1124.35(871.83-1427.63)** | | **3463.69(2696.79-4337.22)** | **2.08(1.87-2.32)** | | **3.54(2.74-4.47)** | | **4.18(3.26-5.23)** | **0.57(-4.22-5.61)** | |
| **Iraq** | **684.08(508.3-896.46)** | | **1491.58(1114-1948.71)** | **1.18(0.89-1.48)** | | **8.13(6.06-10.68)** | | **5.67(4.3-7.39)** | **-1.24(-4.82-2.49)** | |
| **Ireland** | **263.23(204.27-340.35)** | | **14640.12(11042.03-17824.91)** | **54.62(41.35-70.49)** | | **6.3(4.89-8.06)** | | **192.79(145.96-234.79)** | **12.52(9.48-15.64)** | |
| **Israel** | **191.33(145.17-245.92)** | | **4365.9(3423.19-5539.25)** | **21.82(17.44-27.28)** | | **3.87(2.95-4.91)** | | **37.58(29.51-47.6)** | **8.15(4.32-12.13)** | |
| **Italy** | **74111.15(60294.74-89699.08)** | | **560971.32(474687.66-662132.23)** | **6.57(5.84-7.44)** | | **84.07(68.6-101.04)** | | **408.26(347.04-476.32)** | **5.6(4.75-6.46)** | |
| **Jamaica** | **67.92(53.73-84.25)** | | **604.09(466-802.95)** | **7.89(6.25-10.13)** | | **3.89(3.07-4.86)** | | **20.35(15.71-27.14)** | **5.87(1.99-9.9)** | |
| **Japan** | **439043.09(357528.66-530646.65)** | | **1411873.99(1186417.96-1700724.88)** | **2.22(1.88-2.59)** | | **261.58(214.61-314.48)** | | **435.47(369.13-514.16)** | **1.77(1.24-2.31)** | |
| **Jordan** | **106.58(78.66-144.88)** | | **1509.87(1149.82-1917.41)** | **13.17(10.52-16.55)** | | **6.34(4.74-8.73)** | | **18.74(14.28-23.93)** | **3.81(0.63-7.08)** | |
| **Kazakhstan** | **1534.73(1176.31-1951.51)** | | **17880.06(14237.52-21613.07)** | **10.65(8.77-13.16)** | | **11.96(9.2-15.25)** | | **97.94(77.96-117.06)** | **7.52(5.32-9.77)** | |
| **Kenya** | **120.05(92.44-153.17)** | | **708.75(536.59-898.05)** | **4.9(4.17-5.75)** | | **1.32(1.03-1.7)** | | **2.77(2.12-3.52)** | **2.57(-4.5-10.17)** | |
| **Kiribati** | **0.68(0.54-0.89)** | | **4.15(3.12-5.39)** | **5.07(4.1-6.25)** | | **2.08(1.64-2.71)** | | **6.5(4.98-8.33)** | **4.01(-1.44-9.76)** | |
| **Kuwait** | **577.09(447.37-729.45)** | | **3192.62(2487.57-4024.46)** | **4.53(3.93-5.17)** | | **59.49(46.5-75.12)** | | **85.34(66.96-106.72)** | **1.25(0.1-2.41)** | |
| **Kyrgyzstan** | **115.88(88.03-150.68)** | | **603.65(470.84-738.45)** | **4.21(3.35-5.38)** | | **3.75(2.85-4.83)** | | **12.13(9.57-14.79)** | **4.13(0.06-8.37)** | |
| **Lao People's Democratic Republic** | **8.52(6.33-11.29)** | | **47.88(35.1-65.82)** | **4.62(3.76-5.62)** | | **0.41(0.31-0.53)** | | **1.11(0.82-1.49)** | **3.5(-8.55-17.14)** | |
| **Latvia** | **1889.84(1567.48-2295.1)** | | **19331.09(15856.73-23481.41)** | **9.23(7.92-11.07)** | | **53.05(44.04-63.93)** | | **526.67(435.44-631.71)** | **8.24(7.19-9.3)** | |
| **Lebanon** | **155.58(113.07-220.93)** | | **588.48(437.06-782.75)** | **2.78(2.28-3.45)** | | **6.17(4.49-8.69)** | | **11.26(8.34-15.04)** | **2.1(-1.3-5.61)** | |
| **Lesotho** | **13.18(10.37-16.54)** | | **44.54(35.08-56.15)** | **2.38(1.92-3.05)** | | **1.32(1.06-1.65)** | | **3.28(2.6-4.08)** | **3.18(-3.76-10.61)** | |
| **Liberia** | **13.88(10.54-17.76)** | | **58.15(43.11-76.17)** | **3.19(2.66-3.91)** | | **1.16(0.88-1.49)** | | **2.18(1.65-2.81)** | **2.19(-5.44-10.44)** | |
| **Libya** | **228.68(172.64-291.16)** | | **645.65(489.39-827.66)** | **1.82(1.51-2.16)** | | **10.87(8.23-13.9)** | | **11.24(8.49-14.42)** | **0.12(-2.72-3.03)** | |
| **Lithuania** | **1727.73(1379.01-2093.45)** | | **20323.89(16802.92-24467.58)** | **10.76(9.13-12.85)** | | **38.33(30.69-46.11)** | | **380.23(316.79-452.87)** | **8.23(7-9.48)** | |
| **Luxembourg** | **85.01(67.14-109.57)** | | **2621.11(2132.76-3210.05)** | **29.83(25.61-35.38)** | | **15.33(12.1-19.62)** | | **264.1(215.26-323.76)** | **10.31(8.37-12.29)** | |
| **Madagascar** | **76.33(61.56-94.29)** | | **187.74(149.18-234.57)** | **1.46(1.22-1.72)** | | **1.3(1.05-1.57)** | | **1.43(1.15-1.75)** | **0.33(-7.56-8.9)** | |
| **Malawi** | **35.32(26.4-45.3)** | | **94.51(70.85-122.71)** | **1.68(1.41-2.02)** | | **0.85(0.65-1.08)** | | **1.19(0.91-1.53)** | **1.18(-8.08-11.37)** | |
| **Malaysia** | **130.68(95.95-168.49)** | | **2872.11(2240.33-3645.64)** | **20.98(17.13-26.13)** | | **1.48(1.11-1.91)** | | **10.91(8.63-13.67)** | **7.12(0.97-13.65)** | |
| **Maldives** | **0.83(0.6-1.1)** | | **19.26(14.81-24.64)** | **22.21(17.89-27.66)** | | **0.9(0.68-1.16)** | | **6.77(5.23-8.68)** | **7.22(-0.62-15.67)** | |
| **Mali** | **41.28(31.98-53.42)** | | **115.23(89.04-146.46)** | **1.79(1.44-2.18)** | | **0.87(0.68-1.11)** | | **1.11(0.86-1.39)** | **0.85(-8.47-11.11)** | |
| **Malta** | **14.96(11.21-19.31)** | | **957.68(806.37-1156)** | **63.03(49.63-79.8)** | | **3.48(2.63-4.49)** | | **99.79(83.46-119.84)** | **12.27(8.21-16.49)** | |
| **Marshall Islands** | **0.41(0.32-0.52)** | | **2.57(1.99-3.24)** | **5.33(4.29-6.63)** | | **2.51(1.99-3.24)** | | **8.03(6.34-10.14)** | **4.09(-0.88-9.3)** | |
| **Mauritania** | **20.72(16.17-25.78)** | | **79.53(60.72-100.81)** | **2.84(2.3-3.5)** | | **1.86(1.45-2.31)** | | **3.33(2.55-4.2)** | **2.03(-4.09-8.54)** | |
| **Mauritius** | **12.46(9.46-16.17)** | | **252.47(194.87-325.82)** | **19.26(15.43-24.69)** | | **1.68(1.28-2.16)** | | **14.25(11.15-18.11)** | **7.65(1.88-13.76)** | |
| **Mexico** | **4472.64(3539.86-5478.12)** | | **50230.15(40976.03-60266.02)** | **10.23(9.18-11.46)** | | **9.96(7.87-12.2)** | | **41.39(33.68-49.4)** | **5.03(2.56-7.57)** | |
| **Micronesia (Federated States of)** | **2.16(1.66-2.78)** | | **14.35(11.06-18.31)** | **5.63(4.37-7.01)** | | **5.13(3.93-6.61)** | | **22.73(18.04-28.83)** | **5.27(1.85-8.8)** | |
| **Monaco** | **9.17(7.15-12.11)** | | **266.03(219.65-329.05)** | **28.02(22.81-34.99)** | | **12.96(10.11-17.05)** | | **291.41(242.55-359.55)** | **11.33(9.22-13.49)** | |
| **Mongolia** | **30.09(22.66-39.25)** | | **311.91(245.61-387.69)** | **9.37(7.57-12.04)** | | **2.73(2.08-3.54)** | | **11.74(9.38-14.38)** | **5.16(0.49-10.04)** | |
| **Montenegro** | **677.96(534.78-836.39)** | | **4187.44(3401.7-5087.17)** | **5.18(4.45-6.03)** | | **108.71(86.03-134.34)** | | **433.64(352.66-524.66)** | **4.89(4.13-5.65)** | |
| **Morocco** | **281.8(214.66-360.48)** | | **1124.52(868.78-1412.75)** | **2.99(2.53-3.55)** | | **1.83(1.39-2.32)** | | **3.24(2.5-4.04)** | **1.99(-4.19-8.57)** | |
| **Mozambique** | **46.59(35.62-60.15)** | | **147.97(115.79-187.11)** | **2.18(1.84-2.6)** | | **0.73(0.57-0.92)** | | **1.22(0.96-1.51)** | **1.8(-7.92-12.53)** | |
| **Myanmar** | **94.81(69.5-127.9)** | | **553.76(398.81-756.33)** | **4.84(3.92-5.98)** | | **0.4(0.3-0.54)** | | **1.2(0.87-1.6)** | **3.8(-8.2-17.38)** | |
| **Namibia** | **11.53(9.11-14.29)** | | **43.12(34.19-52.51)** | **2.74(2.36-3.22)** | | **1.56(1.25-1.92)** | | **2.9(2.34-3.54)** | **2.17(-4.47-9.26)** | |
| **Nauru** | **0.16(0.13-0.2)** | | **0.56(0.44-0.72)** | **2.56(2-3.23)** | | **5.56(4.36-7.24)** | | **17.2(13.58-21.33)** | **3.97(0.6-7.45)** | |
| **Nepal** | **97.9(74.03-127.43)** | | **497.06(381.93-625.63)** | **4.08(3.48-4.77)** | | **0.94(0.73-1.21)** | | **2.12(1.65-2.67)** | **2.85(-5.41-11.83)** | |
| **Netherlands** | **731.25(572.26-930.82)** | | **10494(8326.54-13411.48)** | **13.35(10.99-16.51)** | | **3.66(2.86-4.62)** | | **30.87(24.52-39.18)** | **7.63(3.68-11.73)** | |
| **New Zealand** | **3312.28(2685.2-4130.12)** | | **32327.95(27212.44-38142.77)** | **8.76(7.78-9.95)** | | **84.34(68.75-104.19)** | | **405.99(342.78-477.05)** | **5.57(4.72-6.43)** | |
| **Nicaragua** | **53.61(42.32-67.19)** | | **512.04(402.51-632.37)** | **8.55(7.32-10.21)** | | **3.12(2.46-3.89)** | | **10.36(8.19-12.7)** | **4.23(-0.23-8.88)** | |
| **Niger** | **30.38(23.25-39.44)** | | **96.44(72.74-125.09)** | **2.17(1.84-2.57)** | | **0.85(0.65-1.08)** | | **0.98(0.75-1.25)** | **0.51(-9.07-11.1)** | |
| **Nigeria** | **713.65(544.61-907.5)** | | **1762.22(1344.56-2233.3)** | **1.47(1.34-1.61)** | | **1.4(1.08-1.76)** | | **1.6(1.24-2.01)** | **0.46(-7.09-8.63)** | |
| **Niue** | **0.13(0.1-0.17)** | | **1.07(0.84-1.33)** | **7.11(5.57-8.92)** | | **5.82(4.59-7.26)** | | **49.02(38.72-60.88)** | **7.63(4.48-10.87)** | |
| **North Macedonia** | **875.55(693.02-1084.21)** | | **9897.65(8139.41-11956.66)** | **10.3(8.51-12.77)** | | **46.36(37.01-56.91)** | | **307.54(253.65-370.57)** | **6.74(5.61-7.89)** | |
| **Northern Mariana Islands** | **4.76(3.73-5.94)** | | **64.53(48.87-81.74)** | **12.56(10.01-15.77)** | | **34.32(27.14-43.3)** | | **128.64(102.16-159.79)** | **4.66(3.31-6.03)** | |
| **Norway** | **753.4(598.42-963.47)** | | **5517.47(4328.9-7065.83)** | **6.32(5.46-7.48)** | | **11.08(8.81-14.1)** | | **57.49(45.28-73.06)** | **5.84(3.52-8.22)** | |
| **Oman** | **32.19(24.97-40.84)** | | **320.17(237.3-413.5)** | **8.95(7.04-11.16)** | | **3.69(2.9-4.55)** | | **11.96(9.11-15.24)** | **4.14(0.03-8.42)** | |
| **Pakistan** | **1502.45(1180.71-1912.19)** | | **4105.9(3215.7-5109.79)** | **1.73(1.54-1.98)** | | **2.53(1.98-3.23)** | | **3.41(2.69-4.25)** | **1.03(-4.48-6.86)** | |
| **Palau** | **0.61(0.47-0.79)** | | **11.5(8.87-14.46)** | **17.72(13.53-22.82)** | | **6.56(4.98-8.42)** | | **56.57(44.48-71.27)** | **7.71(4.75-10.76)** | |
| **Palestine** | **30.46(22.48-39.73)** | | **96.39(70.33-127.67)** | **2.16(1.58-2.73)** | | **3.15(2.31-4.11)** | | **3.29(2.43-4.3)** | **0.14(-5.05-5.62)** | |
| **Panama** | **85.02(66.99-105.46)** | | **635(517.46-772.47)** | **6.47(5.35-7.83)** | | **5.44(4.29-6.82)** | | **15.29(12.49-18.55)** | **3.62(0.19-7.18)** | |
| **Papua New Guinea** | **35.03(27.52-44.69)** | | **241.03(186.43-300.26)** | **5.88(4.99-6.96)** | | **2.03(1.59-2.61)** | | **5.13(3.99-6.35)** | **3.25(-2.38-9.21)** | |
| **Paraguay** | **128.85(104.63-156.8)** | | **1447.74(1202.96-1725.02)** | **10.24(8.75-12.08)** | | **5.4(4.4-6.57)** | | **25.43(21.13-30.44)** | **5.49(2.17-8.92)** | |
| **Peru** | **377.49(296.52-477.57)** | | **11561.59(9339.29-14012.79)** | **29.63(24.05-36.17)** | | **2.91(2.3-3.64)** | | **35.9(29.07-43.6)** | **9.05(4.65-13.64)** | |
| **Philippines** | **186.74(134.55-255.68)** | | **1364.17(996.99-1820.8)** | **6.31(5.42-7.35)** | | **0.6(0.44-0.82)** | | **1.73(1.28-2.31)** | **3.7(-6.26-14.73)** | |
| **Poland** | **49435.81(39195.88-59538.54)** | | **258294.1(209260.22-305719.67)** | **4.22(3.85-4.68)** | | **113.21(89.83-135.96)** | | **382.71(310.52-454.22)** | **4.29(3.54-5.05)** | |
| **Portugal** | **303.36(227.84-401.74)** | | **11252.44(9002.53-14286.93)** | **36.09(28.36-45.72)** | | **2.15(1.63-2.86)** | | **46.47(37.66-57.77)** | **11.18(6.06-16.54)** | |
| **Puerto Rico** | **1943.66(1572.8-2413.51)** | | **28773.38(22813.31-35815.28)** | **13.8(11.14-16.93)** | | **53.47(43.26-66.24)** | | **457.61(362.96-575.53)** | **7.68(6.64-8.74)** | |
| **Qatar** | **282.77(213.91-362.25)** | | **2668.05(2054.81-3338.88)** | **8.44(7.17-9.83)** | | **108.48(83.11-138.8)** | | **137.22(105.59-171.6)** | **0.81(-0.06-1.69)** | |
| **Republic of Korea** | **28187.68(22924.39-34052.97)** | | **268834.29(226639.75-317267.62)** | **8.54(7.21-10.2)** | | **92.66(75.6-111.97)** | | **310.27(261.14-364.94)** | **4.26(3.42-5.09)** | |
| **Republic of Moldova** | **704.11(566.61-851.61)** | | **15713.66(12437.15-19127.86)** | **21.32(17.72-24.91)** | | **15.88(12.78-19.14)** | | **274.15(219.12-333.46)** | **10.32(8.41-12.26)** | |
| **Romania** | **32933.43(26571.79-40249.48)** | | **369312.63(300495.81-454223.07)** | **10.21(8.33-12.41)** | | **117.25(94.72-142.48)** | | **1044.49(855.59-1271.28)** | **7.83(7.13-8.55)** | |
| **Russian Federation** | **78538.22(62246.48-97561.91)** | | **1049883.7(839793.39-1270533.85)** | **12.37(11.29-13.57)** | | **42.95(34.28-53.09)** | | **456.35(365.58-553.15)** | **8.49(7.33-9.67)** | |
| **Rwanda** | **31.52(24.95-39.13)** | | **79.37(59.96-101.08)** | **1.52(1.22-1.85)** | | **1(0.8-1.24)** | | **1.22(0.94-1.53)** | **0.67(-8.1-10.29)** | |
| **Saint Kitts and Nevis** | **2.23(1.81-2.72)** | | **27.81(22.28-33.41)** | **11.47(9.5-14.02)** | | **6.13(5-7.47)** | | **39.52(32.26-47.2)** | **6.64(3.55-9.81)** | |
| **Saint Lucia** | **5.33(4.36-6.45)** | | **70.94(58.58-85.79)** | **12.3(10.22-15.03)** | | **5.89(4.84-7.13)** | | **32.61(26.88-39.31)** | **6.08(2.92-9.33)** | |
| **Saint Vincent and the Grenadines** | **3.63(2.91-4.5)** | | **41.59(34.22-50.19)** | **10.45(8.31-12.94)** | | **4.95(3.95-6.16)** | | **30.25(24.94-36.43)** | **6.44(3.01-9.98)** | |
| **Samoa** | **10.69(8.51-13.44)** | | **36.25(28.54-45.46)** | **2.39(1.95-2.96)** | | **13.31(10.55-16.77)** | | **26.44(21.08-33.1)** | **2.4(0.1-4.75)** | |
| **San Marino** | **2.17(1.65-2.82)** | | **109.42(85.33-131.74)** | **49.5(39.97-61.55)** | | **6.38(4.87-8.26)** | | **169.43(134.48-203.7)** | **11.97(8.96-15.07)** | |
| **Sao Tome and Principe** | **1.02(0.78-1.29)** | | **3.63(2.74-4.52)** | **2.57(2.1-3.18)** | | **1.48(1.13-1.87)** | | **2.76(2.11-3.4)** | **2.17(-4.63-9.46)** | |
| **Saudi Arabia** | **332.57(237.36-449.99)** | | **7647.25(5831.24-9605.12)** | **21.99(17.31-27.91)** | | **4.61(3.32-6.39)** | | **30.66(23.69-38.07)** | **6.75(3.21-10.41)** | |
| **Senegal** | **42.27(32.91-54.14)** | | **119.68(92.06-152.01)** | **1.83(1.52-2.15)** | | **1.12(0.88-1.42)** | | **1.34(1.05-1.7)** | **0.62(-7.71-9.7)** | |
| **Serbia** | **6605.75(5454.49-7854.37)** | | **78339.46(66344.62-92902.76)** | **10.86(9.09-13.47)** | | **57.34(48.14-67.6)** | | **492.89(417.41-579.51)** | **7.7(6.69-8.72)** | |
| **Seychelles** | **1.59(1.19-2.06)** | | **22.84(16.94-29.81)** | **13.38(10.36-17.44)** | | **2.79(2.1-3.6)** | | **20.89(15.47-27.26)** | **7.19(2.67-11.91)** | |
| **Sierra Leone** | **22.13(17.14-27.71)** | | **57.44(44.46-74.02)** | **1.6(1.3-1.93)** | | **1.04(0.81-1.31)** | | **1.27(1-1.6)** | **0.7(-7.9-10.11)** | |
| **Singapore** | **2258.8(1849.07-2745.74)** | | **34091.97(28074.26-40795.77)** | **14.09(12.64-15.72)** | | **98.11(80.67-120.32)** | | **433.97(358.54-518.38)** | **5.26(4.47-6.06)** | |
| **Slovakia** | **2433.63(1962.1-3038.17)** | | **36995.22(30865.34-43898.52)** | **14.2(11.79-17.1)** | | **40.65(32.66-50.46)** | | **410.75(342.55-482.3)** | **8.3(7.11-9.51)** | |
| **Slovenia** | **2708.27(2151.93-3349.95)** | | **44250.92(33263.15-56436.76)** | **15.34(11.98-20.24)** | | **110.19(87.57-136.18)** | | **1080.06(825.88-1367.13)** | **8.19(7.46-8.92)** | |
| **Solomon Islands** | **2.76(2.15-3.5)** | | **18.92(14.75-23.82)** | **5.85(4.9-6.97)** | | **2.37(1.84-3.1)** | | **6.73(5.28-8.49)** | **3.66(-1.5-9.09)** | |
| **Somalia** | **22.73(17.37-29.16)** | | **52.55(39.93-69.57)** | **1.31(1.01-1.62)** | | **0.78(0.62-0.98)** | | **0.66(0.52-0.85)** | **-0.56(-11.18-11.33)** | |
| **South Africa** | **2242.75(1746.86-2788.32)** | | **56993.16(43896.39-72883.15)** | **24.41(20.35-29.14)** | | **10.2(7.92-12.73)** | | **118.85(91.51-151.35)** | **8.84(6.46-11.26)** | |
| **South Sudan** | **29.96(22.84-38.46)** | | **53.28(39.7-70.07)** | **0.78(0.56-1.04)** | | **1.14(0.88-1.45)** | | **1.21(0.92-1.56)** | **0.21(-8.24-9.44)** | |
| **Spain** | **3318.68(2598.57-4181.55)** | | **82883.96(66763.67-102701.56)** | **23.98(19.56-29.01)** | | **6.14(4.83-7.7)** | | **85.32(69.27-105.43)** | **9.5(6.45-12.63)** | |
| **Sri Lanka** | **174.81(132.27-224)** | | **1070.27(818.46-1354.2)** | **5.12(4.31-6.18)** | | **1.61(1.22-2.06)** | | **4.07(3.16-5.14)** | **3.24(-3.05-9.94)** | |
| **Sudan** | **136.75(104.5-174.65)** | | **508.25(390.38-646.09)** | **2.72(2.31-3.2)** | | **1.31(1.01-1.68)** | | **2.33(1.82-2.97)** | **2.01(-5.24-9.82)** | |
| **Suriname** | **12.52(10.09-15.23)** | | **169.77(136.83-206.5)** | **12.56(10.74-14.76)** | | **4.6(3.7-5.57)** | | **27.52(22.3-33.2)** | **6.36(2.8-10.04)** | |
| **Sweden** | **5508.37(4430.19-6983.58)** | | **60974.82(49804.38-74278.39)** | **10.07(8.88-11.52)** | | **35.49(28.84-44.74)** | | **284.59(234.8-343.78)** | **7.44(6.16-8.74)** | |
| **Switzerland** | **1277.13(1002.43-1663.9)** | | **43793.56(36987.37-51576.63)** | **33.29(27.34-40.83)** | | **12.01(9.47-15.65)** | | **248.78(210.62-294.25)** | **11.02(8.82-13.26)** | |
| **Syrian Arab Republic** | **397.37(313.66-489.54)** | | **759.71(593.83-946.67)** | **0.91(0.64-1.22)** | | **6.71(5.29-8.26)** | | **5.57(4.4-6.86)** | **-0.64(-4.42-3.28)** | |
| **Taiwan (Province of China)** | **336.82(250.08-447.58)** | | **21911.17(18162.23-26764.15)** | **64.05(50.36-82.77)** | | **2.31(1.75-2.98)** | | **55.11(45.84-66.97)** | **11.56(6.61-16.74)** | |
| **Tajikistan** | **70.69(53.06-93.74)** | | **353.38(272.98-460.78)** | **4(3.09-5.11)** | | **2.4(1.79-3.16)** | | **5.72(4.47-7.19)** | **3.05(-2.17-8.55)** | |
| **Thailand** | **338.28(249.59-447.18)** | | **9793.35(7633.48-12302.78)** | **27.95(22.56-35.79)** | | **0.97(0.74-1.26)** | | **9.52(7.44-11.92)** | **8.19(0.67-16.26)** | |
| **Timor-Leste** | **1.21(0.86-1.66)** | | **9.77(7.19-13.11)** | **7.05(5.69-8.87)** | | **0.42(0.31-0.56)** | | **1.18(0.88-1.55)** | **3.59(-8.21-16.92)** | |
| **Togo** | **17.56(13.85-22.34)** | | **69.66(54.01-88.02)** | **2.97(2.59-3.43)** | | **1.13(0.89-1.4)** | | **1.48(1.17-1.82)** | **0.95(-7.23-9.86)** | |
| **Tokelau** | **0.04(0.03-0.05)** | | **0.24(0.19-0.31)** | **5.31(4.13-6.82)** | | **2.77(2.2-3.49)** | | **18.42(14.59-23.16)** | **6.75(2.2-11.5)** | |
| **Tonga** | **4.18(3.28-5.35)** | | **27.28(21.71-33.86)** | **5.53(4.58-6.66)** | | **8.28(6.5-10.65)** | | **35.43(28.24-44.19)** | **5.14(2.43-7.92)** | |
| **Trinidad and Tobago** | **60.43(47.21-75.27)** | | **825.74(656.35-1019.48)** | **12.67(10.39-15.64)** | | **6.83(5.36-8.54)** | | **43.89(35.16-53.79)** | **6.63(3.7-9.63)** | |
| **Tunisia** | **225.98(176.22-283.89)** | | **847.5(664.22-1070.55)** | **2.75(2.37-3.22)** | | **4.04(3.17-5.07)** | | **6.38(5-8.03)** | **1.59(-2.68-6.05)** | |
| **Turkey** | **1956.13(1540.41-2409.11)** | | **11166.87(8896.99-13574.35)** | **4.71(4.01-5.62)** | | **4.96(3.95-6.04)** | | **12.3(9.82-14.89)** | **3.18(-0.46-6.96)** | |
| **Turkmenistan** | **75.39(56.38-98.41)** | | **1858.73(1475.43-2276.77)** | **23.65(18.62-29.97)** | | **3.78(2.85-4.9)** | | **44.16(35.28-53.39)** | **8.85(4.97-12.86)** | |
| **Tuvalu** | **0.13(0.1-0.17)** | | **0.92(0.7-1.16)** | **6.04(5.01-7.35)** | | **2.06(1.61-2.6)** | | **9.09(7.06-11.4)** | **5.25(-0.1-10.88)** | |
| **Uganda** | **58.14(44.2-74.8)** | | **165.75(126.62-213)** | **1.85(1.56-2.2)** | | **0.83(0.64-1.05)** | | **1.06(0.82-1.33)** | **0.85(-8.67-11.37)** | |
| **Ukraine** | **34724.81(27665.19-42098.95)** | | **168865.68(133883.47-206428.54)** | **3.86(3.45-4.34)** | | **49.15(39.26-59.28)** | | **232.44(185.06-282.36)** | **5.5(4.39-6.63)** | |
| **United Arab Emirates** | **169.69(124.75-229.73)** | | **5738.16(4365.13-7301.28)** | **32.82(27.67-39.79)** | | **18.86(14.42-24.46)** | | **69.23(53.5-88.41)** | **4.59(2.77-6.44)** | |
| **United Kingdom** | **108478.53(86136.4-137223.32)** | | **431375.62(360324.87-516764.55)** | **2.98(2.49-3.57)** | | **118.65(94.51-149.28)** | | **341.24(287.07-405.19)** | **3.71(2.97-4.46)** | |
| **United Republic of Tanzania** | **122.41(92.4-155.5)** | | **409.41(310.76-524.15)** | **2.34(1.98-2.8)** | | **1.06(0.81-1.33)** | | **1.54(1.18-1.95)** | **1.31(-6.98-10.34)** | |
| **United States of America** | **685737.93(556401.45-838472.59)** | | **1425072.93(1243341.34-1649908.1)** | **1.08(0.86-1.36)** | | **210.23(171.24-256.04)** | | **261.92(229.32-299.88)** | **0.76(0.13-1.39)** | |
| **United States Virgin Islands** | **29.67(24.07-36.67)** | | **484.32(387.75-594.92)** | **15.32(12.91-18.13)** | | **32.95(27-40.62)** | | **263.88(211.63-324)** | **7.44(6.1-8.79)** | |
| **Uruguay** | **494.36(380.92-644.57)** | | **7000.64(5879.22-8385.87)** | **13.16(10.11-16.73)** | | **12.71(9.88-16.44)** | | **129.03(108.73-153.35)** | **8.32(6.19-10.49)** | |
| **Uzbekistan** | **427.11(319.03-556.52)** | | **7733.27(6101.91-9524.8)** | **17.11(13.42-21.72)** | | **3.65(2.74-4.73)** | | **33.6(27.16-40.4)** | **7.95(4.01-12.04)** | |
| **Vanuatu** | **1.82(1.43-2.32)** | | **14.06(10.98-17.79)** | **6.73(5.71-8.07)** | | **3.01(2.35-3.9)** | | **8.39(6.57-10.66)** | **3.6(-1-8.41)** | |
| **Venezuela (Bolivarian Republic of)** | **654.8(534.12-788.01)** | | **6324.83(5184.92-7603.21)** | **8.66(7.43-9.99)** | | **6.51(5.29-7.84)** | | **21.17(17.36-25.42)** | **4.15(1.04-7.35)** | |
| **Viet Nam** | **257.45(192.86-341.62)** | | **1795.72(1332.69-2367.95)** | **5.97(4.86-7.62)** | | **0.63(0.48-0.83)** | | **1.93(1.44-2.52)** | **3.91(-5.77-14.59)** | |
| **Yemen** | **74.94(58.77-95.99)** | | **268.12(207.88-346.59)** | **2.58(2.21-2.99)** | | **1.27(1-1.61)** | | **1.67(1.31-2.11)** | **0.93(-6.79-9.29)** | |
| **Zambia** | **27.85(20.75-35.89)** | | **102.89(77.94-133.08)** | **2.69(2.24-3.27)** | | **0.91(0.7-1.17)** | | **1.38(1.06-1.76)** | **1.45(-7.4-11.14)** | |
| **Zimbabwe** | **76.67(59.65-96.05)** | | **172.4(137.63-210)** | **1.25(1.03-1.5)** | | **1.77(1.39-2.21)** | | **2.27(1.83-2.77)** | **0.86(-5.75-7.93)** | |
| **Abbreviations: CAVD, calcific aortic valve disease; EAPC, estimated annual percentage change; ASPR, age standardized prevalence rate; UI, uncertainty interval; CI, confidence interval.** | | | | | | | | | | |
