# Supplementary material for: Changing epidemiology of calcific aortic valve disease: 30-year trends of incidence, prevalence, and deaths across 204 countries and territories
Source: Aging (Albany NY). 2021 May 11;13(9):12710–32. doi: 10.18632/aging.202942 (PMC8148466; doi:10.18632/aging.202942)
Supplement: Supplementary Table 4 [file aging-13-202942-s005.docx]

**Supplementary Table 4. The change of CAVD deaths between 1990 and 2019 and EAPC at 204 countries and territories.**

|  |  | **All-ages Deaths** | | |  |  | **ASDR** | | |  |
| --- | --- | --- | --- | --- | --- | --- | --- | --- | --- | --- |
| **Location** | **1990 No. (95% UI)** | | **2019 No. (95% UI)** | **Change in Absolute Number (95% UI)** | | **1990 per 100,000 No. (95% UI)** | | **2019 per 100,000 No. (95% UI)** | **EAPC No. (95% CI)** | |
| **Afghanistan** | **57.76(23.78-96.85)** | | **112.26(57.48-179.7)** | **0.94(0.39-1.93)** | | **0.89(0.37-1.42)** | | **0.87(0.44-1.31)** | **-0.07(-9.76-10.66)** | |
| **Albania** | **7.75(5.77-11.66)** | | **23.25(16.18-31.33)** | **2(0.79-4.02)** | | **0.45(0.32-0.7)** | | **0.56(0.39-0.75)** | **0.76(-12-15.38)** | |
| **Algeria** | **120.43(89.79-158.97)** | | **294.01(191.27-400.16)** | **1.44(0.65-2.63)** | | **1.18(0.89-1.56)** | | **0.98(0.65-1.3)** | **-0.63(-9.4-8.99)** | |
| **American Samoa** | **0.19(0.15-0.24)** | | **0.42(0.34-0.52)** | **1.22(0.67-2.02)** | | **1.1(0.84-1.41)** | | **1.05(0.83-1.32)** | **-0.18(-8.97-9.46)** | |
| **Andorra** | **0.72(0.49-1.03)** | | **3.13(2.27-4.11)** | **3.32(1.73-5.77)** | | **2.02(1.4-2.8)** | | **1.92(1.39-2.53)** | **-0.18(-6.76-6.86)** | |
| **Angola** | **27.85(15.99-44.58)** | | **86.54(65.52-114.66)** | **2.11(1.02-4.16)** | | **0.9(0.54-1.39)** | | **1.06(0.8-1.38)** | **0.55(-8.74-10.78)** | |
| **Antigua and Barbuda** | **0.48(0.4-0.56)** | | **0.85(0.7-1.08)** | **0.77(0.4-1.23)** | | **0.85(0.71-1)** | | **0.94(0.77-1.19)** | **0.35(-9.29-11.01)** | |
| **Argentina** | **1106.44(961.1-1257.2)** | | **2165.88(1869.33-2414.45)** | **0.96(0.69-1.27)** | | **3.92(3.39-4.44)** | | **3.87(3.34-4.3)** | **-0.04(-4.77-4.92)** | |
| **Armenia** | **4.8(4.08-5.5)** | | **14.93(12.15-17.93)** | **2.11(1.34-2.99)** | | **0.19(0.16-0.22)** | | **0.38(0.32-0.46)** | **2.4(-15.18-23.63)** | |
| **Australia** | **605.93(538.56-674.41)** | | **1434.82(1166.87-1648.12)** | **1.37(1.08-1.63)** | | **3.29(2.89-3.66)** | | **2.89(2.36-3.31)** | **-0.45(-5.73-5.13)** | |
| **Austria** | **344.46(282.35-386.18)** | | **979.92(787.42-1133)** | **1.84(1.36-2.5)** | | **2.84(2.34-3.22)** | | **4.31(3.52-4.96)** | **1.44(-3.66-6.82)** | |
| **Azerbaijan** | **6.9(5.68-8.23)** | | **15.8(12.44-20.21)** | **1.29(0.73-2.07)** | | **0.14(0.11-0.17)** | | **0.19(0.15-0.24)** | **1.13(-20.45-28.55)** | |
| **Bahamas** | **1.23(1.02-1.41)** | | **3.32(2.7-4.15)** | **1.71(1.12-2.48)** | | **0.82(0.68-0.94)** | | **0.92(0.75-1.14)** | **0.37(-9.41-11.22)** | |
| **Bahrain** | **1.59(1.3-1.96)** | | **6.86(5.23-8.89)** | **3.33(2.16-5.15)** | | **1.47(1.16-1.86)** | | **1.42(1.12-1.79)** | **-0.12(-7.76-8.15)** | |
| **Bangladesh** | **280.43(157.32-390.28)** | | **932.92(615.85-1326.07)** | **2.33(0.96-4.87)** | | **0.73(0.43-0.99)** | | **0.85(0.56-1.22)** | **0.55(-9.73-12.01)** | |
| **Barbados** | **2.98(2.57-3.42)** | | **5.37(4.44-6.42)** | **0.8(0.43-1.21)** | | **1(0.86-1.14)** | | **1.11(0.92-1.33)** | **0.39(-8.54-10.19)** | |
| **Belarus** | **17.44(11.93-22.59)** | | **29.07(22.3-37.94)** | **0.67(0.22-1.65)** | | **0.14(0.1-0.18)** | | **0.18(0.14-0.24)** | **0.95(-20.6-28.35)** | |
| **Belgium** | **662.24(562.62-813.13)** | | **1703.05(1323.47-1960.29)** | **1.57(1.03-2.04)** | | **4.24(3.59-5.22)** | | **5.44(4.33-6.21)** | **0.86(-3.46-5.38)** | |
| **Belize** | **0.39(0.24-0.55)** | | **1.32(1.09-1.69)** | **2.4(1.15-4.42)** | | **0.42(0.26-0.59)** | | **0.51(0.41-0.64)** | **0.69(-12.58-15.97)** | |
| **Benin** | **11.67(6.33-17.91)** | | **29.61(17.19-46.7)** | **1.54(0.83-2.82)** | | **0.58(0.32-0.88)** | | **0.6(0.36-0.92)** | **0.11(-11.61-13.39)** | |
| **Bermuda** | **3.1(2.37-3.61)** | | **4.74(3.86-5.78)** | **0.53(0.19-1.1)** | | **5.29(4.07-6.12)** | | **3.51(2.9-4.29)** | **-1.4(-5.88-3.3)** | |
| **Bhutan** | **1.16(0.5-1.97)** | | **4.32(2.91-5.94)** | **2.72(1.26-6.37)** | | **0.65(0.3-1.07)** | | **0.91(0.61-1.25)** | **1.19(-9.33-12.92)** | |
| **Bolivia (Plurinational State of)** | **24.79(14.39-37.8)** | | **71.36(50.41-100.18)** | **1.88(0.96-3.7)** | | **0.82(0.48-1.22)** | | **0.88(0.62-1.22)** | **0.22(-9.65-11.16)** | |
| **Bosnia and Herzegovina** | **31.79(21.56-41.06)** | | **80.81(61.75-106.79)** | **1.54(0.8-2.88)** | | **1.07(0.74-1.39)** | | **1.45(1.11-1.89)** | **1.08(-7.27-10.17)** | |
| **Botswana** | **3.81(2.67-5.52)** | | **9.24(6.24-13.95)** | **1.42(0.71-2.54)** | | **0.86(0.61-1.22)** | | **0.89(0.62-1.32)** | **0.13(-9.58-10.89)** | |
| **Brazil** | **1507.45(1326.04-1636.67)** | | **3467.29(3000.8-4002.58)** | **1.3(1.07-1.6)** | | **1.83(1.6-1.97)** | | **1.54(1.33-1.78)** | **-0.58(-7.66-7.04)** | |
| **Brunei Darussalam** | **1.22(0.96-1.64)** | | **4.99(4.08-5.84)** | **3.09(1.94-4.53)** | | **2.02(1.56-2.62)** | | **3.29(2.59-3.99)** | **1.7(-4.26-8.04)** | |
| **Bulgaria** | **24(17.81-36.89)** | | **69.08(52.01-88.83)** | **1.88(0.66-3.38)** | | **0.21(0.16-0.33)** | | **0.48(0.36-0.62)** | **2.88(-13.7-22.63)** | |
| **Burkina Faso** | **24.43(12.17-37.94)** | | **65.1(37.37-101.62)** | **1.67(0.98-2.72)** | | **0.58(0.29-0.91)** | | **0.73(0.44-1.14)** | **0.83(-10.48-13.55)** | |
| **Burundi** | **22.07(12.05-37.8)** | | **28.53(19.13-40.71)** | **0.29(-0.16-1.14)** | | **1.06(0.62-1.74)** | | **0.81(0.57-1.17)** | **-0.93(-10.32-9.45)** | |
| **Cabo Verde** | **0.98(0.76-1.21)** | | **3.12(2.42-4)** | **2.18(1.3-3.49)** | | **0.43(0.33-0.53)** | | **0.7(0.55-0.9)** | **1.73(-10.78-15.99)** | |
| **Cambodia** | **8.65(5.3-14.35)** | | **22.67(16.52-34.83)** | **1.62(0.74-3.03)** | | **0.22(0.14-0.36)** | | **0.22(0.16-0.34)** | **-0.06(-18.43-22.45)** | |
| **Cameroon** | **25.89(14.76-39.54)** | | **82.27(49.37-131.72)** | **2.18(1.18-3.91)** | | **0.59(0.33-0.91)** | | **0.68(0.43-1.04)** | **0.47(-10.92-13.32)** | |
| **Canada** | **772.71(673.36-890.38)** | | **2358.59(1900.88-2649.44)** | **2.05(1.61-2.45)** | | **2.47(2.14-2.85)** | | **2.93(2.4-3.28)** | **0.59(-5.11-6.64)** | |
| **Central African Republic** | **10.1(5.21-16.82)** | | **16.8(9-26.99)** | **0.66(0.22-1.3)** | | **1.1(0.62-1.78)** | | **1.04(0.61-1.53)** | **-0.18(-8.99-9.47)** | |
| **Chad** | **13.71(7.47-21.33)** | | **30.73(17.01-49.8)** | **1.24(0.62-2.14)** | | **0.5(0.27-0.76)** | | **0.54(0.3-0.86)** | **0.28(-12.22-14.56)** | |
| **Chile** | **142.78(126.4-156.91)** | | **334.95(293.92-384.51)** | **1.35(1.02-1.73)** | | **1.57(1.38-1.73)** | | **1.41(1.24-1.62)** | **-0.36(-7.88-7.76)** | |
| **China** | **1461.78(926.43-2080.7)** | | **2798.63(2224.69-3395.63)** | **0.91(0.24-2.35)** | | **0.18(0.11-0.26)** | | **0.15(0.12-0.18)** | **-0.65(-21.71-26.09)** | |
| **Colombia** | **255.61(228.49-283.54)** | | **663.55(478.02-933.29)** | **1.6(0.89-2.59)** | | **1.48(1.32-1.66)** | | **1.25(0.9-1.76)** | **-0.58(-8.42-7.93)** | |
| **Comoros** | **1.81(1.04-2.79)** | | **3.6(2.65-4.88)** | **0.99(0.31-2.49)** | | **0.94(0.58-1.37)** | | **0.85(0.62-1.16)** | **-0.33(-9.9-10.25)** | |
| **Congo** | **10.31(6.21-15.87)** | | **22.35(15.94-31.39)** | **1.17(0.41-2.4)** | | **1.23(0.78-1.74)** | | **1.15(0.87-1.57)** | **-0.21(-8.59-8.93)** | |
| **Cook Islands** | **0.06(0.04-0.07)** | | **0.07(0.06-0.09)** | **0.3(-0.08-0.86)** | | **0.49(0.37-0.64)** | | **0.32(0.26-0.41)** | **-1.4(-15.41-14.93)** | |
| **Costa Rica** | **17.52(14.84-19.86)** | | **67.39(51.3-86.75)** | **2.85(1.88-4.08)** | | **1.03(0.87-1.17)** | | **1.31(1-1.69)** | **0.85(-7.75-10.25)** | |
| **Croatia** | **33.33(20.51-69.8)** | | **297.74(207.67-381.75)** | **7.93(2.15-14.79)** | | **0.57(0.36-1.18)** | | **3.04(2.13-3.89)** | **5.95(-3.9-16.82)** | |
| **Cuba** | **94.48(81.87-105.57)** | | **195.39(152.26-258.03)** | **1.07(0.59-1.72)** | | **0.92(0.8-1.03)** | | **1.03(0.8-1.37)** | **0.39(-8.89-10.61)** | |
| **Cyprus** | **59.12(45.64-75.44)** | | **134.17(112.16-158.02)** | **1.27(0.75-2.01)** | | **10.21(7.64-13.17)** | | **8.2(6.86-9.76)** | **-0.75(-3.85-2.44)** | |
| **Czechia** | **37.81(24.25-66)** | | **597.9(436.91-776.84)** | **14.81(6.27-26.81)** | | **0.28(0.18-0.5)** | | **2.69(1.97-3.51)** | **8.09(-5.44-23.55)** | |
| **Côte d'Ivoire** | **23.7(13.14-36.35)** | | **60.31(35.96-93.73)** | **1.54(0.76-2.76)** | | **0.56(0.32-0.85)** | | **0.56(0.34-0.84)** | **-0.05(-12.03-13.56)** | |
| **Democratic People's Republic of Korea** | **37.07(26.43-53.7)** | | **62.24(46.01-85.92)** | **0.68(0.19-1.35)** | | **0.24(0.17-0.34)** | | **0.2(0.15-0.28)** | **-0.62(-19.01-21.94)** | |
| **Democratic Republic of the Congo** | **113.75(68.24-187.97)** | | **269.52(173.94-399.89)** | **1.37(0.66-2.5)** | | **0.95(0.63-1.44)** | | **0.97(0.66-1.38)** | **0.06(-9.24-10.31)** | |
| **Denmark** | **230.63(199.94-261.41)** | | **603.66(485.47-686.82)** | **1.62(1.24-2.04)** | | **2.61(2.26-2.95)** | | **4.53(3.69-5.15)** | **1.92(-3.3-7.41)** | |
| **Djibouti** | **1.05(0.74-1.52)** | | **4.21(2.84-6.16)** | **2.99(1.51-5.35)** | | **0.96(0.71-1.37)** | | **0.99(0.66-1.43)** | **0.08(-9.15-10.26)** | |
| **Dominica** | **0.67(0.53-0.82)** | | **0.93(0.72-1.19)** | **0.38(0.04-0.85)** | | **0.92(0.73-1.1)** | | **1.03(0.79-1.32)** | **0.41(-8.88-10.65)** | |
| **Dominican Republic** | **26.55(21.27-31.65)** | | **75.12(57.57-97.24)** | **1.83(1.11-2.8)** | | **0.76(0.61-0.9)** | | **0.85(0.66-1.09)** | **0.41(-9.77-11.73)** | |
| **Ecuador** | **30.96(25.02-38.71)** | | **122.5(93.38-159.79)** | **2.96(1.84-4.56)** | | **0.59(0.47-0.73)** | | **0.86(0.66-1.11)** | **1.3(-9.65-13.57)** | |
| **Egypt** | **287.54(198.31-405.03)** | | **657.7(471.27-928.72)** | **1.29(0.62-2.46)** | | **1.04(0.7-1.48)** | | **1.11(0.79-1.54)** | **0.21(-8.63-9.91)** | |
| **El Salvador** | **8.31(6.79-9.94)** | | **16.14(12.13-20.94)** | **0.94(0.42-1.72)** | | **0.27(0.22-0.33)** | | **0.27(0.2-0.35)** | **-0.04(-16.85-20.16)** | |
| **Equatorial Guinea** | **1.54(0.81-2.63)** | | **4.27(2.76-6.16)** | **1.76(0.3-5.09)** | | **0.95(0.52-1.58)** | | **1.15(0.74-1.64)** | **0.66(-8.33-10.52)** | |
| **Eritrea** | **7.1(3.12-12.41)** | | **18.5(11.91-28.07)** | **1.6(0.73-3.56)** | | **0.9(0.41-1.49)** | | **0.95(0.66-1.32)** | **0.19(-9.29-10.67)** | |
| **Estonia** | **8.45(6.26-9.88)** | | **80.46(59.07-108.48)** | **8.52(5.58-13.96)** | | **0.43(0.32-0.5)** | | **2.57(1.89-3.45)** | **6.35(-4.85-18.88)** | |
| **Eswatini** | **1.8(1.18-2.72)** | | **3.67(2.38-5.63)** | **1.04(0.45-1.92)** | | **0.77(0.51-1.16)** | | **0.86(0.57-1.25)** | **0.39(-9.7-11.61)** | |
| **Ethiopia** | **135.44(56.82-227.48)** | | **241.07(177.1-333.91)** | **0.78(0.03-2.81)** | | **0.85(0.41-1.34)** | | **0.71(0.53-1.01)** | **-0.59(-10.81-10.81)** | |
| **Fiji** | **1.5(1.22-1.87)** | | **2.42(1.89-3.04)** | **0.62(0.21-1.2)** | | **0.5(0.4-0.62)** | | **0.43(0.33-0.54)** | **-0.56(-13.6-14.44)** | |
| **Finland** | **293.5(239.52-337.39)** | | **744.08(594.53-863.21)** | **1.54(1.07-2.07)** | | **4.08(3.35-4.69)** | | **4.9(3.95-5.67)** | **0.63(-3.83-5.29)** | |
| **France** | **4427.85(3917.62-4954.51)** | | **8028.8(6387-9224.84)** | **0.81(0.54-1.07)** | | **4.97(4.4-5.58)** | | **4.04(3.28-4.61)** | **-0.71(-5.11-3.89)** | |
| **Gabon** | **5.48(3.61-8.19)** | | **10.41(7.95-13.41)** | **0.9(0.29-1.85)** | | **1.16(0.81-1.67)** | | **1.23(0.94-1.6)** | **0.2(-8.18-9.34)** | |
| **Gambia** | **1.87(1.06-2.82)** | | **6.03(3.84-9.1)** | **2.22(1.19-3.93)** | | **0.53(0.31-0.8)** | | **0.62(0.4-0.93)** | **0.55(-11.4-14.13)** | |
| **Georgia** | **9.41(7.45-11.55)** | | **44.11(30.79-67)** | **3.69(2.14-6.86)** | | **0.16(0.13-0.2)** | | **0.69(0.49-1.05)** | **5.15(-12.74-26.72)** | |
| **Germany** | **6092.26(5147.97-6933.23)** | | **13154.48(11098.62-15131.62)** | **1.16(0.81-1.56)** | | **4.63(3.93-5.24)** | | **5.47(4.61-6.26)** | **0.58(-3.63-4.96)** | |
| **Ghana** | **29.2(16.21-48.69)** | | **88.72(55.29-139.03)** | **2.04(1.26-3.42)** | | **0.49(0.28-0.78)** | | **0.59(0.38-0.88)** | **0.65(-11.69-14.73)** | |
| **Greece** | **346.66(290.74-382.54)** | | **1077.25(906.59-1241.47)** | **2.11(1.62-2.83)** | | **2.44(2.04-2.71)** | | **3.33(2.83-3.83)** | **1.07(-4.52-6.99)** | |
| **Greenland** | **1.03(0.53-1.71)** | | **2.1(1.27-2.95)** | **1.03(0.43-2.03)** | | **4.63(2.31-7.56)** | | **4.37(2.66-6.15)** | **-0.2(-4.59-4.41)** | |
| **Grenada** | **0.81(0.54-1.04)** | | **1.16(0.96-1.5)** | **0.43(0.02-1.21)** | | **1.05(0.7-1.35)** | | **1.12(0.93-1.44)** | **0.22(-8.57-9.86)** | |
| **Guam** | **0.93(0.76-1.13)** | | **2.76(2.21-3.33)** | **1.97(1.11-2.95)** | | **1.7(1.35-2.13)** | | **1.57(1.26-1.9)** | **-0.27(-7.46-7.47)** | |
| **Guatemala** | **12.34(8.48-16.3)** | | **34.89(26.94-44.32)** | **1.83(0.94-3.43)** | | **0.41(0.3-0.53)** | | **0.35(0.28-0.44)** | **-0.5(-14.86-16.28)** | |
| **Guinea** | **17.14(9.56-26.85)** | | **33.82(19.63-53.25)** | **0.97(0.41-1.74)** | | **0.54(0.3-0.84)** | | **0.61(0.36-0.96)** | **0.44(-11.51-14)** | |
| **Guinea-Bissau** | **2.84(1-5.29)** | | **5.33(2.66-9.19)** | **0.87(0.25-2.38)** | | **0.69(0.26-1.25)** | | **0.71(0.37-1.19)** | **0.12(-10.68-12.22)** | |
| **Guyana** | **10.86(8.85-12.91)** | | **11.15(8.49-14.57)** | **0.03(-0.25-0.4)** | | **2.88(2.37-3.42)** | | **1.9(1.49-2.44)** | **-1.42(-7.45-5)** | |
| **Haiti** | **37.47(20.5-54.45)** | | **73.19(41.74-113.98)** | **0.95(0.41-1.79)** | | **1.36(0.77-1.9)** | | **1.22(0.73-1.82)** | **-0.38(-8.43-8.37)** | |
| **Honduras** | **10.87(8.28-14.54)** | | **43.66(34.56-54.65)** | **3.02(2.07-4.3)** | | **0.56(0.41-0.77)** | | **0.81(0.64-1.02)** | **1.24(-9.97-13.84)** | |
| **Hungary** | **315.54(252.63-348.62)** | | **819.55(640.56-996.56)** | **1.6(1.02-2.32)** | | **2.23(1.79-2.47)** | | **3.95(3.11-4.8)** | **1.99(-3.62-7.93)** | |
| **Iceland** | **10.99(9.62-12.42)** | | **25.31(19.89-29.12)** | **1.3(0.91-1.68)** | | **3.59(3.15-4.05)** | | **3.7(2.96-4.24)** | **0.11(-4.78-5.25)** | |
| **India** | **2356.21(1416.24-3404.08)** | | **6861.86(5180.92-8944.67)** | **1.91(1.21-3.12)** | | **0.75(0.47-1.05)** | | **0.73(0.56-0.94)** | **-0.1(-10.59-11.62)** | |
| **Indonesia** | **215.96(169.41-299.91)** | | **539.98(361.66-748.11)** | **1.5(0.71-2.48)** | | **0.26(0.2-0.35)** | | **0.3(0.21-0.42)** | **0.57(-16.07-20.51)** | |
| **Iran (Islamic Republic of)** | **240.97(189.91-296.94)** | | **629.01(563.85-761.1)** | **1.61(1.15-2.43)** | | **1.05(0.83-1.32)** | | **0.9(0.8-1.11)** | **-0.51(-9.72-9.64)** | |
| **Iraq** | **23.08(16.75-32.95)** | | **78.96(54.08-104.37)** | **2.42(1.22-4.66)** | | **0.3(0.21-0.43)** | | **0.35(0.25-0.46)** | **0.59(-14.97-19.01)** | |
| **Ireland** | **118.4(107.52-133.7)** | | **238.6(199.8-272.21)** | **1.02(0.73-1.29)** | | **3.03(2.74-3.42)** | | **3.04(2.55-3.47)** | **0.01(-5.33-5.65)** | |
| **Israel** | **162.08(140.36-183.56)** | | **488.43(395.87-555.36)** | **2.01(1.51-2.49)** | | **3.83(3.28-4.35)** | | **3.68(3.02-4.18)** | **-0.13(-4.94-4.91)** | |
| **Italy** | **1147.49(937.05-1448.98)** | | **4375.67(3436.01-4957.02)** | **2.81(1.74-3.31)** | | **1.29(1.05-1.65)** | | **2.3(1.83-2.59)** | **2.01(-5.29-9.88)** | |
| **Jamaica** | **4.63(3.82-5.49)** | | **9.84(7.54-13.05)** | **1.12(0.6-1.85)** | | **0.25(0.21-0.3)** | | **0.31(0.24-0.41)** | **0.76(-15.99-20.83)** | |
| **Japan** | **4152.65(3526.06-4922.57)** | | **12868.42(8511.89-15732.07)** | **2.1(1.37-2.53)** | | **2.81(2.35-3.35)** | | **2.14(1.49-2.55)** | **-0.94(-6.83-5.33)** | |
| **Jordan** | **9.95(7.93-12.28)** | | **27.28(22.15-33.56)** | **1.74(1.06-2.77)** | | **0.85(0.68-1.04)** | | **0.49(0.4-0.6)** | **-1.88(-13.08-10.76)** | |
| **Kazakhstan** | **15.23(11.37-18.56)** | | **33.32(26.66-43.21)** | **1.19(0.68-2.17)** | | **0.13(0.09-0.16)** | | **0.21(0.17-0.26)** | **1.75(-20-29.41)** | |
| **Kenya** | **50.4(39.75-69.5)** | | **151(116.23-195.97)** | **2(1.3-2.9)** | | **0.73(0.58-0.97)** | | **0.92(0.67-1.25)** | **0.78(-9.35-12.05)** | |
| **Kiribati** | **0.24(0.17-0.42)** | | **0.4(0.28-0.62)** | **0.68(0.19-1.48)** | | **0.74(0.54-1.4)** | | **0.7(0.53-1.2)** | **-0.19(-10.79-11.68)** | |
| **Kuwait** | **3.71(3.11-4.25)** | | **20.71(16.78-24.89)** | **4.59(3.3-5.96)** | | **0.69(0.55-0.81)** | | **1.06(0.84-1.26)** | **1.46(-8.61-12.63)** | |
| **Kyrgyzstan** | **5.72(4.94-6.99)** | | **7.82(6.27-9.54)** | **0.37(0.07-0.72)** | | **0.18(0.16-0.22)** | | **0.18(0.14-0.21)** | **-0.14(-20.28-25.1)** | |
| **Lao People's Democratic Republic** | **4.31(2.48-7.72)** | | **9.83(7.1-14.4)** | **1.28(0.41-2.8)** | | **0.25(0.15-0.42)** | | **0.26(0.19-0.37)** | **0.17(-17.21-21.19)** | |
| **Latvia** | **15.51(13.32-17.46)** | | **42.33(33.94-51.33)** | **1.73(1.08-2.51)** | | **0.44(0.38-0.49)** | | **0.97(0.79-1.18)** | **2.77(-9.11-16.21)** | |
| **Lebanon** | **26.45(18.48-34.18)** | | **57.68(38.63-84.35)** | **1.18(0.57-2.26)** | | **1.29(0.9-1.67)** | | **1.11(0.74-1.61)** | **-0.52(-8.86-8.58)** | |
| **Lesotho** | **5.59(3.66-8.34)** | | **9.01(5.73-14.38)** | **0.61(0.17-1.21)** | | **0.7(0.47-1.04)** | | **0.97(0.63-1.48)** | **1.14(-9.02-12.43)** | |
| **Liberia** | **5.57(3.02-8.55)** | | **11.42(6.72-17.85)** | **1.05(0.43-2.15)** | | **0.53(0.29-0.82)** | | **0.53(0.32-0.82)** | **0.02(-12.27-14.02)** | |
| **Libya** | **14.17(8.61-19.49)** | | **38.13(22.89-53.94)** | **1.69(0.99-2.84)** | | **0.74(0.44-1.06)** | | **0.75(0.43-1.07)** | **0.03(-10.44-11.73)** | |
| **Lithuania** | **11.58(9.2-13.38)** | | **45.2(35.41-55.36)** | **2.9(1.93-4.36)** | | **0.26(0.21-0.3)** | | **0.75(0.58-0.93)** | **3.73(-11.08-21)** | |
| **Luxembourg** | **23.4(20.48-26.84)** | | **55.99(44.51-66.65)** | **1.39(0.95-1.86)** | | **4.49(3.92-5.16)** | | **4.71(3.78-5.57)** | **0.16(-4.21-4.73)** | |
| **Madagascar** | **58.69(38.97-87.37)** | | **104.8(69.41-147.76)** | **0.79(0.29-1.54)** | | **1.23(0.86-1.7)** | | **1.17(0.81-1.58)** | **-0.17(-8.52-8.94)** | |
| **Malawi** | **25.53(17.57-36.86)** | | **54(39.47-73.15)** | **1.11(0.41-2.17)** | | **0.85(0.62-1.21)** | | **0.91(0.65-1.27)** | **0.24(-9.49-11.02)** | |
| **Malaysia** | **32.59(27.3-38.61)** | | **119.72(92.97-154.59)** | **2.67(1.74-3.97)** | | **0.39(0.32-0.46)** | | **0.52(0.4-0.66)** | **1.02(-12.51-16.64)** | |
| **Maldives** | **0.39(0.22-0.62)** | | **1.71(1.36-2.16)** | **3.44(1.57-7.53)** | | **0.51(0.29-0.79)** | | **0.65(0.51-0.83)** | **0.87(-11.13-14.48)** | |
| **Mali** | **20.18(8.95-35.53)** | | **45.01(25.86-76.46)** | **1.23(0.59-2.58)** | | **0.52(0.24-0.91)** | | **0.54(0.32-0.87)** | **0.11(-12.19-14.13)** | |
| **Malta** | **7.1(6.25-8.16)** | | **19.16(15.58-22.29)** | **1.7(1.24-2.2)** | | **1.86(1.63-2.14)** | | **1.85(1.53-2.15)** | **-0.01(-6.78-7.26)** | |
| **Marshall Islands** | **0.17(0.1-0.26)** | | **0.27(0.17-0.42)** | **0.58(0.17-1.13)** | | **1.23(0.76-1.77)** | | **1.1(0.72-1.61)** | **-0.38(-8.83-8.85)** | |
| **Mauritania** | **6.19(3.61-9.43)** | | **10(7.05-14.41)** | **0.62(0.06-1.73)** | | **0.63(0.37-0.95)** | | **0.49(0.35-0.69)** | **-0.83(-12.79-12.78)** | |
| **Mauritius** | **4.58(3.69-5.81)** | | **8.91(7.21-11.2)** | **0.94(0.45-1.65)** | | **0.61(0.5-0.78)** | | **0.57(0.46-0.72)** | **-0.25(-11.89-12.94)** | |
| **Mexico** | **244.37(226.11-280.48)** | | **823.46(686.02-980.26)** | **2.37(1.79-2.98)** | | **0.59(0.54-0.69)** | | **0.73(0.61-0.87)** | **0.74(-10.5-13.39)** | |
| **Micronesia (Federated States of)** | **0.47(0.27-0.7)** | | **0.59(0.35-0.92)** | **0.25(-0.17-0.75)** | | **1.25(0.75-1.74)** | | **1.11(0.7-1.65)** | **-0.4(-8.82-8.78)** | |
| **Monaco** | **0.9(0.66-1.21)** | | **1.24(0.91-1.62)** | **0.38(-0.03-0.88)** | | **1.08(0.8-1.43)** | | **1.01(0.76-1.31)** | **-0.21(-9.12-9.57)** | |
| **Mongolia** | **2.82(1.71-4.78)** | | **6.35(4.4-10.76)** | **1.25(0.5-2.85)** | | **0.26(0.16-0.45)** | | **0.27(0.19-0.45)** | **0.1(-16.79-20.42)** | |
| **Montenegro** | **1.96(1.48-2.38)** | | **4.57(3.7-5.67)** | **1.33(0.75-2.2)** | | **0.34(0.26-0.42)** | | **0.49(0.4-0.61)** | **1.26(-12.88-17.7)** | |
| **Morocco** | **110.56(72.29-155.29)** | | **283.33(196.16-376.27)** | **1.56(0.92-2.83)** | | **0.9(0.58-1.28)** | | **1.03(0.69-1.37)** | **0.47(-8.86-10.75)** | |
| **Mozambique** | **39.01(24.56-56.39)** | | **96.93(67.04-131.18)** | **1.48(0.77-2.65)** | | **0.86(0.56-1.23)** | | **1.12(0.8-1.57)** | **0.94(-8.4-11.22)** | |
| **Myanmar** | **59.45(37.36-96.99)** | | **102.09(78.04-143.57)** | **0.72(0.13-1.72)** | | **0.29(0.18-0.47)** | | **0.25(0.19-0.34)** | **-0.54(-17.38-19.74)** | |
| **Namibia** | **4.54(3.19-6.42)** | | **10.58(7.88-14.41)** | **1.33(0.7-2.36)** | | **0.82(0.57-1.12)** | | **0.91(0.67-1.21)** | **0.35(-9.44-11.21)** | |
| **Nauru** | **0.04(0.03-0.06)** | | **0.04(0.02-0.06)** | **-0.07(-0.28-0.22)** | | **1.39(0.96-1.9)** | | **1.24(0.81-1.74)** | **-0.39(-8.37-8.28)** | |
| **Nepal** | **45.77(28.03-64.91)** | | **158.92(110.61-220.55)** | **2.47(1.4-4.26)** | | **0.65(0.41-0.94)** | | **0.89(0.61-1.27)** | **1.1(-9.44-12.85)** | |
| **Netherlands** | **813.55(668.18-1120.5)** | | **2053.9(1613.79-2411.65)** | **1.52(0.84-2.11)** | | **3.97(3.26-5.5)** | | **5.26(4.15-6.14)** | **0.97(-3.47-5.61)** | |
| **New Zealand** | **164.81(145.29-181.04)** | | **430.74(346.36-489.52)** | **1.61(1.23-1.91)** | | **4.43(3.88-4.87)** | | **4.79(3.88-5.41)** | **0.27(-4.1-4.84)** | |
| **Nicaragua** | **6(5.22-6.99)** | | **15.68(12.54-20.28)** | **1.62(1.05-2.33)** | | **0.38(0.33-0.44)** | | **0.37(0.3-0.49)** | **-0.01(-14.47-16.88)** | |
| **Niger** | **14.54(6.87-23.74)** | | **38.54(21.87-63.09)** | **1.65(0.85-3.07)** | | **0.52(0.25-0.83)** | | **0.51(0.31-0.81)** | **-0.06(-12.49-14.13)** | |
| **Nigeria** | **282.35(136.17-486.88)** | | **427.09(292.25-616.67)** | **0.51(-0.04-1.73)** | | **0.66(0.33-1.13)** | | **0.51(0.36-0.69)** | **-0.93(-12.66-12.38)** | |
| **Niue** | **0.02(0.02-0.03)** | | **0.02(0.01-0.02)** | **-0.3(-0.47--0.08)** | | **1.03(0.77-1.37)** | | **0.84(0.63-1.1)** | **-0.7(-10.11-9.7)** | |
| **North Macedonia** | **7.01(5.84-9.07)** | | **19.28(14.91-24.67)** | **1.75(1.01-2.67)** | | **0.46(0.37-0.6)** | | **0.75(0.59-0.93)** | **1.66(-10.39-15.34)** | |
| **Northern Mariana Islands** | **0.31(0.23-0.42)** | | **0.85(0.68-1.02)** | **1.7(0.87-2.78)** | | **1.99(1.54-2.53)** | | **2.67(2.08-3.26)** | **1.02(-5.18-7.63)** | |
| **Norway** | **425.94(362.99-491.19)** | | **691.2(548.31-784.6)** | **0.62(0.43-0.78)** | | **5.55(4.76-6.39)** | | **5.72(4.56-6.45)** | **0.11(-3.85-4.22)** | |
| **Oman** | **8.79(6.24-11.67)** | | **20.92(13.63-29.32)** | **1.38(0.43-2.9)** | | **1.59(1.13-2.1)** | | **1.49(1.05-1.95)** | **-0.22(-7.62-7.78)** | |
| **Pakistan** | **378.98(212.46-548.06)** | | **790.81(510.68-1071.57)** | **1.09(0.64-1.79)** | | **0.76(0.44-1.09)** | | **0.92(0.61-1.22)** | **0.64(-9.36-11.74)** | |
| **Palau** | **0.03(0.02-0.04)** | | **0.05(0.04-0.06)** | **0.79(0.27-1.63)** | | **0.27(0.2-0.38)** | | **0.25(0.2-0.33)** | **-0.23(-17.2-20.2)** | |
| **Palestine** | **2.76(1.85-4.11)** | | **7.29(5.95-9.26)** | **1.64(0.79-2.94)** | | **0.31(0.22-0.46)** | | **0.32(0.26-0.4)** | **0.03(-15.62-18.59)** | |
| **Panama** | **13.23(11.25-15.18)** | | **20.45(15.57-26.84)** | **0.55(0.15-1.11)** | | **0.88(0.74-1.01)** | | **0.49(0.37-0.64)** | **-1.99(-13.11-10.55)** | |
| **Papua New Guinea** | **9.78(4.75-16.31)** | | **26.08(15.26-40.54)** | **1.67(0.95-2.78)** | | **0.73(0.37-1.15)** | | **0.71(0.43-1.09)** | **-0.11(-10.75-11.8)** | |
| **Paraguay** | **25.37(20.43-30.13)** | | **115.65(86.37-148.6)** | **3.56(2.28-5.34)** | | **1.22(0.98-1.44)** | | **2.2(1.64-2.82)** | **2.06(-5.44-10.16)** | |
| **Peru** | **62.47(46.55-81.13)** | | **96.9(70.05-131.49)** | **0.55(-0.01-1.45)** | | **0.52(0.39-0.66)** | | **0.3(0.22-0.41)** | **-1.88(-15.97-14.58)** | |
| **Philippines** | **31.48(26.65-40.06)** | | **136.13(110.83-165.76)** | **3.32(2.15-4.36)** | | **0.14(0.12-0.19)** | | **0.2(0.16-0.24)** | **1.15(-20.04-27.94)** | |
| **Poland** | **95.86(67.2-139.42)** | | **1522.06(1121.8-1931.62)** | **14.88(7.91-25.41)** | | **0.23(0.16-0.33)** | | **2.05(1.52-2.61)** | **7.84(-7.05-25.11)** | |
| **Portugal** | **181.72(136.86-209.27)** | | **954.84(778.15-1081.4)** | **4.25(3.28-5.72)** | | **1.44(1.07-1.66)** | | **3.18(2.61-3.59)** | **2.77(-3.98-10)** | |
| **Puerto Rico** | **49.25(42.39-55.03)** | | **93.59(71.1-118.63)** | **0.9(0.46-1.45)** | | **1.43(1.23-1.62)** | | **1.14(0.87-1.45)** | **-0.78(-8.84-8)** | |
| **Qatar** | **1.19(0.89-1.62)** | | **4.6(3.18-6.61)** | **2.88(1.62-4.87)** | | **2.06(1.41-3.1)** | | **0.96(0.72-1.27)** | **-2.58(-10.38-5.9)** | |
| **Republic of Korea** | **113.29(83.63-144.27)** | | **664.47(536.44-786.9)** | **4.87(3.42-7.08)** | | **0.52(0.39-0.65)** | | **0.83(0.67-0.98)** | **1.6(-9.83-14.47)** | |
| **Republic of Moldova** | **7.27(5.87-8.32)** | | **9.2(7.24-11.7)** | **0.27(0.01-0.61)** | | **0.18(0.15-0.21)** | | **0.16(0.13-0.21)** | **-0.34(-20.93-25.6)** | |
| **Romania** | **150.46(118.02-194.03)** | | **298.74(236.3-364.34)** | **0.99(0.54-1.55)** | | **0.58(0.46-0.77)** | | **0.77(0.61-0.94)** | **0.96(-10.21-13.53)** | |
| **Russian Federation** | **403.44(315.88-450.65)** | | **1248.79(1015.32-1503.04)** | **2.1(1.48-3.23)** | | **0.24(0.19-0.27)** | | **0.54(0.44-0.66)** | **2.86(-12.86-21.42)** | |
| **Rwanda** | **30.29(17.58-50.35)** | | **47.99(34.15-68.99)** | **0.58(-0.05-1.72)** | | **1.27(0.78-1.98)** | | **1.06(0.72-1.57)** | **-0.62(-9.06-8.61)** | |
| **Saint Kitts and Nevis** | **0.45(0.34-0.55)** | | **0.59(0.48-0.75)** | **0.32(-0.03-0.83)** | | **1.26(0.97-1.53)** | | **1.09(0.91-1.36)** | **-0.48(-8.91-8.71)** | |
| **Saint Lucia** | **0.89(0.73-1.07)** | | **1.93(1.59-2.33)** | **1.18(0.7-1.76)** | | **1.1(0.9-1.31)** | | **0.94(0.78-1.13)** | **-0.52(-9.53-9.39)** | |
| **Saint Vincent and the Grenadines** | **1.14(0.9-1.31)** | | **1.39(1.16-1.72)** | **0.22(-0.01-0.66)** | | **1.61(1.27-1.84)** | | **1.11(0.93-1.37)** | **-1.27(-9.18-7.32)** | |
| **Samoa** | **0.83(0.62-1.07)** | | **1.12(0.83-1.53)** | **0.35(-0.02-0.85)** | | **1.08(0.81-1.39)** | | **0.85(0.64-1.16)** | **-0.83(-10.09-9.39)** | |
| **San Marino** | **0.6(0.45-0.76)** | | **2.04(1.32-2.91)** | **2.42(1.22-4.17)** | | **1.92(1.43-2.47)** | | **2.37(1.55-3.37)** | **0.72(-5.68-7.55)** | |
| **Sao Tome and Principe** | **0.26(0.16-0.37)** | | **0.62(0.43-0.82)** | **1.41(0.7-2.67)** | | **0.43(0.27-0.62)** | | **0.58(0.41-0.78)** | **1.02(-11.86-15.78)** | |
| **Saudi Arabia** | **28.83(17.26-40.1)** | | **97.53(73.92-132.65)** | **2.38(1.16-5.3)** | | **0.57(0.32-0.81)** | | **0.65(0.48-0.84)** | **0.43(-11.17-13.55)** | |
| **Senegal** | **15.44(9.53-21.96)** | | **37.86(25.47-53.56)** | **1.45(0.79-2.5)** | | **0.48(0.3-0.69)** | | **0.51(0.35-0.71)** | **0.2(-12.5-14.76)** | |
| **Serbia** | **117.01(80.29-148.01)** | | **443.34(339.04-574.46)** | **2.79(1.64-5.03)** | | **1.18(0.79-1.51)** | | **2.84(2.2-3.7)** | **3.08(-4.28-10.99)** | |
| **Seychelles** | **0.36(0.29-0.43)** | | **0.68(0.54-0.83)** | **0.86(0.5-1.34)** | | **0.65(0.52-0.78)** | | **0.7(0.56-0.86)** | **0.25(-10.79-12.66)** | |
| **Sierra Leone** | **9.24(5.21-14.4)** | | **20.04(12.04-30.74)** | **1.17(0.53-2.19)** | | **0.49(0.28-0.75)** | | **0.54(0.33-0.82)** | **0.37(-12.18-14.71)** | |
| **Singapore** | **14.94(13.04-17.33)** | | **35.16(27.17-42.09)** | **1.35(0.91-1.84)** | | **0.77(0.65-0.88)** | | **0.49(0.38-0.59)** | **-1.53(-12.94-11.39)** | |
| **Slovakia** | **20.71(16.92-33.07)** | | **135.1(101.43-172.4)** | **5.52(2.45-7.96)** | | **0.36(0.29-0.56)** | | **1.48(1.12-1.89)** | **5.05(-7.41-19.19)** | |
| **Slovenia** | **59.42(44.5-83.09)** | | **363.7(262.3-482.96)** | **5.12(3.34-7.53)** | | **2.6(1.95-3.59)** | | **6.77(4.92-8.94)** | **3.35(-1.62-8.57)** | |
| **Solomon Islands** | **1.03(0.53-1.76)** | | **2.26(1.18-3.82)** | **1.19(0.64-1.93)** | | **0.99(0.6-1.45)** | | **0.89(0.54-1.35)** | **-0.34(-9.71-9.99)** | |
| **Somalia** | **20.89(10.52-36.2)** | | **41.97(24.23-67.01)** | **1.01(0.42-2.09)** | | **1(0.58-1.62)** | | **0.83(0.52-1.31)** | **-0.63(-10.13-9.87)** | |
| **South Africa** | **178.76(151.09-204.91)** | | **348.55(283.57-394.58)** | **0.95(0.67-1.24)** | | **0.93(0.78-1.07)** | | **0.96(0.79-1.09)** | **0.12(-9.26-10.48)** | |
| **South Sudan** | **17.26(10.68-26.4)** | | **19.82(13.1-28.8)** | **0.15(-0.25-0.76)** | | **0.84(0.53-1.23)** | | **0.67(0.44-0.98)** | **-0.78(-11.17-10.81)** | |
| **Spain** | **1686.4(1484.28-1906.04)** | | **5153.06(4177-6116.47)** | **2.06(1.62-2.55)** | | **3.21(2.81-3.65)** | | **4(3.3-4.68)** | **0.76(-4.21-5.99)** | |
| **Sri Lanka** | **89.37(75.4-105.93)** | | **151.83(110.77-205.59)** | **0.7(0.2-1.47)** | | **0.93(0.78-1.1)** | | **0.63(0.46-0.86)** | **-1.32(-11.62-10.18)** | |
| **Sudan** | **78.79(44.76-118.73)** | | **162.98(107.71-242.36)** | **1.07(0.37-2.41)** | | **0.88(0.51-1.3)** | | **0.88(0.59-1.27)** | **0.01(-9.66-10.73)** | |
| **Suriname** | **2.02(1.68-2.37)** | | **4.78(3.86-5.94)** | **1.37(0.88-2.03)** | | **0.81(0.67-0.95)** | | **0.85(0.68-1.06)** | **0.2(-9.79-11.29)** | |
| **Sweden** | **676.7(596.45-740.23)** | | **1209.17(979.36-1405.81)** | **0.79(0.59-1.02)** | | **4.01(3.53-4.38)** | | **4.39(3.63-5.06)** | **0.31(-4.26-5.11)** | |
| **Switzerland** | **391.83(318.4-460.31)** | | **746.29(581.12-880.27)** | **0.9(0.62-1.27)** | | **3.39(2.76-3.98)** | | **3.18(2.52-3.71)** | **-0.22(-5.35-5.19)** | |
| **Syrian Arab Republic** | **153.1(102.18-210.98)** | | **186.43(129.15-268.47)** | **0.22(-0.18-0.88)** | | **3.12(2.02-4.21)** | | **1.81(1.28-2.57)** | **-1.85(-7.86-4.55)** | |
| **Taiwan (Province of China)** | **69.19(57.52-99.21)** | | **370.2(279.7-471.91)** | **4.35(2.25-6.22)** | | **0.64(0.52-0.92)** | | **0.91(0.69-1.16)** | **1.23(-9.34-13.04)** | |
| **Tajikistan** | **4.48(3.46-5.72)** | | **9.76(7.74-12.19)** | **1.18(0.57-1.97)** | | **0.16(0.12-0.21)** | | **0.21(0.17-0.27)** | **1.06(-19.23-26.44)** | |
| **Thailand** | **89.28(70.82-111.48)** | | **360.43(264.09-472.85)** | **3.04(1.75-4.8)** | | **0.29(0.23-0.36)** | | **0.37(0.27-0.49)** | **0.88(-14.69-19.29)** | |
| **Timor-Leste** | **0.41(0.27-0.68)** | | **1.7(1.11-2.59)** | **3.13(1.64-5.46)** | | **0.18(0.12-0.29)** | | **0.24(0.16-0.36)** | **0.93(-18.13-24.43)** | |
| **Togo** | **7.24(4.32-11.25)** | | **22.85(13.38-37.01)** | **2.16(1.25-3.58)** | | **0.57(0.34-0.87)** | | **0.63(0.39-0.98)** | **0.35(-11.35-13.6)** | |
| **Tokelau** | **0.01(0.01-0.02)** | | **0.01(0.01-0.01)** | **-0.26(-0.46-0.1)** | | **0.95(0.63-1.34)** | | **0.8(0.61-1.09)** | **-0.6(-10.27-10.13)** | |
| **Tonga** | **0.4(0.31-0.53)** | | **0.59(0.43-0.8)** | **0.48(0.08-1.07)** | | **0.86(0.65-1.15)** | | **0.76(0.55-1.02)** | **-0.46(-10.51-10.73)** | |
| **Trinidad and Tobago** | **5.36(4.42-6.45)** | | **10.41(7.85-13.61)** | **0.94(0.42-1.71)** | | **0.71(0.57-0.84)** | | **0.6(0.45-0.78)** | **-0.56(-11.69-11.96)** | |
| **Tunisia** | **42.96(29.23-55.4)** | | **107.99(69.15-153.7)** | **1.51(0.74-2.68)** | | **0.96(0.65-1.24)** | | **0.92(0.59-1.31)** | **-0.16(-9.54-10.19)** | |
| **Turkey** | **466.98(301-632.57)** | | **851.22(632.49-1092.68)** | **0.82(0.25-2.1)** | | **1.45(0.93-2.01)** | | **1.03(0.76-1.32)** | **-1.17(-9.42-7.83)** | |
| **Turkmenistan** | **2.71(2.12-3.18)** | | **7.02(5.37-9.26)** | **1.59(0.92-2.62)** | | **0.15(0.11-0.18)** | | **0.19(0.15-0.25)** | **0.84(-20.18-27.41)** | |
| **Tuvalu** | **0.06(0.04-0.08)** | | **0.08(0.06-0.11)** | **0.38(-0.02-1.1)** | | **1.09(0.69-1.54)** | | **0.93(0.66-1.29)** | **-0.55(-9.6-9.4)** | |
| **Uganda** | **44.66(31.95-65.35)** | | **101.55(74.99-138.75)** | **1.27(0.62-2.22)** | | **0.84(0.61-1.22)** | | **0.89(0.63-1.26)** | **0.22(-9.58-11.08)** | |
| **Ukraine** | **98.69(73.81-118.15)** | | **185.21(154.47-221.29)** | **0.88(0.49-1.47)** | | **0.15(0.11-0.17)** | | **0.25(0.21-0.3)** | **1.95(-18.38-27.34)** | |
| **United Arab Emirates** | **6.39(3.81-9.47)** | | **51.58(26.75-79.78)** | **7.08(4.16-11.9)** | | **1.21(0.72-1.78)** | | **0.95(0.52-1.47)** | **-0.82(-9.59-8.81)** | |
| **United Kingdom** | **2981.82(2595.03-3184.44)** | | **5408.66(4572.07-5893.47)** | **0.81(0.62-1.05)** | | **3.2(2.78-3.42)** | | **3.62(3.09-3.94)** | **0.43(-4.64-5.78)** | |
| **United Republic of Tanzania** | **99.09(73.09-143.96)** | | **213.95(146.12-303.32)** | **1.16(0.44-2.33)** | | **1.13(0.83-1.59)** | | **1.06(0.7-1.49)** | **-0.2(-8.92-9.34)** | |
| **United States of America** | **12384.82(10877.47-13503.78)** | | **24825.95(20353.87-27717.51)** | **1(0.84-1.14)** | | **3.66(3.22-3.99)** | | **3.73(3.11-4.12)** | **0.06(-4.79-5.16)** | |
| **United States Virgin Islands** | **1.45(1.16-1.77)** | | **3.48(2.87-4.18)** | **1.41(0.92-2.04)** | | **1.94(1.57-2.4)** | | **2.05(1.7-2.49)** | **0.19(-6.37-7.21)** | |
| **Uruguay** | **137.09(120.3-152.92)** | | **342.85(286.93-391.79)** | **1.5(1.1-1.95)** | | **3.62(3.15-4.03)** | | **5.18(4.38-5.88)** | **1.24(-3.34-6.04)** | |
| **Uzbekistan** | **11.37(6.38-18.44)** | | **67.74(49.95-95.55)** | **4.96(2.3-11.27)** | | **0.1(0.06-0.17)** | | **0.46(0.33-0.66)** | **5.26(-16.61-32.87)** | |
| **Vanuatu** | **0.5(0.31-0.73)** | | **1.28(0.83-1.87)** | **1.56(0.9-2.53)** | | **0.99(0.67-1.35)** | | **0.89(0.58-1.23)** | **-0.37(-9.74-9.97)** | |
| **Venezuela (Bolivarian Republic of)** | **88.42(71.47-100.05)** | | **270.12(201.86-364.14)** | **2.05(1.19-3.36)** | | **0.94(0.76-1.08)** | | **0.97(0.72-1.3)** | **0.09(-9.24-10.38)** | |
| **Viet Nam** | **118.43(90-167.41)** | | **244.66(178.52-340.87)** | **1.07(0.51-1.95)** | | **0.32(0.24-0.45)** | | **0.29(0.22-0.41)** | **-0.31(-16.11-18.47)** | |
| **Yemen** | **41.48(21.87-67.05)** | | **118.51(80.2-167.54)** | **1.86(0.95-3.49)** | | **0.92(0.51-1.41)** | | **0.93(0.64-1.28)** | **0.01(-9.45-10.47)** | |
| **Zambia** | **19.8(12.29-29.39)** | | **66.6(49.01-89.89)** | **2.36(1.36-4.09)** | | **0.88(0.58-1.24)** | | **1.27(0.95-1.67)** | **1.27(-7.8-11.23)** | |
| **Zimbabwe** | **24.52(18.93-35.12)** | | **48.54(35.26-68.86)** | **0.98(0.45-1.67)** | | **0.78(0.58-1.13)** | | **0.9(0.65-1.28)** | **0.49(-9.51-11.59)** | |
| **Abbreviations: CAVD, calcific aortic valve disease; EAPC, estimated annual percentage change; ASDR, age standardized death rate; UI, uncertainty interval; CI, confidence interval.** | | | | | | | | | | |
